# Synthesis of Electrophiles Derived from Dimeric Aminoboranes and Assessing Their Utility in the Borylation of π Nucleophiles

**DOI:** 10.1021/acs.organomet.2c00393

**Published:** 2022-09-14

**Authors:** Clément R. P. Millet, Jürgen Pahl, Emily Noone, Kang Yuan, Gary S. Nichol, Marina Uzelac, Michael J. Ingleson

**Affiliations:** School of Chemistry, University of Edinburgh, Edinburgh EH9 3FJ, U.K.

## Abstract

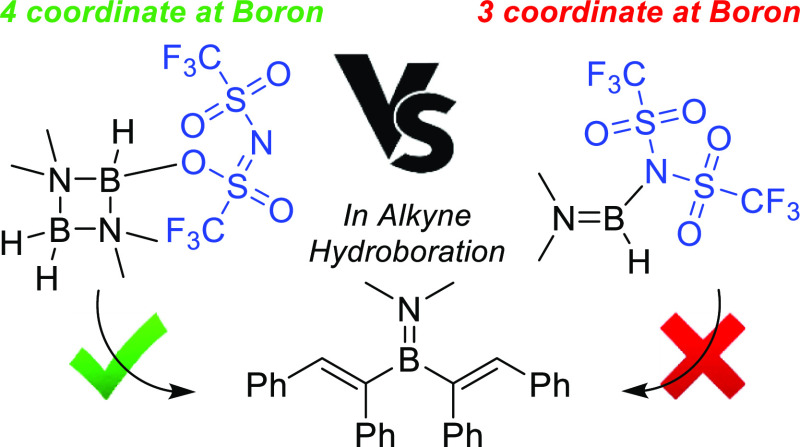

Dimeric aminoboranes, [H_2_BNR_2_]_2_ (R = Me or CH_2_CH_2_) containing B_2_N_2_ cores, can be activated by I_2_, HNTf_2_ (NTf_2_ = [N(SO_2_CF_3_)_2_]), or [Ph_3_C][B(C_6_F_5_)_4_] to form isolable H_2_B(μ-NR_2_)_2_BHX (for X = I or NTf_2_). For X = [B(C_6_F_5_)_4_]^−^ further reactivity, presumably between [H_2_B(μ-NMe_2_)_2_BH][B(C_6_F_5_)_4_] and aminoborane, forms a B_3_N_3_-based monocation containing a three-center two electron B-(μ-H)-B moiety. The structures of H_2_B(μ-NMe_2_)_2_BH(I) and [(μ-NMe_2_)BH(NTf_2_)]_2_ indicated a sterically crowded environment around boron, and this leads to the less common O-bound mode of NTf_2_ binding. While the iodide congener reacted very slowly with alkynes, the NTf_2_ analogues were more reactive, with hydroboration of internal alkynes forming (vinyl)_2_BNR_2_ species and R_2_NBH(NTf_2_) as the major products. Further studies indicated that the B_2_N_2_ core is maintained during the first hydroboration, and that it is during subsequent steps that B_2_N_2_ dissociation occurs. In the mono-boron systems, for example, ^*i*^Pr_2_NBH(NTf_2_), NTf_2_ is N-bound; thus, they have less steric crowding around boron relative to the B_2_N_2_ systems. Notably, the monoboron systems are much less reactive in alkyne hydroboration than the B_2_N_2_-based bis-boranes, despite the former being three coordinate at boron while the latter are four coordinate at boron. Finally, these B_2_N_2_ electrophiles are much more prone to dissociate into mono-borane species than pyrazabole [H_2_B(μ-N_2_C_3_H_3_)]_2_ analogues, making them less useful for the directed diborylation of a single substrate.

## 1 Introduction

**Figure 1:**
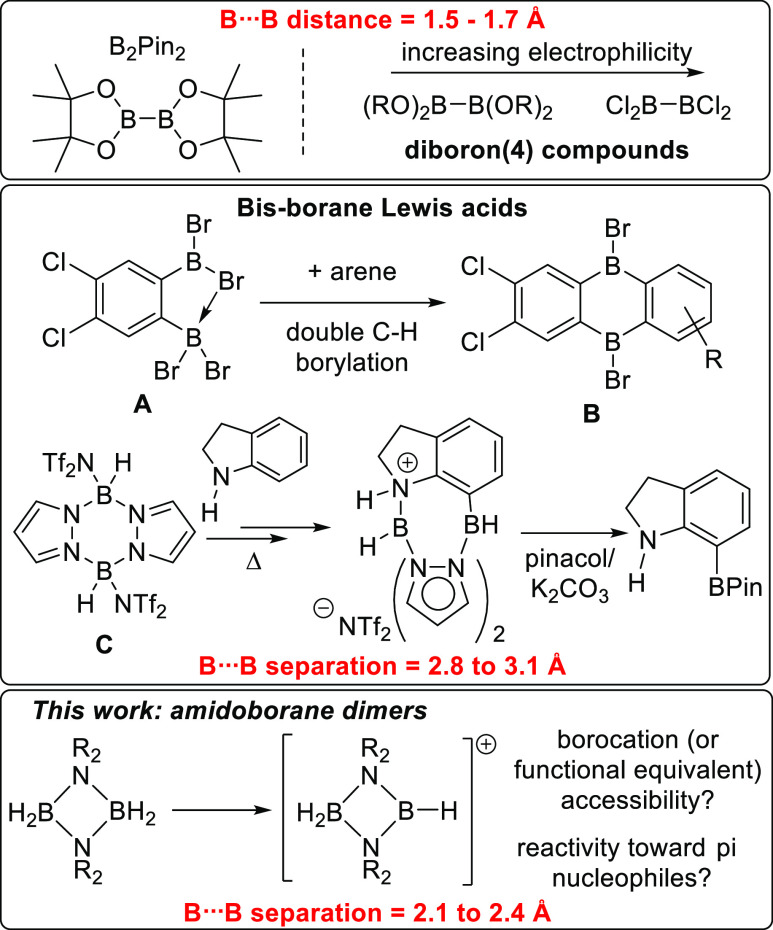
Top, diboron(4) compounds. Middle, bis-boranes able to effect double E–H borylation (E = N or C). Bottom, this work exploring electrophiles derived from dimeric aminoboranes.

Diboron compounds are of significant importance in synthesis, particularly through the use of tetra-alkoxydiboron(4)s, such as B_2_Pin_2_ (Figure 1, top), in transition-metal-catalyzed borylation reactions.^1^ Recent years have seen a resurgence in the chemistry of more (relative to B_2_Pin_2_) electrophilic diboron(4) compounds. This has expanded on the work of Schlesinger using B_2_X_4_ (X = halide),^2^ and a number of electrophilic diboron(4) compounds now have been reported, including examples that can borylate π nucleophiles and activate small molecules (e.g., H_2_ and CO).^3^ Parallel to this, there has been significant research into the chemistry of bidentate Lewis acids containing two electrophilic boron centers but no B–B bond, herein termed bis-boranes. While bis-boranes have been widely applied for small-molecule activation [e.g., in frustrated Lewis pairs (FLPs)]^4^ and in anion sensing,^5^ the use of bis-boranes in the double borylation of π nucleophiles is relatively underexplored (*vide infra*).^6^ This is despite the tunable nature of bis-boranes, particularly tailoring the B···B separation to match a specific substrate.

One of the most utilized classes of bis-boranes are 1,2-C_6_H_4_(BX_2_)_2_ (X = Cl or Br) and derivatives (e.g., 9,10-diboraanthracenes). While various groups demonstrated that these bis-borane ditopic Lewis acids can be used for small-molecule activation^7^ and anion binding,^5^ Wagner et al. demonstrated that they can be used for double electrophilic C–H borylation.^8^ Specifically, compound **A** effected the double vicinal electrophilic C–H borylation of a range of aromatics to form B_2_-doped polycyclic aromatic hydrocarbons (e.g., **B**). We recently extended this approach using bis-borane Lewis acids based on pyrazaboles (e.g., **C**),^9^ which enabled the borylation-directed borylation of indoles and indolines (Figure 1, middle). Notably, the pyrazabole B_2_N_4_ core in **C** is sufficiently robust to persist during both electrophilic borylation steps (N–H and C–H), but it is reactive enough that it can be transformed subsequently into synthetically ubiquitous pinacol boronate esters. The B···B separation in **A** and **C** is ca. 3 Å, which is ideal for the vicinal functionalization of aromatics and the N/C7 functionalization of indoles. However, bis-borane Lewis acids with smaller B···B separations will be required for other substrates, for example, for accessing peri diborylated naphthalenes or 1,1-diborylated alkenes [where B···B separations in the double borylation products containing a B-(μ-NMe_2_)-B unit will be ca. 2.1–2.5 Å].^10,11^ Thus, we were interested in accessing electrophiles derived from aminoborane dimers, (H_2_BNR_2_)_2_, which have appropriate B···B separations and are simple to access. Herein, we report the synthesis, characterization, and reactivity studies toward π nucleophiles of a series of boron electrophiles derived from aminoborane dimers.

## 2 Results and Discussion

**Figure 2:**
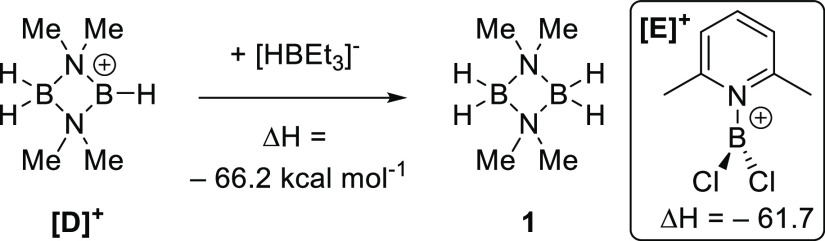
HIA calculations (relative to BEt_3_) at M06-2x/6-311G(d,p)//PCM(DCM) (all calculations herein are performed at this level).

Aminoboranes, R_2_NBH_2_, often exist in an equilibrium between dimeric and monomeric forms.^12^ As a consequence of this sterically driven equilibrium, only a small number of aminoboranes are accessible as stable dimers in solution. Aminoboranes [Me_2_NBH_2_]_2_, **1**, and [(pyrrolidine)BH_2_]_2_, **2**, were selected due to their inexpensive starting materials (Me_2_NH/pyrrolidine and L-BH_3_) and their dimeric form dominating in solution at room temperature (and even on heating). Importantly, solid state data for **1** and **2** show that they exhibit B···B separations of ca. 2.20 Å, which is in the desired region.^13^ Furthermore, calculations on the hydride ion affinity (HIA) of a borenium cation derived from **1**,^14^**[D]**^**+**^, reveal it to have a high HIA. Indeed, the HIA of **[D]**^**+**^ is comparable to some of the most reactive (in electrophilic C–H borylation)^15^ mono-boron cationic species (e.g., [**E]**^**+**^, Figure 2, inset) and greater than the HIA for borocations derived from pyrazabole.^9^ Presumably, the high HIA for **[D]**^**+**^ is due to the absence of any π donors bound to boron and indicates that if borenium cations (or functional borenium equivalents)^14^ can be accessed from **1** or **2**, they will be highly reactive species.

### 2.1 Synthesis of Electrophilic “B_2_N_2_” Aminoboranes

**Figure 3:**
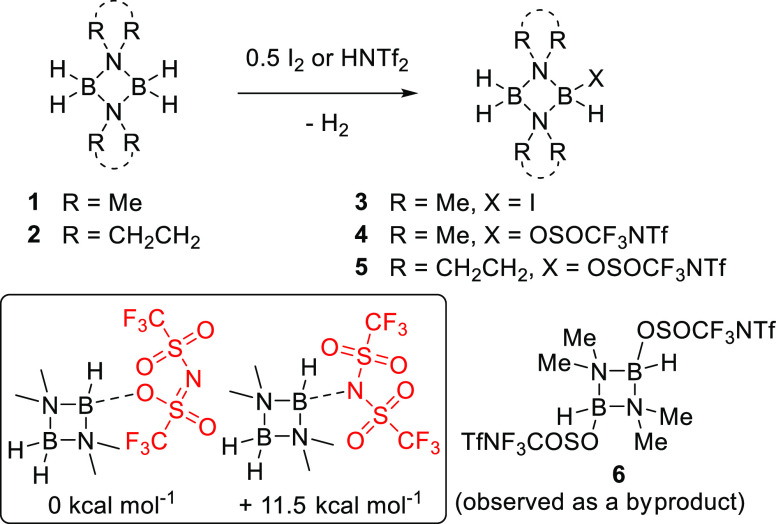
Top, the synthesis of compounds **3–5**. Inset bottom, the relative energies (Δ*G*) of O- and N-bound NTf_2_ isomers of **4**. Bottom right, compound **6**, only isolated in our hands as a byproduct from the reaction between alkynes and **4** (*vide infra*).

Aminoboranes **1** and **2** were synthesized via catalyzed dehydrocoupling of the parent amine-borane following a reported method with lithium 2-^*t*^Bu-pyridine.^16^ A series of boron electrophiles derived from these precursors were targeted next. First **1** was treated with 0.5 equiv iodine (Figure 3, top), resulting in immediate hydrogen evolution and formation of H_2_B(μ-Me_2_N)_2_BH(I), **3**. Compound **3** was isolated by sublimation as a crystalline solid in 34% yield (though *in situ* conversion to **3** is effectively quantitative). The ^11^B NMR spectrum of **3** revealed two distinct boron signals at δ_11B_ 3.9 (t, ^1^*J*_BH_ = 118 Hz, *B*H_2_) and 0.4 (d, ^1^*J*_BH_ = 142 Hz, *B*HI), in accordance with the two different boron environments and their respective mono- and di-hydride substitution. The three inequivalent hydrides on the two boron centers were also observed by ^1^H NMR spectroscopy (see Figure S10). X-ray diffraction (XRD) analysis confirmed the structure of **3** (*vide infra*), while IR spectroscopy confirmed only terminal B–H units. Notably, attempts to access a bis iodide derivative, [Me_2_NBH(I)]_2_, by adding excess iodine to **1** failed even after 3 days at room temperature. While heating did lead to slow further reactivity of **3** with I_2_, this led to complex mixtures which contained monomeric species, for example, (amine)BI_3_.

Mono and bis-borane systems, involving boron hydrides in combination with HNTf_2_ (NTf_2_ = N(S(O)_2_CF_3_)_2_), have been used to generate reactive boron electrophiles^17^ including examples which can effect C–B formation.^9,18^ Thus, aminoboranes **1** and **2** were treated with one equivalent of HNTf_2_ (Figure 3, top). Hydrogen evolution was observed, and H_2_B(μ-Me_2_N)_2_BH(OSOCF_3_NTf), **4**, and H_2_B(μ-pyrrolidine)_2_BH(OSOCF_3_NTf), **5**, were isolated in good yields (83 and 65%, respectively). The ^11^B NMR spectrum of **4** displayed two signals at δ_11B_ 4.9 (d, ^1^*J*_BH_ = 142 Hz, (H)*B*OSOCF_3_NTf) and 3.3 (t, ^1^*J*_BH_ = 119 Hz, *B*H_2_). The ^11^B NMR signals of **5** were overlapped, giving a multiplet (δ_11B_ 5.4–1.1), that turned into two singlets in the ^11^B{^1^H} NMR spectrum (δ_11B_ 3.5 and 2.8) (see Figures S22 and S23). The ^19^F NMR spectra of both **4** and **5** showed two fluorine resonances (**4** δ_19F_ = −75.7 and −78.5; **5** δ_19F_ = −75.7 and −78.4), indicating inequivalent CF_3_ groups in the NTf_2_ moiety. Similar ^19^F NMR spectra have been reported by Vedejs and co-workers for R_3_N–BH_2_NTf_2_ compounds where two fluorine resonances for B-NTf_2_ corresponded to the O-bound NTf_2_ isomer, in contrast to the more commonly found N-bound.^19^ The mode of NTf_2_ binding to boron is linked closely to the steric environment around boron, for example, Me_3_N–BH_2_NTf_2_ has predominantly N-bound NTf_2_ (7:1 N/O bound), while with ^*i*^Pr_2_NEt–BH_2_NTf_2_, which contains a much larger amine, it is almost exclusively O-bound NTf_2_. The existence of only O-bound NTf_2_ in **4** and **5** suggests a relatively sterically encumbered boron center due to the four flanking methyl/CH_2_ units. The complete absence (by ^19^F NMR spectroscopy) of any N-bound isomer is also consistent with calculations which determined that the N-bound isomer of **4** to be 11.5 kcal mol^–1^ higher in energy than the O-bound isomer (Figure 3, inset). While multiple crystallization attempts for both **4** and **5** were unsuccessful, all data are consistent with NTf_2_ being O-bound in both **4** and **5**, and IR spectroscopy was consistent with the presence of only terminal B–H units.

In an attempt to access a bis-NTf_2_ analogue, [(μ-Me_2_N)BH(OSOCF_3_NTf)]_2_, **6** (Figure 3, bottom right), compound **1** reacted with two equivalents of HNTf_2_. While this led to rapid formation of **4**, further reactivity was only observed on heating. This led to slow consumption of **4** to form a complex mixture; notably, this contained mono-boron species (e.g., δ_11B_ = 27.1, d, ^1^*J*_BH_ = 170 Hz—see Figure S80). It was not possible to isolate pure doubly activated diboron aminoboranes from these reactions for use in synthetic studies. However, it should be noted that **6** is accessible as it was isolated in very low quantity by fractional crystallization during reactivity studies between alkynes and **4** (*vide infra*) and was crystallographically characterized (*vide infra* for discussion).

**Figure 4:**
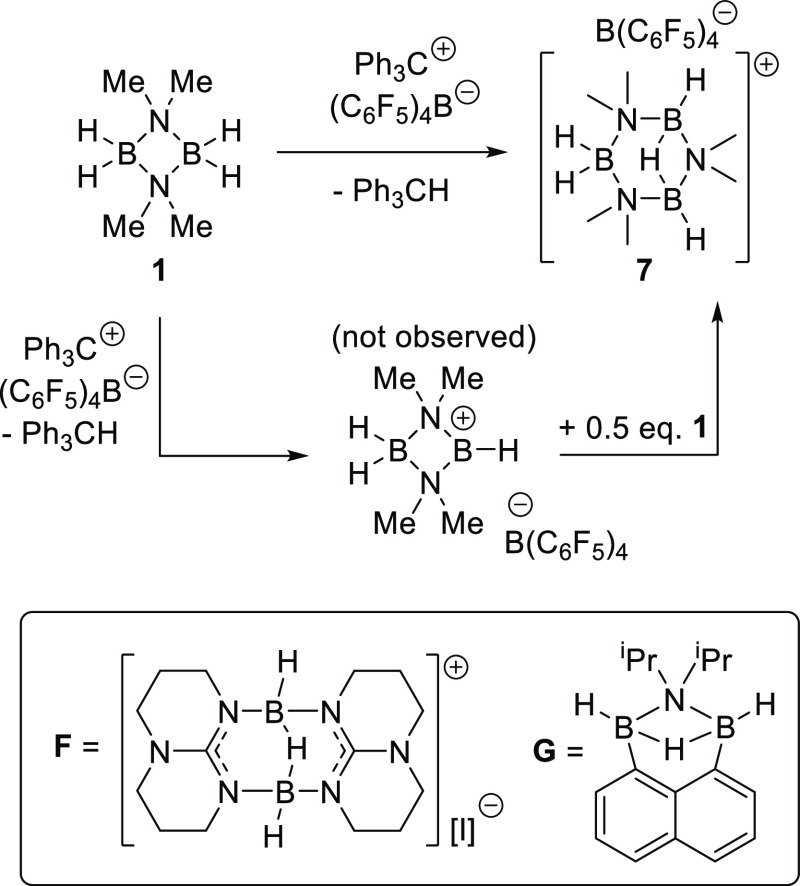
Top, synthesis of the cation **7**. Inset bottom, reported compounds with a comparable H–B(μ–H)B–H unit.

Desirous of synthesizing a bis-borane aminoborane electrophile that exists as a separated ion pair with a planar three coordinate borocation, **1** was treated with one equivalent of [Ph_3_C][B(C_6_F_5_)_4_] (Figure 4). The major product from this reaction, **7**, could be isolated by crystallization from layering a chlorobenzene solution with hexane. XRD analysis revealed the unexpected formation of the triboron monocation **7** (Figure 4, *vide infra* for structural discussion). The solid-state structure showed the formation of a salt, with a [B(C_6_F_5_)_4_]^−^ counter anion, but it was not the desired cation **[D]**^**+**^. Instead, the dimeric aminoborane **1** converted into a triboron species, containing a three-center two-electron B–H–B unit. The difference observed between forming **7** (with [B(C_6_F_5_)_4_]^−^) and **3–5** (with I^–^ or [NTf_2_]^−^) is attributed to the very weakly coordinating nature of [B(C_6_F_5_)_4_]^−^, which provides insufficient stabilization of the borocation **[D]**^**+**^.^20^ Therefore, post hydride abstraction by Ph_3_C^+^, borocation **[D]**^**+**^ reacts further, presumably with remaining **1** through formal transfer of one Me_2_NBH_2_ unit from **1** to **[D]**^**+**^. The additional Me_2_NBH_2_ formally inserts into the N–BH^+^ bond of **[D]**^**+**^, ultimately affording **7**. With the composition of **7** determined, modification of the stoichiometry to 3 equiv **1**:2 equiv [Ph_3_C][B(C_6_F_5_)_4_] enabled **7** to be formed cleanly (by *in situ* NMR spectroscopy) and isolated in 70% yield.

Analysis of **7** by 2D and low-temperature NMR experiments enabled assignment of the ^1^H and ^13^C signals, which were in accordance with the solid-state structure, with the presence of the three different hydride environments confirmed by 2D ^11^B–^1^H HMQC (see Figure S33). The bridging hydride was significantly more shielded compared to the other hydrides in **7**, coming as a broad multiplet at δ 1.72–1.10 (vs 3.45–2.14 and 2.83–1.74 for the terminal hydrides). This chemical shift is in a similar region to that for the bridging hydride in the cation **F** reported by Himmel and co-workers (δ_1H_ 1.97, in C_6_D_6_).^21^ Notably, the ^11^B NMR spectrum of **7** showed a triplet (δ_11B_ 3.4, ^1^*J*_BH_ = 120 Hz, *B*H_2_) and a doublet (δ_11B_ −6.1, ^1^*J*_BH_ = 164 Hz, H*B*(H)*B*H), with no coupling between the bridging hydride and the boron atoms observed. The absence of observable coupling with the bridging hydride in the ^11^B NMR spectrum is consistent with related literature examples (e.g., **F** and **G**—Figure 4, inset bottom)^11,21^ and is partly due to the smaller magnitude of ^1^*J*_B–H_ coupling involving bridging hydrides (as reported in these related systems). While the additional B–H coupling was observed in higher-temperature NMR experiments for **G**, coupling with the bridging hydride was not observed at higher temperature for **7**, with decomposition of **7** occuring at higher temperatures.

**Scheme 1:**
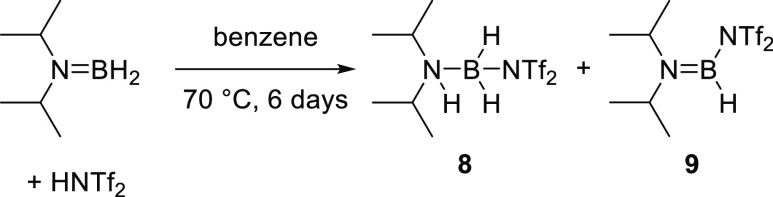
Synthesis of Electrophiles by Addition of HNTf_2_ to a Monomeric Aminoborane

Finally, to enable comparisons during reactivity studies, a strong electrophile derived from a monomeric aminoborane was targeted. Therefore, the more sterically encumbered aminoborane (^*i*^Pr)_2_NBH_2_ (which exists as a monomer at room temperature) was reacted with HNTf_2_ (Scheme 1). This reaction resulted in the immediate formation of the amine-borane adduct **8** instead of the desired product **9**, as evidenced by a boron resonance at δ_11B_ −9.5 (br, t, ^1^*J*_BH_ = 123 Hz) and was confirmed further by ^1^H, ^13^C, and ^19^F NMR spectroscopies (see Figures S40–S45). Heating **8** in benzene resulted in very slow conversion to **9**, which showed a signal in the ^11^B NMR spectrum at δ 28.9 (d, ^1^*J*_BH_ = 170 Hz); however, even after 7 days heating, a significant amount of **8** (ca. 60%) persisted. Therefore, compound **9** was isolated by fractional crystallization and characterized by ^1^H, ^13^C, and ^19^F NMR spectroscopies. The single signal in the ^19^F NMR spectrum (δ −73.2) indicated an N-bound triflimide, which was later confirmed by XRD analysis (*vide infra* for discussion). The switch from O-bound NTf_2_ in the bis-borane structures of **4** and **5** to N-bound NTf_2_ in **9** is notable and demonstrates the steric crowding present in dimeric aminoborane derivatives. Finally, it should be noted that resonances very close to the δ_11B_ of **8** (−6.5, t) and **9** (27.1, d) are observed in the reaction of **4** with HNTf_2_, supporting the conclusion that monomeric boranes are being produced from combinations of **4** and two equiv HNTf_2_.

### 2.2 Solid-State Structures

**Figure 5:**
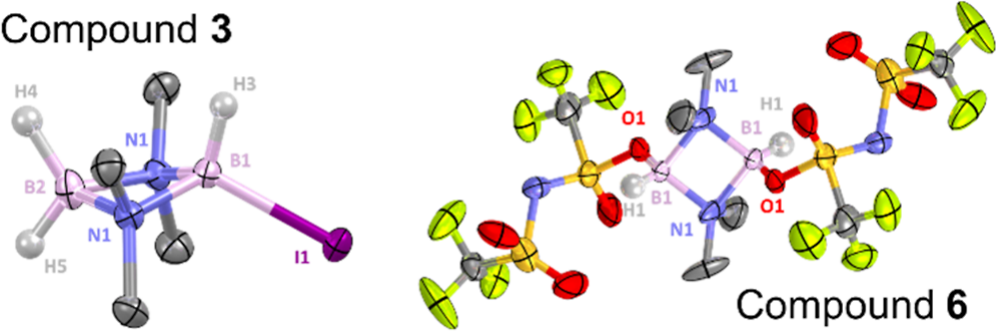
Left, the solid-state structure of **3** (50% ellipsoid probability). Hydrogen atoms except H3, H4, and H5 are omitted for clarity. Selected metrics [Å or °] for **3**: B···B = 2.142(5), B1–N1 = 1.579(3), B2–N1 = 1.614(4), B1–I1 = 2.252(4), and B2–B1–I1 = 142.9(2). Right, the solid-state structure of **6** (30% ellipsoid probability). Hydrogen atoms except H1 and disorder are omitted for clarity. Selected metrics [Å or °] for **6**: B···B = 2.274(2), B1–N1 = 1.589(8), B1–N1 = 1.662(10), B1–O1 = 1.526(7), B1–O1–S1 = 123.3(4), and B1–B1–O1 = 111.8(4).

Crystals of **3** were obtained by sublimation and confirmed the formulation from NMR spectroscopy (Figure 5, left). The B···B distance in **3** (2.142(5) Å) is slightly shorter compared to its precursor **1**. Contrary to **1** that exhibits a planar N_2_B_2_ four-membered ring (Σ (internal angles of the N_2_B_2_ ring) = 360°), the unsymmetric substitution in **3** has distorted the N_2_B_2_ ring slightly into a butterfly conformation with the Σ (internal angles of the N_2_B_2_ ring) = 355°. A similar minor distortion was observed for an unsymmetric analogue, Br_2_B(μ-Me_2_N)_2_BBr(OEt) (Σ (internal angle of the N_2_B_2_ ring) = 357°).^22^ The larger iodine atom also causes other distortions in **3**, as evidenced by the large B2–B1–I1 angle of 142.9(2)°, a value greater than those in related substituted B_2_N_2_ compounds (where angles span the range 128–139°).^22,23^ Furthermore, the incorporation of the less coordinating iodine leads to a shortening of the B1–N1 bond length (1.579(3) Å) and elongation of the B2–N1 bond length (1.614(4) Å), compared with its precursor **1**, where all the B–N bond lengths are 1.595–1.597 Å. The boron atom substituted with iodine shows a distorted tetrahedral geometry with a B–I bond of 2.252(4) Å, a slightly shorter distance compared with the B–I bond lengths reported for closely related compounds L-BH_2_I (2.30–2.34 Å, L = N-heterocyclic carbenes or PR_3_).^24^

The bis-NTf_2_ derivative, [(μ-Me_2_N)BH(OSOCF_3_NTf)]_2_, **6** (Figure 5, right) was obtained as a byproduct from reactions between **4** and alkynes by fractional crystallization. Its solid-state structure revealed doubly O-bound triflimide moieties, which is in accordance with the O- versus N-bound equilibrium being sterically driven and the expected steric encumbrance around the core N_2_B_2_ ring (based on NMR data for **4**). **6** crystallizes as the trans isomer, which exhibits a large B1–O1–S1 angle (123.3(4)°) and a B1–O1 bond length of 1.526(7) Å, which is the shortest reported distance for an NTf_2_ O bound to four coordinate boron.^17c,25^ Note, the calculated B–O distance in **4** is comparable at 1.522 Å. Finally, the B···B distance in **6** (2.274(2) Å) is larger compared to **1** and **3**, possibly due to steric effects from the two NTf_2_ units. While the four-membered ring of **6** is planar (Σ(internal angles) = 360°), the O-bound triflimides are impacting the core N_2_B_2_ ring with a noticeable difference observed between the B1–N1 bond lengths [1.589(8) and 1.662(10) Å], a difference larger than that in compound **3**.

To assess why N-binding of NTf_2_ to the B_2_N_2_ cores in **4** is significantly higher in energy, the calculated structure of the non-observed N-bound isomer of **4**, **4-N**, was analyzed. The B–NMe_2_ bonds (1.575 and 1.586 Å) are typical for boron-nitrogen single bonds at tetra-coordinate boron centers; however, the B–NTf_2_ (1.626 Å) bond length is longer than the B-NTf_2_ distance in a NTf_2_ derivative of pyrazabole [1.609(2) Å] and a Ar_3_P–BH(R)NTf_2_ species,^17b^ suggesting a weaker interaction in **4-N**. The N_2_B_2_ four-membered ring of **4-N** is found in a butterfly conformation and is more distorted than that in the case of **3**, with a Σ (internal angles of the N_2_B_2_ ring) = 351° (vs 355° in **3**). Most notably, a very large B–B-NTf_2_ angle of 149.8° is found for **4-N**, much larger than the B1–B1–O1 angle observed in compound **6** (111.8(4)°) and even higher than that the B2–B1–I1 for **3** (142.9(2)°). These data highlight the steric effects that N-binding of NTf_2_ to B_2_N_2_ cores imparts which is the likely reason why O-bound NTf_2_ is observed experimentally in **4–6**.

**Figure 6:**
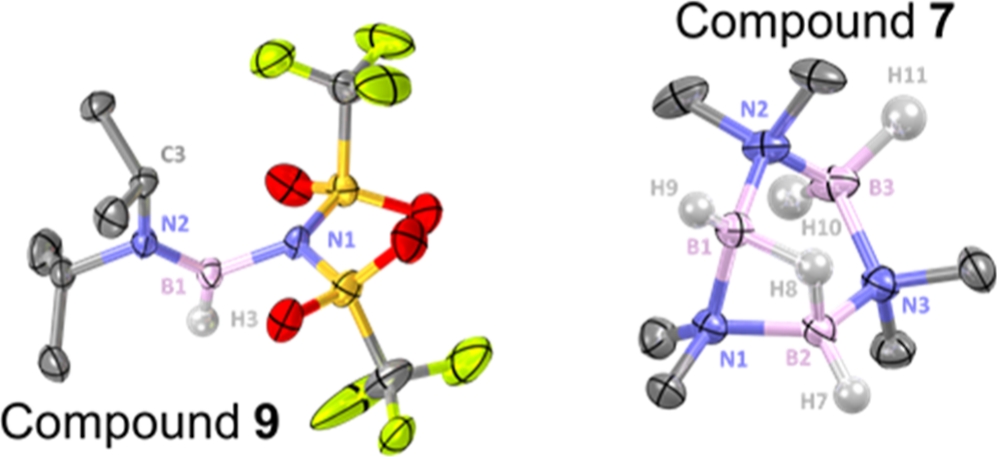
Left, the solid-state structure of **9** (50% ellipsoid probability). Hydrogen atoms except H3 are omitted for clarity. Selected metrics (Å or °) for **9**: B1–N1 = 1.566(4), B1–N2 = 1.370(4), and C3–N2–B1–N1 = −2.5(4). Right, the solid-state structure of **7** (50% ellipsoid probability). Hydrogen atoms except H7, H8, H9, H10, and H11 and the anion are omitted for clarity. Selected metrics (Å) for **7**: B1–N1 = 1.546(3), B1–N2 = 1.544(3), B2–N1 = 1.545(3), B2–N3 = 1.545(3), B3–N3 = 1.617(3), and B3–N2 = 1.617(3).

Comparison of the calculated structure of **4-N** with the solid-state structure of the **9** is also informative as **9** also contains an N-bound NTf_2_ (Figure 6, left). This is in accordance with a less hindered tricoordinate boron center. Notably, in **9**, the B1–NTf_2_ exhibits a bond length of 1.586(15) Å, which is much shorter than that calculated for **4-N**, further indicating the significant steric crowding around the B_2_N_2_ cores. Furthermore, in **9**, a short B1–N2 bond length (1.351(17) Å) is observed, which is in the range of B=N double bonds (1.3–1.4 Å),^26^ but it is shorter than previously reported R_2_N=BH_2_ species (e.g., 1.380(2) Å),^27^ possibly due to enhanced electrophilicity at boron on substitution of H for NTf_2_. The presence of a B=N unit in **9** is also confirmed by the very small C3–N2–B1–N1 torsion angle of −2.5(4)°.

Finally, the solid-state structure of **7** (Figure 6, right for the cationic component) represents a salt derived from the trimer (BH_2_)_3_(NMe_2_)_3_.^28^ Contrary to neutral (BH_2_)_3_(NMe_2_)_3_, which exhibits a chair conformation, the cationic portion of **7** has a distorted boat conformation, with a planar B1–B2–N3–N2 central part [Σ (angle of N_2_B_2_) = 360°] and N1 and B3 orientated on the same face. The boron atoms B1 and B2, involved in the three-center two-electron B–H–B unit, exhibit shorter B–N bond lengths [between 1.544(3) and 1.546(3) Å) than B3 (1.617(3) Å], consistent with B1 and B2 being more electron-deficient boron centers. This is also indicated by the N–B bonds in neutral **G** being considerably longer [1.614(6) Å]. The B1···B2 distance at 1.912(4) in **7** is also shorter than that in **G** [1.971(7) Å]; this difference is not due to the peri substitution in **G** as the B···B separation in (BBN)_2_(μ-H)(μ-NMe_2_) is 1.975(4) Å. However, 1.912(4) Å is still longer than a B–B single bond, and we attribute this short B···B separation to the contracted N1–B bonds involving the bridging NMe_2_ that are short as a result of the electron deficiency at B1 and B2 afforded by the cationic charge.

### 2.3 Reactivity Studies

With a series of bis-borane borocation equivalents isolated, we investigated briefly their reactivity toward simple bases. The significant steric crowding in **4** (indicated by the less common O-bound mode of NTf_2_ coordination) was confirmed by the fact that **4** does not bind bulky Lewis bases such as P^*t*^Bu_3_, or even PPh_3_, in contrast to other reactive (but less hindered) borocations.^14^ However, small nucleophilic bases do react with these bis-borane electrophiles, but they lead to cleavage of the B_2_N_2_ core. For example, the addition of 4-DMAP to **4** led to the precipitation of a solid that was confirmed by X-ray crystallographic studies to be the mono-boron species [(4-DMAP)_2_BH_2_][NTf_2_] (see Figure S82).

**Scheme 2:**
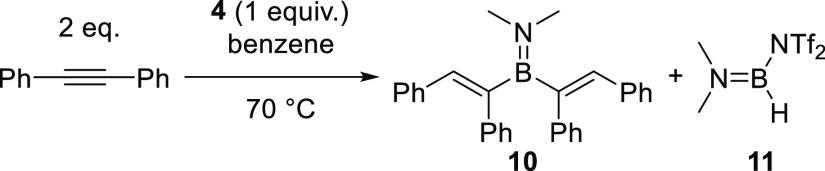
Hydroboration of Diphenylacetylene with **4** Internal conversion (vs an internal standard) ≈ 95% (after 4.5 days).

Moving to π nucleophiles, reactions between **3** and naphthalene, N–H-indole, N–Me-indole, or *N*-methylaniline gave no evidence for C–H borylation at ambient and raised temperatures, although indole hydroboration was observed. Similarly, no C–H borylation was observed for reactions between **4** and N–H-indole, N–Me-indole, or *N*-methylaniline (with or without 2,6-di-*tert*-butyl-4-methylpyridine as an exogenous base). As reduction of indoles to indolines is a known reaction achievable with L-BH_3_,^29^ these reactions were not investigated further. We turned our attention next to alkene and alkyne functionalization. When stilbene was combined with **3**, this gave no reaction. Hydroboration of stilbene by **4** was observed by ^1^H NMR spectroscopy; however, the slow rate of the reaction coupled with the formation of multiple species meant we did not pursue this reaction. Terminal alkynes treated with **4** reacted in a faster manner but led to the complex intractable mixtures; however, the reactivity with internal alkynes was cleaner. Reacting **4** with diphenylacetylene led to two new signals in the ^11^B NMR spectrum at δ_11B_ 40.0 (br, s) and 27.7 (d, ^1^*J*_BH_ = 180 Hz), while ^1^H NMR spectroscopy indicated the formation of a single hydroboration product (a vinylic C–H was observed at δ_1H_ 6.75 ppm). While the reaction is slow at 70 °C, higher temperatures cannot be used due to the limited stability of **4** in solution at raised temperatures. The δ_11B_ = 40 product was also observed using **3**, but this reaction was extremely slow (≈5% internal conversion vs an internal standard, after 5 days heating), consistent with the stronger coordination of iodide to boron relative to triflimide. Therefore, only reactivity with NTf_2_ derivatives is discussed from hereon. The broad signal at δ_11B_ = 40.0 is consistent with the formation of an R_2_B=NMe_2_ species,^30^ with R in this case presumably vinyl groups formed from the hydroboration of the alkyne to form **10** (Scheme 2). Further NMR experiments allowed us to confirm the formation of the divinylaminoborane **10** (see Figures S56–S60). No change in the reaction outcome was observed on repeating in the presence of exogenous base (in an attempt to effect intramolecular aryl C–H borylation after the initial hydroboration).

**Figure 7:**
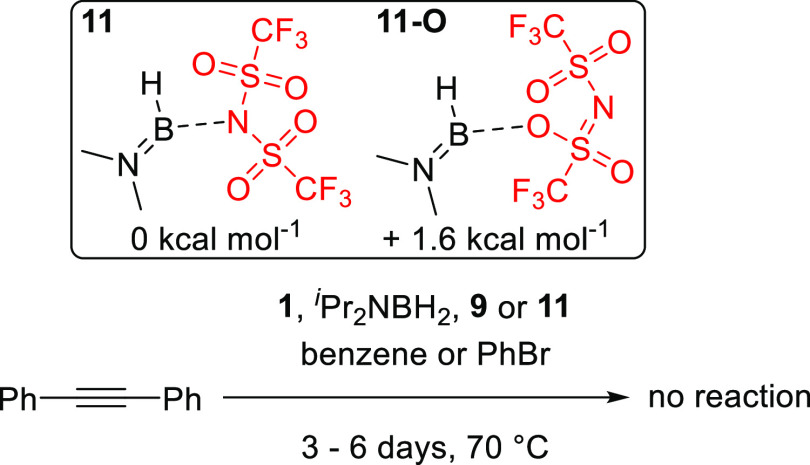
Inset, relative stability of **11** and **11-O**. Bottom, absence of reactivity from **1**, ^*i*^Pr_2_NBH_2_, and **9** toward diphenyl-acetylene.

Notably, the second observed signal at δ_11B_ = 27.7 while close to that reported for (Me_2_N)_2_BH (δ_11B_ 26–29, ^1^*J*_BH_ = 135–139 Hz),^31^ exhibited a ^1^*J*_BH_ = 180 Hz. This is more consistent with the formation of **11** (Scheme 2), which is closely comparable in spectroscopic data to **9**. The B_2_N_2_ core of **4** has split during formation of **10** and **11**, with **10** the product derived from the formal hydroboration of two equivalents of diphenylacetylene with Me_2_N=BH_2_, while **11** is then the expected byproduct to maintain mass balance. Stoichiometry studies confirmed the full consumption of all diphenylacetylene, and **4** occurs only at a ratio of 2:1. Crystallization of a reaction mixture post hydroboration enabled isolation (in a very small quantity) and structural characterization of the dimer of **11**, compound **6**. As discussed above **6** contains two O-bound NTf_2_, whereas in the monomeric derivatives **11** and **9**, NTf_2_ is N-bound (by ^19^F NMR spectroscopy and by crystallographic analysis for **9**). A sufficient quantity of pure **6** for NMR analysis was not obtainable by fractional crystallization, precluding full analysis. Nevertheless, the observation of O-bound NTf_2_ in the solid-state structure of **6** is consistent with the NMR data for **4** and calculations (the O-bound isomer of **4** is more stable than the N-bound by 11.5 kcal mol^–1^), in contrast calculations on the isomers of **11** (**11-O** and **11**, respectively) show that the N-bound NTf_2_ isomer is more stable by 1.6 kcal mol^–1^ than **11-O** (Figure 7, top inset), consistent with a less sterically encumbered boron center in **11** (relative to **4**).

The mechanism for the hydroboration of diphenylacetylene starting from **4** at raised temperature could proceed via bis-borane **4**, or via mono-boron species derived from the dissociation of **4** at raised temperatures. No significant hydroboration of diphenylacetylene was observed when it was treated with **1** or the mono-boron species ^*i*^Pr_2_NBH_2_ indicating the need for an electrophilic borane (Figure 7, bottom—see Figures S68–S75). More notably, ^*i*^Pr_2_NBH(NTf_2_), **9**, also did not affect hydroboration (<5% after 4 days at 70 °C). This indicates the necessity of an electrophilic bis-borane (as in **4**) for hydroboration to occur in these aminoborane systems. This is supported by the observation of **11** at the end of the reactions starting from **4** (even when using excess diphenylacetylene), indicating that **11** also does not hydroborate diphenylacetylene under these conditions. Therefore, the hydroboration of the first equivalent of diphenylacetylene occurs via the bis-borane **4** and not via mono-borane species from the dissociation of **4**. No intermediates between **4** and **10** are observed by NMR spectroscopy; thus, the subsequent steps occur rapidly relative to the first step of the reaction. Decreasing the temperature and the polarity of the solvent slowed the reaction rate; however, the reaction was cleanest in benzene (relative to reactions in haloarenes), therefore only reactions in benzene are discussed.

The disparity in reactivity towards diphenylacetylene of **4** versus **9/11** is notable as it indicates that a four coordinate at boron B-NTf_2_ species (in **4**) is more reactive in hydroboration than a three coordinate at boron B-NTf_2_ species (in **9/11**). However, it should be noted that in the mono-boron R_2_N=BH(NTf_2_) compounds there is significant B=NR_2_ multiple bond character; thus, the reaction of **4** and **9/11** with alkynes are all expected to proceed via displacement of NTf_2_ (by an S_N_1 or an S_N_2 at boron mechanism). Significant B=N character precluding hydroboration is supported by ^*i*^Pr_2_N=BH_2_, not effecting alkyne hydroboration under these conditions. If the hydroboration reaction with **4** or **9/11** proceeds via an S_N_1-type mechanism, then this would require dissociation of NTf_2_ from boron and formation of borenium (e.g., **[D]**^**+**^ from **4**) or borinium (e.g., [R_2_N=B–H]^+^ from **9/11**) cations. Borenium cations are much more energetically accessible than borinium cations, and we have recently shown that [R_2_N=BY]^+^ borinium cations are extremely high in energy and not feasible intermediates in C–H borylation.^32^ If an S_N_2-type mechanism is operative, the absence of any diphenylacetylene hydroboration starting from **9/11** can be attributed to the stronger binding of NTf_2_ to boron via nitrogen relative to O-bound NTf_2_ (as found in bis-borane **4**), leading to a higher reaction barrier.

**Scheme 3:**
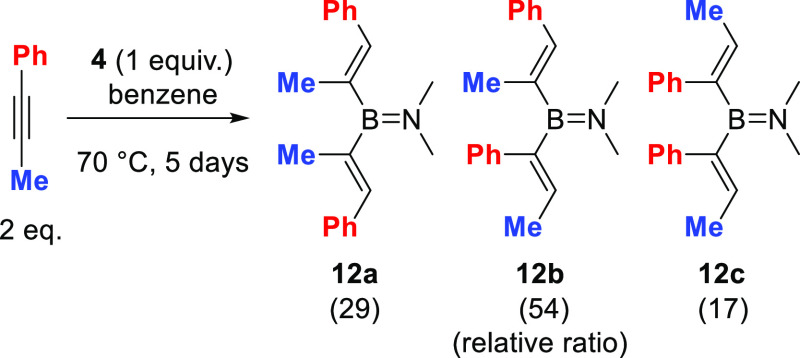
Synthesis of **12a–c** Internal conversion (vs an internal standard) ≈ 81% (after 5 days).

With a better understanding of the reaction, the regioselectivity in the hydroboration of an unsymmetric alkyne was probed. Hydroboration of 1-phenyl-1-propyne afforded a mixture of isomers, **12a**, **12b**, and **12c** (Scheme 3), in a ratio of 29:54:17 (see Figure S63) in a conversion of 81%. The Markovnikov/anti-Markovnikov regioselectivity therefore is low using **4** and is comparable to that previously reported for the hydroboration of the same substrate with (2,6-lutidine)BH_2_I.^18^ Attempts to alter the regioselectivity by varying the reaction conditions did not lead to any significant improvement. Furthermore, the use of **5** also led to a mixture of hydroboration isomers but the complexity of the *in situ*^1^H NMR spectra (on switching NMe_2_ for pyrrolidine) precluded determination of the exact ratios in this case. It should be noted that while **10** and **12** proved unstable to silica gel or distillation, fractional crystallization from pentane at low temperature afforded sufficiently pure material to enable the full characterization (see Figures S56–60 and S64–66).

**Scheme 4:**
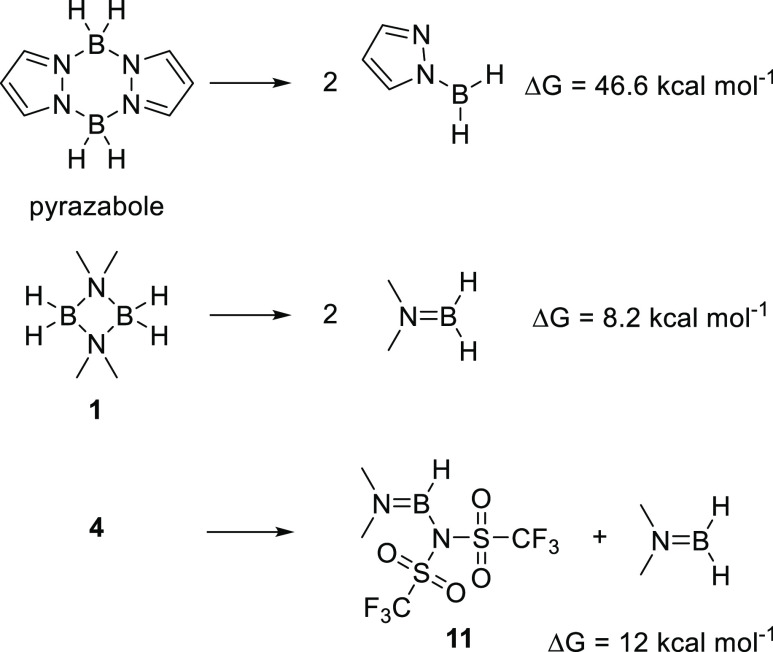
Comparison of the Energy Change during Dissociation of Pyrazabole, **1**, and **4** into Their Respective Mono-Boron Species

Finally, we were interested in understanding the greater propensity of these dimeric aminoborane derivatives to cleave to form mono-boron species compared to pyrazabole derivatives as the dissociation of **3** and **4** into mono-boron species is undesirable for their use in borylation-directed borylation.^9^ It was found that the dissociation was much more endergonic in the case of pyrazabole than that for [Me_2_NBH_2_]_2_, **1** (Scheme 4). This is in accordance with the lower stability observed in reactivity studies with the bis-borane aminoborane systems (e.g., **3** and **4**) relative to the pyrazaboles. We attribute this in part to ring strain in dimeric aminoboranes and the greater π donor ability of a NMe_2_ group relative to a pyrazole unit (where the N lone pair is part of the aromatic system) that helps stabilize the BH_2_ center in the monomeric form.

It should also be noted that substitution of H for NTf_2_ only alters the energy involved in dissociation of the bis-borane by a small amount (δΔ*G* = 3.8 kcal mol^–1^, Scheme 4 bottom); thus, it can also can be expected to split into mono-boron species to some degree, particularly on heating. Notably, heating **4** for 3 days in benzene at 100 °C (in a sealed tube) led to the observation (by ^11^B NMR spectroscopy) of small quantities of **11**. The fact that **11** is observed at room temperature from this reaction, that is, it is not consumed to reform **4** on cooling, was surprising based on the DFT calculations. This observation is attributed tentatively to a significant kinetic barrier associated with the linkage isomerism that has to occur to convert from N-bound NTf_2_ in **11** to form O-bound NTf_2_ in **4**.

## 3 Conclusions

In conclusion, a series of dimeric aminoborane-derived electrophiles were synthesized using readily accessible starting material. The optimal coordinating ability of the anion X to boron in H_2_B(μ-NR_2_)_2_BHX electrophiles is crucial, with iodide proving too coordinating (inhibiting reactivity with π nucleophiles), while [B(C_6_F_5_)_4_]^−^ is insufficiently coordinating which leads to further reactivity to form a B_3_N_3_-based cation. Triflimide (NTf_2_) proved to be optimal, enabling bis-borane electrophiles with B_2_N_2_ cores to be accessed that do react relatively cleanly with certain π nucleophiles. Thus, hydroboration of internal alkynes was achieved using H_2_B(μ-NR_2_)_2_BH(NTf_2_) with the B_2_N_2_ core maintained during the first hydroboration but subsequently splitting to ultimately produce R_2_N=B(vinyl)_2_. Notably, mono-boron analogues, for example, R_2_NBH(NTf_2_) exist with N-bound NTf_2_ in contrast to the bis-boranes and are much less reactive in alkyne hydroboration. This represents an unusual case where the four coordinate at boron species, H_2_B(μ-NR_2_)_2_BH(NTf_2_), is more reactive than a three coordinate at boron analogue R_2_NBH(NTf_2_). Finally, the stability of dimeric bis-boranes with respect to dissociation into monomers needs to be carefully considered for use in borylation-directed borylation, with the B_2_N_2_ core in dimeric aminoboranes too weakly bound for that particular application.

## 4 Experimental Section

### 4.1 General Materials and Methods

All reactions were performed under inert conditions using standard Schlenk techniques or in an MBraun Unilab glovebox (<0.1 ppm H_2_O/O_2_). Unless otherwise stated, solvents were degassed with nitrogen, dried over activated aluminum oxide (Solvent Purification System: Inert PureSolv MD5 SPS), and stored over 3 Å molecular sieves in ampules equipped with Young’s valves. Chlorobenzene, 1,2-difluorobenzene, and 1,2-dichlorobenzene were dried over calcium hydride, distilled, and stored over 3 Å molecular sieves. Deuterated solvents [CDCl_3_, C_6_D_6_, and C_6_D_5_Br (99.6% D, Sigma-Aldrich)] were dried and stored over 3 Å molecular sieves. All chemicals were, unless stated otherwise, purchased from commercial sources and used as received. BH_3_·SMe_2_ was transferred to an ampule fitted with Young’s valve prior to use. [Ph_3_C][B(C_6_F_5_)_4_] and lithium 2-^*t*^Bu-dihydropyridine were synthesized following the literature procedure.^33,34^ NMR spectra [^1^H, ^1^H{^11^B}, ^11^B, ^11^B{^1^H}, ^13^C{^1^H}, and ^19^F] were recorded on Bruker Avance III 400 MHz, Bruker Avance III 500 MHz, Bruker Avance III 600 MHz, or Bruker PRO 500 MHz spectrometers. Chemical shifts (δ) are quoted in parts per million (ppm), and coupling constants (*J*) are given in hertz (Hz) to the nearest 0.5 Hz and as positive values regardless of their real individual signs. ^1^H and ^13^C shifts are referenced to the appropriate residual solvent peak, while ^11^B and ^19^F shifts are referenced relative to external BF_3_·Et_2_O and C_6_F_6_, respectively. Abbreviations used are s (singlet), d (doublet), t (triplet), q (quartet), sep (septet), m (multiplet), and br (broad). Background signals in ^11^B NMR spectra arise to a significant degree from glass components of the probes used in our spectrometers. Unless otherwise stated, all NMR spectra were recorded at 20 °C. Mass spectrometry was performed at the Scottish Instrumentation and Resource Centre for Advanced Mass Spectrometry (SIRCAMS) at the University of Edinburgh using electron impact (EI) or electrospray ionization (ESI) techniques. CHN elemental analyses were carried out by Elemental Microanalysis Ltd. FTIR spectra were recorded on Shimadzu IRAffinity-1S FTIR.

### 4.2 Synthesis of Boron Electrophiles and Precursors

#### 4.2.1 Preparation of [Me_2_NBH_2_]_2_, **1**

Borane dimethylamine complex (3.00 g, 51.00 mmol, 1.00 equiv) and lithium 2-^*t*^Bu-dihydropyridine (0.18 g, 1.30 mmol, 2.5 mol %) were added to a Schlenk flask. Using a bend adapter tube, the flask was connected to a second Schlenk flask, immersed in an ice bath. The mixture was heated overnight at 100 °C with the collection Schlenk flask being left opened to the nitrogen atmosphere. The product started subliming during overnight heating. More product **1** was isolated by further sublimation (heat-gun under N_2_) as colorless crystals in 55% yield (1.58 g, 13.92 mmol). Analytical data were in accordance with literature values.^35^^1^H NMR (500 MHz, CDCl_3_, 300 K): δ 2.64 (1:1:1:1, q, ^1^*J*_HB_ = 112 Hz, 4H, B*H*), 2.45 (s, 12H, N(C*H*_3_)_2_); ^11^B NMR (160 MHz, CDCl_3_, 300 K): δ 5.2 (t, ^1^*J*_BH_ = 112 Hz, *B*H); ^11^B {^1^H} NMR (160 MHz, CDCl_3_, 300 K): δ 5.2 (s, *B*H); ^13^C {^1^H} NMR (126 MHz, CDCl_3_, 300 K): δ 51.93 (s, N(*C*H_3_)_2_).

#### 4.2.2 Preparation of [Pyrrolidine-BH_2_]_2_, **2**

Neat BH_3_·Me_2_S (2.63 mL, 27.70 mmol, 1.00 equiv) was slowly added to a solution of pyrrolidine (2.00 g, 27.70 mmol, 1.00 equiv) in pentane (30 mL) at room temperature. The solution was stirred for 1 h. Volatiles were removed *in vacuo* affording the pyrrolidine·BH_3_ adduct as a white solid. Lithium 2-^*t*^Bu-dihydropyridine (0.10 g, 0.69 mmol, 2.5 mol %) was added to the crude pyrrolidine·BH_3_ adduct, and the mixture was heated overnight at 100 °C in an unpressurized system. Product **2** was isolated by distillation (100 °C, 10^–3^ to 10^–2^ mbar) as a colorless waxy solid in 80% yield (1.84 g, 11.09 mmol). Analytical data were in accordance with literature values.^36^^1^H NMR (500 MHz, CDCl_3_, 300 K): δ 2.86 (br, t, 8H, NC*H*_2_CH_2_), 2.61 (1:1:1:1, q, ^1^*J*_HB_ = 112 Hz, 4H, B*H*), 1.76–1.66 (m, 8H, NCH_2_C*H*_2_); ^11^B NMR (160 MHz, CDCl_3_, 300 K): δ 3.0 (t, ^1^*J*_BH_ = 112 Hz, *B*H); ^11^B {^1^H} NMR (160 MHz, CDCl_3_, 300 K): δ 3.0 (s, *B*H); ^13^C {^1^H} NMR (126 MHz, CDCl_3_, 300 K): δ 60.08 (s, N*C*H_2_CH_2_), 23.62 (s, NCH_2_*C*H_2_).

#### 4.2.3 Preparation of H_2_B(μ-Me_2_N)_2_BH(I), **3**

Iodine (0.54 g, 2.11 mmol, 0.48 equiv) was dissolved in benzene (10 mL) and slowly added to a solution of [Me_2_NBH_2_]_2_ (0.50 g, 4.39 mmol, 1.00 equiv) in benzene (10 mL) at room temperature. The resulting solution was stirred at room temperature for 30 min. The volatiles were removed under vacuum with care, to avoid the solid product subliming during the process. The product was extracted with pentane (5 mL). After vacuuming the volatiles, the product **3** was isolated by sublimation under vacuum (heat-gun, 10^–3^ to 10^–2^ mbar) as a colorless crystalline solid in 34% yield (0.35 g, 1.47 mmol). ^1^H NMR (500 MHz, C_6_D_6_, 300 K): δ 4.37 (1:1:1:1, q, ^1^*J*_HB_ = 142 Hz, 1H, B*H*I), 2.88 (1:1:1:1, q, ^1^*J*_HB_ = 119 Hz, 1H, B*H*), 2.69 (1:1:1:1, q, ^1^*J*_HB_ = 115 Hz, 1H, B*H*), 2.22 (s, 6H, (NC*H*_3_)_2_), 2.00 (s, 6H, (NC*H*_3_)_2_); ^11^B NMR (160 MHz, C_6_D_6_, 300 K): δ 3.9 (t, ^1^*J*_BH_ = 118 Hz, *B*H_2_), 0.4 (d, ^1^*J*_BH_ = 142 Hz, *B*HI); ^11^B {^1^H} NMR (160 MHz, C_6_D_6_, 300 K): δ 3.9 (s, *B*H_2_), 0.4 (s, *B*HI); ^13^C {^1^H} NMR (126 MHz, C_6_D_6_, 300 K): δ 51.24 (s, (N*C*H_3_)_2_), 49.86 (s, (N*C*H_3_)_2_) (see Figure S1). Elemental analysis: calculated for C_4_H_15_B_2_N_2_I: C 20.04%, H 6.31%, N 11.69%; observed: C 20.67%, H 6.46%, N 11.53%. IR: (ν_max_ (neat)/cm^–1^) 2499 (B–H), 2432 (B–H), 2358 (B–H).

#### 4.2.4 Preparation of H_2_B(μ-Me_2_N)_2_BH(OSOCF_3_NTf), **4**

HNTf_2_ (1.18 g, 4.18 mmol, 1.00 equiv) was dissolved in benzene (10 mL) and slowly added to a solution of [Me_2_NBH_2_]_2_ (0.50 g, 4.39 mmol, 1.05 equiv) in benzene (10 mL) at room temperature. The solution was stirred at room temperature for 24 h. The volatiles were removed under vacuum. The product was extracted with pentane (10 mL). Drying under vacuum for 1 h afforded the product **4** as a colorless oil in 83% yield (1.37 g, 3.48 mmol). ^1^H NMR (500 MHz, C_6_D_6_, 300 K): δ 3.14 (br, q, ^1^*J*_HB_ = 138 Hz, 1H, B(*H*)OS(O)(CF_3_)NTf), 2.33 (br, q, ^1^*J*_HB_ = 123 Hz, 2H, B*H*_2_), 1.96 (s, 3H, C*H*_3_), 1.88 (s, 3H, C*H*_3_), 1.87 (s, 3H, C*H*_3_), 1.79 (s, 3H, C*H*_3_); ^1^H {^11^B} NMR (500 MHz, C_6_D_6_, 300 K): δ 3.14 (br, s, 1H, B(*H*)OS(O)(CF_3_)NTf), 2.33 (br, s, 2H, B*H*_2_), 1.97 (s, 3H, C*H*_3_), 1.88 (s, 3H, C*H*_3_), 1.87 (s, 3H, C*H*_3_), 1.80 (s, 3H, C*H*_3_); ^11^B NMR (160 MHz, C_6_D_6_, 300 K): δ 4.9 (d, ^1^*J*_BH_ = 142 Hz, *B*(H)OS(O)(CF_3_)NTf), 3.3 (t, ^1^*J*_BH_ = 119 Hz, *B*H_2_); ^11^B {^1^H} NMR (160 MHz, C_6_D_6_, 300 K): δ 4.9 (s, *B*(H)OS(O)(CF_3_)NTf), 3.3 (s, *B*H_2_); ^13^C {^1^H} NMR (126 MHz, C_6_D_6_, 300 K): δ 119.99 (q, ^1^*J*_CF_ = 320 Hz, *C*F_3_), 119.43 (q, ^1^*J*_CF_ = 321 Hz, *C*F_3_), 49.40 (s, *C*H_3_), 49.32 (s, *C*H_3_), 44.48 (s, *C*H_3_), 44.43 (s, *C*H_3_); ^19^F NMR (471 MHz, C_6_D_6_, 300 K): δ −75.7 (s, 3F, C*F*_3_), −78.5 (s, 3F, C*F*_3_). Elemental analysis: calculated for C_6_H_15_B_2_F_6_N_3_O_4_S_2_: C 18.34%, H 3.85%, N 10.69%; observed: C 18.40%, H 3.62%, N 10.50%. IR: (ν_max_ (neat)/cm^–1^) 2515 (B–H), 2474 (B–H), 2372 (B–H).

#### 4.2.5 Preparation of H_2_B(μ-pyrrolidine)_2_BH(OSOCF_3_NTf), **5**

HNTf_2_ (1.21 g, 4.30 mmol, 1.00 equiv) was dissolved in benzene (10 mL) and slowly added to a solution of [pyrrolidine-BH_2_]_2_ (0.75 g, 4.52 mmol, 1.05 equiv) in benzene (10 mL) at room temperature. The solution was stirred at room temperature for 20 h. The volatiles were removed under vacuum. The product was extracted with pentane (10 mL). Drying under vacuum overnight at 40 °C afforded the product **5** as a colorless oil in 65% yield (1.24 g, 2.79 mmol). ^1^H NMR (500 MHz, C_6_D_6_, 300 K): δ 4.00–2.90 (br, m, 1H, B(*H*)OS(O)(CF_3_)NTf), 2.88–1.96 (m, 10H, NC*H*_2_CH_2_ and B*H*_2_), 1.35–1.11 (m, 8H, NCH_2_C*H*_2_); ^1^H {^11^B} NMR (500 MHz, C_6_D_6_, 300 K): δ 3.43 (br, s, 1H, B(*H*)OS(O)(CF_3_)NTf), 2.88–1.96 (m, 10H, NC*H*_2_CH_2_ and B*H*_2_), 1.35–1.11 (m, 8H, NCH_2_C*H*_2_); ^11^B NMR (160 MHz, C_6_D_6_, 300 K): δ 5.4–1.1 (m, *B*(H)OS(O)(CF_3_)NTf, *B*H_2_); ^11^B {^1^H} NMR (160 MHz, C_6_D_6_, 300 K): δ 3.5 (s, *B*(H)OS(O)(CF_3_)NTf), 2.8 (s, *B*H_2_); ^13^C {^1^H} NMR (126 MHz, C_6_D_6_, 300 K): δ 120.05 (q, ^1^*J*_CF_ = 320 Hz, *C*F_3_), 119.49 (q, ^1^*J*_CF_ = 322 Hz, *C*F_3_), 58.62 (s, N*C*H_2_CH_2_), 58.55 (s, N*C*H_2_CH_2_), 53.85 (s, N*C*H_2_CH_2_), 22.89 (s, NCH_2_*C*H_2_), 22.88 (s, NCH_2_*C*H_2_), 22.79 (s, NCH_2_*C*H_2_), 22.78 (s, NCH_2_*C*H_2_); ^19^F NMR (471 MHz, C_6_D_6_, 300 K): δ −75.7 (s, 3F, C*F*_3_), −78.4 (s, 3F, C*F*_3_). Elemental analysis: calculated for C_10_H_19_B_2_F_6_N_3_O_4_S_2_: C 26.99%, H 4.30%, N 9.44%; observed: C 26.94%, H 4.18%, N 9.39%. IR: (ν_max_ (neat)/cm^–1^) 2499 (B–H), 2451 (B–H), 2374 (B–H).

#### 4.2.6 Preparation of [(Me_2_N)_3_B_3_H_5_][B(C_6_F_5_)_4_], **7**

(Me_2_NBH_2_)_2_ (0.03 g, 0.22 mmol, 3.00 equiv) and [Ph_3_C][B(C_6_F_5_)_4_] (0.14 g, 0.15 mmol, 2 equiv) were dissolved in PhCl (3 mL) and heated to 60 °C until the solution turned colorless (30 min). The solution was carefully layered with hexane (5 mL). After 21 days, the formed colorless crystals were washed with hexane (2 × 2 mL) and dried *in vacuo*. The product **7** was isolated as colorless crystals in 70% yield (0.09 g, 0.10 mmol). ^1^H NMR^a,b^ (500 MHz, C_6_H_4_F_2_, 300 K): δ 3.45–2.14 (br, m, 2H, *H*B(H)B*H*), 3.45–1.74 (br, m, 18H, N(C*H*_3_)_2_), 2.83–1.74 (br, m, 2H, B*H*_2_), 1.72–1.10 (br, m, 1H, HB(*H*)BH); ^1^H NMR^a^ (500 MHz, C_6_H_4_F_2_, 278 K): δ 3.45–2.14 (br, m, 2H, *H*B(H)B*H*), 3.13 (s, 3H, N(C*H*_3_)), 2.83–1.74 (br, m, 2H, B*H*_2_), 2.55 (s, 6H, N(C*H*_3_)), 2.47 (s, 6H, N(C*H*_3_)), 2.36 (s, 3H, N(C*H*_3_)), 1.72–1.10 (br, m, 1H, HB(*H*)BH); ^1^H {^11^B} NMR^a,b^ (500 MHz, C_6_H_4_F_2_, 300 K): δ 3.45–2.14 (br, m, 2H, *H*B(H)B*H*), 3.45–1.74 (br, m, 18H, N(C*H*_3_)_2_), 2.83–1.74 (br, m, 2H, B*H*_2_), 1.52 (br, s, 1H, HB(*H*)BH); ^11^B NMR^a^ (160 MHz, C_6_H_4_F_2_, 300 K): δ 3.4 (t, ^1^*J*_BH_ = 120 Hz, *B*H_2_), −6.1 (br, d, ^1^*J*_BH_ = 164 Hz, H*B*(H)*B*H), −16.2 (s, *B*(C_6_F_5_)_4_); ^11^B {^1^H} NMR^a^ (160 MHz, C_6_H_4_F_2_, 300 K): δ 3.4 (s, *B*H_2_), −6.1 (s, H*B*(H)*B*H), −16.2 (s, *B*(C_6_F_5_)_4_); ^13^C {^1^H} NMR^a^ (126 MHz, C_6_H_4_F_2_, 300 K): δ 148.8 (br, m, *C*_*ortho*_F), 140.6 (br, t, *C*_*para*_F), 138.6 (br, m, *C*_*meta*_F), 136.7 (br, m, *C*_*ipso*_F), 54.3 (br, s, N(*C*H_3_)), 49.8 (br, s, N(*C*H_3_)), 46.4 (br, s, N(*C*H_3_)), 40.1 (br, s, N(*C*H_3_)); ^19^F NMR^a^ (471 MHz, C_6_H_4_F_2_, 300 K): δ −132.5 (br, s, C*F*_*ortho*_), −163.9 (t, ^3^*J*_FF_ = 20 Hz, C*F*_*para*_), −167.7 (br, t, ^3^*J*_FF_ = 17 Hz, C*F*_*meta*_).^a^ Due to solubility and stability issues, NMR data of **7** were recorded in 1,2-difluorobenzene (C_6_H_4_F_2_). Reference NMR experiments using SiMe_4_ were carried out to determine the ^1^H and ^13^C shifts of 1,2-difluorobenzene. Hydrogen atoms located on boron centers [B*H*_2_, *H*B(H)B*H*, and HB(*H*)BH] were identified by the 2D ^11^B–^1^H HMQC experiment. Mass spectrum: HRMS (ESI+) *m*/*z*: calcd for C_6_H_23_B_3_N_3_^+^: 170.21657; found: 170.21576. IR: (ν_max_ (neat)/cm^–1^) 2538 (B–H), 2465 (B–H), 2403 (B–H).

#### 4.2.7 Preparation of **10**

A solution of H_2_B(μ-Me_2_N)_2_BH(OSOCF_3_NTf), **4** (0.22 g, 0.56 mmol, 1.00 equiv) in benzene (1 mL) was added to a solution of diphenylacetylene (0.20 g, 1.12 mmol, 2.00 equiv) in benzene (1 mL). The resulting solution was heated at 70 °C for 1 week. While heating, the solution turned slowly from colorless to dark orange. The volatiles were removed under vacuum, affording an oil. The oil was extracted with pentane (2 mL), giving a clear orange solution. The solution was cooled to −35 °C and filtered at this temperature. Removal of volatiles *in vacuo* afforded the product **10** as an orange oil (0.11 g), contaminated with remaining trace of −NTf_2_ side-products. ^1^H NMR (500 MHz, C_6_D_6_, 300 K): δ 7.23–7.19 (m, 4H), 7.15–7.12 (m, 4H), 7.12–7.08 (m, 4H), 7.03–6.99 (m, 2H), 6.99–6.94 (m, 4H), 6.92–6.88 (m, 2H), 6.75 (s, 2H), 2.68 (s, 6H); ^11^B NMR (160 MHz, C_6_D_6_, 300 K): δ 40.0 (br, s, *B*N(Me)_2_); ^13^C {^1^H} NMR (126 MHz, C_6_D_6_, 300 K): δ 147.43 (br), 142.95, 138.50, 134.72, 129.77, 129.03, 128.82, 128.27^a^, 126.90, 126.29, 40.99. Mass spectrum: HRMS (ESI+) *m*/*z*: calcd for C_30_H_28_BN: 413.23093; found: 413.23142.

## Supporting Information Available

NMR spectra (PDF)
Coordinates for calculated compounds (XYZ)

## Supplementary Material

om2c00393_si_001.pdf

om2c00393_si_002.xyz
