# Supplementary material for: Synthesis of Electrophiles Derived from Dimeric Aminoboranes and Assessing Their Utility in the Borylation of π Nucleophiles
Source: Organometallics. 2022 Sep 14;41(18):2638–47. doi: 10.1021/acs.organomet.2c00393 (PMC9516688; doi:10.1021/acs.organomet.2c00393)
Supplement: Supplementary file 1 — om2c00393_si_001.pdf [file om2c00393_si_001.pdf]

The synthesis of electrophiles derived from dimeric aminoboranes and assessing their utility in the borylation of π nucleophiles.

Clement. R. P. Millet, Jürgen Pahl, Emily Noone, Kang Yuan, Gary S. Nichol, Marina Uzelac, Michael J. Ingleson*.

mingleso@ed.ac.uk

School of Chemistry, University of Edinburgh, Edinburgh, EH9 3FJ.

Contents

1 General considerations ... 4
2 Synthesis of boron electrophiles and precursors ... 5
2.1 Preparation of $[Me_2NBH_2]_2$ 1 ... 5
2.2 Preparation of $[Pyrrolidine\text{-}BH_2]_2$ 2 ... 5
2.3 Preparation of $H_2B(\mu\text{-}Me_2N)_2BH(I)$ 3 ... 6
2.4 Preparation of $H_2B(\mu\text{-}Me_2N)_2BH(OSOCF_3NTf)$ 4 ... 7
2.5 Preparation of $H_2B(\mu\text{-pyrrolidine})_2BH(OSOCF_3NTf)$ 5 ... 8
2.6 Preparation of $[(Me_2N)_3B_3H_5][B(C_6F_5)_4]$ 7 ... 9
3 Monomeric borocations equivalents ... 10
3.1 Generation of the adduct 8 ... 10
3.1.1 Preparation 1 ... 10
3.1.2 Preparation 2 ... 10
3.2 Generation of the monomeric borocation $(^iPr)_2NBHNTf_2$ 9 ... 11
3.3 NMR Spectra of $[Me_2NBH_2]_2$ 1 ... 12
3.4 NMR Spectra of $[Pyrrolidine\text{-}BH_2]_2$ 2 ... 13
3.5 NMR Spectra of $H_2B(\mu\text{-}Me_2N)_2BH(I)$ 3 ... 15
3.6 NMR Spectra of $H_2B(\mu\text{-}Me_2N)_2BH(OSOCF_3NTf)$ 4 ... 17
3.7 NMR Spectra of $H_2B(\mu\text{-pyrrolidine})_2BH(OSOCF_3NTf)$ 5 ... 20
3.8 NMR Spectra of $[(Me_2N)_3B_3H_5][B(C_6F_5)_4]$ Cation 7 ... 23
3.9 Reference NMR Spectra of $SiMe_4$ in difluorobenzene ($C_6H_4F_2$) ... 27
3.10 FTIR Spectra of boron electrophiles ... 28
3.11 NMR spectra of 8 ... 30
3.12 NMR spectra of $(^iPr)_2NBHNTf_2$ 9 ... 33
4 Hydroboration of alkynes ... 36
4.1 Hydroboration of diphenylacetylene with 3 ... 36
4.1.1 Internal conversion to 10 ... 36
4.2 Hydroboration of diphenylacetylene with 4 ... 37
4.2.1 Internal conversion to 10 ... 37
4.2.2 Characterisation of 10 ... 39
4.2.3 NMR Spectra of 10 ... 40
4.3 Hydroboration of 1-phenyl-1-propyne with 4 ... 42
4.3.1 Internal conversion to 12a-c ... 42

4.3.2 Characterisation of isomers of 12a-c 44
4.3.3 NMR Spectra of 12a-c 45
5 Complementary studies 47
5.1 Attempt of synthesis of $[Me_2NBH(I)]_2$ 47
5.2 Control reactions 48
5.2.1 Hydroboration of diphenylacetylene with $[Me_2NBH_2]_2$ 1 48
5.2.2 Hydroboration of diphenylacetylene with $[Me_2NBH_2]_2$ 1 and catalytic amount of 4 49
5.2.3 Hydroboration of diphenylacetylene with $(^iPr)_2NBHNTf_2$ 9 51
5.2.4 Hydroboration of diphenylacetylene with $(^iPr)_2NBH_2$ 52
5.3 Solvent effect: Hydroboration of Diphenylacetylene with 4 in polar solvent 53
5.4 Temperature effect: Hydroboration of Diphenylacetylene with 4 in polar solvent at 40 °C 54
5.5 Study of the By-product from hydroboration reaction 55
5.5.1 In situ observation of by-product 11 55
5.5.2 In situ generation of 11 from 1 55
5.5.3 In situ generation of 11 from 4 56
5.5.4 Formation of 6 57
6 Crystallographic data 58
6.1 Crystal structure of 3 58
6.2 Crystal structure of 6 59
6.3 Crystal structure of 7 60
6.4 Crystal structure of 9 61
6.5 Crystal Structure of $[(4\text{-DMAP})_2BH_2][NTf_2]$ 62
7 Computational Details 64
8 References 65

1 General considerations

All reactions were performed under inert conditions using standard Schlenk techniques or in an *MBraun Unilab* glovebox (<0.1 ppm H_2O / O_2).

Unless otherwise stated, solvents were degassed with nitrogen, dried over activated aluminium oxide (Solvent Purification System: Inert PureSolv MD5 SPS) and stored over 3 Å molecular sieves in ampules equipped with J. Young's valves. Chlorobenzene, 1,2-difluorobenzene and 1,2-dichlorobenzene were dried over calcium hydride, distilled and stored over 3 Å molecular sieves. Deuterated solvents ($CDCl_3$, C_6D_6 and C_6D_5Br (99.6% D, Sigma Aldrich)) were dried and stored over 3 Å molecular sieves. All chemicals were, unless stated otherwise, purchased from commercial sources and used as received. $BH_3{\cdot}SMe_2$ was transferred to an ampule fitted with a J. Young's valve prior to use. $[Ph_3C][B(C_6F_5)_4]$ and lithium 2-^{t}Bu-dihydropyridine were synthesized following literature procedure.[S1,S2]

NMR spectra (^{1}H, ^{1}H{^{11}B}, ^{11}B, ^{11}B{^{1}H}, ^{13}C{^{1}H} and ^{19}F) were recorded on *Bruker Avance III 400 MHz, Bruker Avance III 500 MHz, Bruker Avance III 600 MHz* or *Bruker PRO 500 MHz* spectrometers. Chemical shifts (δ) are quoted in parts per million (ppm), coupling constants (*J*) are given in hertz (Hz) to the nearest 0.5 Hz, and as positive vales regardless of their real individual signs. ^{1}H and ^{13}C shifts are referenced to the appropriate residual solvent peak while ^{11}B and ^{19}F shifts are referenced relative to external $BF_3{\cdot}Et_2O$ and C_6F_6, respectively. Abbreviations used are s (singlet), d (doublet), t (triplet), q (quartet), sep (septet), m (multiplet), br (broad). Background signals in ^{11}B NMR spectra arise to a significant degree from glass components of the probes used in our spectrometers. Unless otherwise stated, all NMR spectra were recorded at 20 °C.

Mass spectrometry was performed by the *Scottish Instrumentation and Resource Centre for Advanced Mass Spectrometry* (SIRCAMS) at the University of Edinburgh using electron impact (EI) or electrospray ionisation (ESI) techniques.

CHN Elemental Analyses were carried out by *Elemental Microanalysis Ltd*.

FTIR spectra were recorded on *Shimadzu IRAffinity-1S FTIR*.

2 Synthesis of boron electrophiles and precursors

2.1 Preparation of $[Me_2NBH_2]_2$ 1

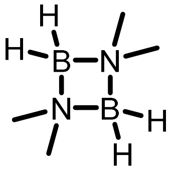

Borane dimethylamine complex (3.00 g, 51.00 mmol, 1.00 equiv.) and lithium 2-^{t}Bu-dihydropyridine (0.18 g, 1.30 mmol, 2.5 mol%) were added to a Schlenk flask. Using a bend adapter tube, the flask was connected to a second collection Schlenk flask, immersed in an ice bath. The mixture was heated overnight at 100 °C with the collection Schlenk flask being left opened to Nitrogen atmosphere. The product started subliming during overnight heating. More product **1** was isolated by further sublimation (heat-gun, N_2 Schlenk line pressure) as colourless crystals in 55% yield (1.58 g, 13.92 mmol). Analytical data were in accordance with literature values.[S3,S4]

^{1}H NMR (500 MHz, $CDCl_3$, 300 K) δ 2.64 (1:1:1:1, q, $^1J_{HB}$ = 112 Hz, 4H, B*H*), 2.45 (s, 12H, $N(CH_3)_2$).

^{11}B NMR (160 MHz, $CDCl_3$, 300 K) δ 5.2 (t, $^1J_{BH}$ = 112 Hz, *B*H).

^{11}B {^{1}H} NMR (160 MHz, $CDCl_3$, 300 K) δ 5.2 (s, *B*H).

^{13}C {^{1}H} NMR (126 MHz, $CDCl_3$, 300 K) δ 51.93 (s, $N(CH_3)_2$).

2.2 Preparation of [Pyrrolidine-BH_2]$_2$ 2

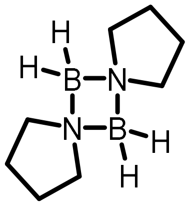

Neat $BH_3{\cdot}Me_2S$ (2.63 mL, 27.70 mmol, 1.00 equiv.) was slowly added to a solution of Pyrrolidine (2.00 g, 27.70 mmol, 1.00 equiv.) in pentane (30 mL) at room temperature. The solution was stirred for one hour. Volatiles were removed *in vacuo* affording the Pyrrolidine·BH_3 adduct as a white solid. Lithium 2-^{t}Bu-dihydropyridine (0.10 g, 0.69 mmol, 2.5 mol%) was added to the crude Pyrrolidine·BH_3 adduct and the mixture was heated overnight at 100 °C in an unpressurized system. Product **2** was isolated by

distillation (100 °C, 10^{-3} to 10^{-2} mbar) as a colourless waxy solid in 80% yield (1.84 g, 11.09 mmol). Analytical data were in accordance with literature values.[S5]

^{1}H NMR (500 MHz, $CDCl_3$, 300 K) δ 2.86 (br, t, 8H, NCH_2CH$_2$), 2.61 (1:1:1:1, q, $^{1}J_{HB}$ = 112 Hz, 4H, B*H*), 1.76-1.66 (m, 8H, NCH$_2$CH_2).

^{11}B NMR (160 MHz, $CDCl_3$, 300 K) δ 3.0 (t, $^{1}J_{BH}$ = 112 Hz, *B*H).

^{11}B {^{1}H} NMR (160 MHz, $CDCl_3$, 300 K) δ 3.0 (s, *B*H).

^{13}C {^{1}H} NMR (126 MHz, $CDCl_3$, 300 K) δ 60.08 (s, N*C*H$_2$CH$_2$), 23.62 (s, NCH$_2$*C*H$_2$).

2.3 Preparation of $H_2B(\mu\text{-}Me_2N)_2BH(I)$ 3

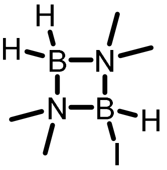

Iodine (0.54 g, 2.11 mmol, 0.48 equiv.) was dissolved in benzene (10 mL) and slowly added to a solution of $[Me_2NBH_2]_2$ (0.50 g, 4.39 mmol, 1.00 equiv.) in benzene (10 mL) at room temperature. The resulting solution was stirred at room temperature for 30 min. The volatiles were removed under vacuum, avoiding the solid product to sublime during the process. The product was extracted with pentane (5 mL). After vacuuming the volatiles, the product **3** was isolated by sublimation under vacuum (heat-gun, 10^{-3} to 10^{-2} mbar) as a colourless crystalline solid in 34% yield (0.35 g, 1.47 mmol).

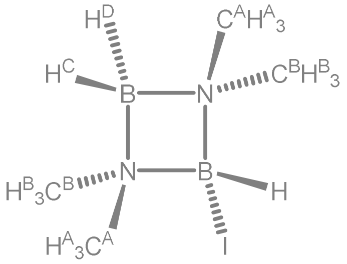

Figure S1: Scheme of **3**.

^{1}H NMR (500 MHz, C_6D_6, 300 K) δ 4.37 (1:1:1:1, q, $^{1}J_{HB}$ = 142 Hz, 1H, B*H*I), 2.88 (1:1:1:1, q, $^{1}J_{HB}$ = 119 Hz, 1H, BH^{C}*H^{D}*), 2.69 (1:1:1:1, q, $^{1}J_{HB}$ = 115 Hz, 1H, B*H^{C}*H^{D}), 2.22 (s, 6H, (NC^{B}*H$^{B}_{3}$*)$_2$), 2.00 (s, 6H, (NC^{A}*H$^{A}_{3}$*)$_2$).

^{11}B NMR (160 MHz, C_6D_6, 300 K) δ 3.9 (t, $^{1}J_{BH}$ = 118 Hz, *B*H^CH^D), 0.4 (d, $^{1}J_{BH}$ = 142 Hz, *B*HI).

^{11}B {^{1}H} NMR (160 MHz, C_6D_6, 300 K) δ 3.9 (s, *B*H^CH^D), 0.4 (s, *B*HI).

^{13}C {^{1}H} NMR (126 MHz, C_6D_6, 300 K) δ 51.24 (s, $(NC^BH^B{}_3)_2$), 49.86 (s, $(NC^AH^A{}_3)_2$).

Elemental Analysis: Calculated for $C_4H_{15}B_2N_2I$: C 20.04%, H 6.31%, N 11.69%; Observed: C 20.67%, H 6.46%, N 11.53%.

IR: (vmax (neat)/cm^{-1}) 2499 (B–H), 2432 (B–H), 2358 (B–H).

2.4 Preparation of $H_2B(\mu\text{-}Me_2N)_2BH(OSOCF_3NTf)$ 4

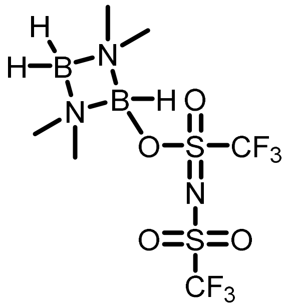

$HNTf_2$ (1.18 g, 4.18 mmol, 1.00 equiv.) was dissolved in benzene (10 mL) and slowly added to a solution of $[Me_2NBH_2]_2$ (0.50 g, 4.39 mmol, 1.05 equiv.) in benzene (10 mL) at room temperature. The solution was stirred at room temperature for 24 hours. The volatiles were removed under vacuum. The product was extracted with pentane (10 mL). Drying under vacuum for 1 hour afforded the product **4** as a colourless oil in 83% yield (1.37 g, 3.48 mmol).

^{1}H NMR (500 MHz, C_6D_6, 300 K) δ 3.14 (br, q, $^{1}J_{HB}$ = 138 Hz, 1H, B(*H*)OS(O)(CF_3)NTf), 2.33 (br, q, $^{1}J_{HB}$ = 123 Hz, 2H, BH_2), 1.96 (s, 3H, CH_3), 1.88 (s, 3H, CH_3), 1.87 (s, 3H, CH_3), 1.79 (s, 3H, CH_3).

^{1}H {^{11}B} NMR (500 MHz, C_6D_6, 300 K) δ 3.14 (br, s, 1H, B(*H*)OS(O)(CF_3)NTf), 2.33 (br, s, 2H, BH_2), 1.97 (s, 3H, CH_3), 1.88 (s, 3H, CH_3), 1.87 (s, 3H, CH_3), 1.80 (s, 3H, CH_3).

^{11}B NMR (160 MHz, C_6D_6, 300 K) δ 4.9 (d, $^{1}J_{BH}$ = 142 Hz, *B*(H)OS(O)(CF_3)NTf), 3.3 (t, $^{1}J_{BH}$ = 119 Hz, *B*H_2).

^{11}B {^{1}H} NMR (160 MHz, C_6D_6, 300 K) δ 4.9 (s, *B*(H)OS(O)(CF_3)NTf), 3.3 (s, *B*H_2).

^{13}C {^{1}H} NMR (126 MHz, C_6D_6, 300 K) δ 119.99 (q, $^{1}J_{CF}$ = 320 Hz, *C*F_3), 119.43 (q, $^{1}J_{CF}$ = 321 Hz, *C*F_3), 49.40 (s, *C*H_3), 49.32 (s, *C*H_3), 44.48 (s, *C*H_3), 44.43 (s, *C*H_3).

^{19}**F NMR** (471 MHz, C_6D_6, 300 K) δ –75.7 (s, 3F, C*F*$_3$), –78.5 (s, 3F, C*F*$_3$).

Elemental Analysis: Calculated for $C_6H_{15}B_2F_6N_3O_4S_2$: C 18.34%, H 3.85%, N 10.69%; Observed: C 18.40%, H 3.62%, N 10.50%.

IR: (vmax (neat)/cm^{-1}) 2515 (B–H), 2474 (B–H), 2372 (B–H).

2.5 Preparation of H₂B(μ-pyrrolidine)₂BH(OSOCF₃NTf) 5

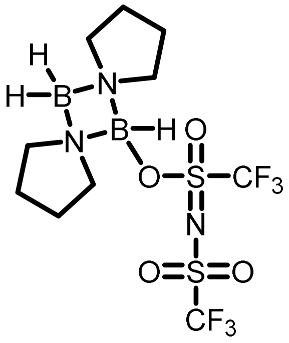

$HNTf_2$ (1.21 g, 4.30 mmol, 1.00 equiv.) was dissolved in benzene (10 mL) and slowly added to a solution of [Pyrrolidine-BH_2]$_2$ (0.75 g, 4.52 mmol, 1.05 equiv.) in benzene (10 mL) at room temperature. The solution was stirred at room temperature for 20 hours. The volatiles were removed under vacuum. The product was extracted with pentane (10 mL). Drying under vacuum overnight at 40 °C afforded the product **5** as a colourless oil in 65% yield (1.24 g, 2.79 mmol). Very slow decomposition of **5** was observed over months, even when kept under inert atmosphere.

^{1}**H NMR** (500 MHz, C_6D_6, 300 K) δ 4.00-2.90 (br, m, 1H, B(*H*)OS(O)(CF$_3$)NTf), 2.88-1.96 (m, 10H, NC*H*$_2$CH$_2$ and B*H*$_2$), 1.35-1.11 (m, 8H, NCH$_2$C*H*$_2$).

^{1}**H {^{11}B} NMR** (500 MHz, C_6D_6, 300 K) δ 3.43 (br, s, 1H, B(*H*)OS(O)(CF$_3$)NTf), 2.88-1.96 (m, 10H, NC*H*$_2$CH$_2$ and B*H*$_2$), 1.35-1.11 (m, 8H, NCH$_2$C*H*$_2$).

^{11}**B NMR** (160 MHz, C_6D_6, 300 K) δ 5.4-1.1 (m, *B*(H)OS(O)(CF$_3$)NTf), *B*H$_2$).

^{11}**B {^{1}H} NMR** (160 MHz, C_6D_6, 300 K) δ 3.5 (s, *B*(H)OS(O)(CF$_3$)NTf), 2.8 (s, *B*H$_2$).

^{13}**C {^{1}H} NMR** (126 MHz, C_6D_6, 300 K) δ 120.05 (q, $^{1}J_{CF}$ = 320 Hz, *C*F$_3$), 119.49 (q, $^{1}J_{CF}$ = 322 Hz, *C*F$_3$), 58.62 (s, N*C*H$_2$CH$_2$), 58.55 (s, N*C*H$_2$CH$_2$), 53.85 (s, N*C*H$_2$CH$_2$), 22.89 (s, NCH$_2$*C*H$_2$), 22.88 (s, NCH$_2$*C*H$_2$), 22.79 (s, NCH$_2$*C*H$_2$), 22.78 (s, NCH$_2$*C*H$_2$). Note the resonance at 53.85 is twice the intensity of the resonances at 58.62 and 58.55 thus we assume that two signals are coincident.

^{19}**F NMR** (471 MHz, C_6D_6, 300 K) δ –75.7 (s, 3F, C*F*$_3$), –78.4 (s, 3F, C*F*$_3$).

Elemental Analysis: Calculated for $C_{10}H_{19}B_2F_6N_3O_4S_2$: C 26.99%, H 4.30%, N 9.44%; Observed: C 26.94%, H 4.18%, N 9.39%.

IR: (vmax (neat)/cm^{-1}) 2499 (B–H), 2451 (B–H), 2374 (B–H).

2.6 Preparation of $[(Me_2N)_3B_3H_5][B(C_6F_5)_4]$ 7

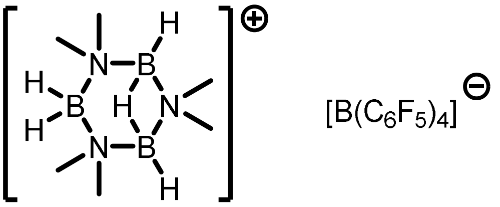

$(Me_2NBH_2)_2$ (0.03 g, 0.22 mmol, 3.00 equiv.) and $[Ph_3C][B(C_6F_5)_4]$ (0.14 g, 0.15 mmol, 2 equiv.) were dissolved in PhCl (3 mL) and heated to 60 °C until the solution turned colourless (30 min). The solution was carefully layered with hexane (5 mL). After 21 days the formed colourless crystals were washed with hexane (2 x 2mL) and dried *in vacuo.* The product **7** was isolated as colourless crystals in 70% yield (0.09 g, 0.10 mmol).

^{1}H NMR[a, b] (500 MHz, $C_6H_4F_2$, **300 K**) δ 3.45-2.14 (br, m, 2H, *H*B(H)B*H*), 3.45-1.74 (br, m, 18H, N(CH_3)$_2$), 2.83-1.74 (br, m, 2H, BH_2), 1.72-1.10 (br, m, 1H, HB(*H*)BH).

^{1}H NMR[a, b] (500 MHz, $C_6H_4F_2$, **278 K**) δ 3.45-2.14 (br, m, 2H, *H*B(H)B*H*), 3.13 (s, 3H, N(CH_3)), 2.83-1.74 (br, m, 2H, BH_2), 2.55 (s, 6H, N(CH_3)), 2.47 (s, 6H, N(CH_3)), 2.36 (s, 3H, N(CH_3)), 1.72-1.10 (br, m, 1H, HB(*H*)BH).

^{1}H {^{11}B} NMR[a, b] (500 MHz, $C_6H_4F_2$, **300 K**) δ 3.45-2.14 (br, m, 2H, *H*B(H)B*H*), 3.45-1.74 (br, m, 18H, N(CH_3)$_2$), 2.83-1.74 (br, m, 2H, BH_2), 1.52 (br, s, 1H, HB(*H*)BH).

^{11}B NMR[a] (160 MHz, $C_6H_4F_2$, 300 K) δ 3.4 (t, $^{1}J_{BH}$ = 120 Hz, *B*H$_2$), –6.1 (br, d, $^{1}J_{BH}$ = 164 Hz, H*B*(H)*B*H), –16.2 (s, *B*(C_6F_5)$_4$).

^{11}B {^{1}H} NMR[a] (160 MHz, $C_6H_4F_2$, 300 K) δ 3.4 (s, *B*H$_2$), –6.1 (s, H*B*(H)*B*H), –16.2 (s, *B*(C_6F_5)$_4$).

^{13}C {^{1}H} NMR[a] (126 MHz, $C_6H_4F_2$, 300 K) δ 148.8 (br, m, C_{ortho}F), 140.6 (br, t, C_{para}F), 138.6 (br, m, C_{meta}F), 136.7 (br, m, C_{ipso}F), 54.3 (br, s, N(*C*H$_3$)), 49.8 (br, s, N(*C*H$_3$)), 46.4 (br, s, N(*C*H$_3$)), 40.1 (br, s, N(*C*H$_3$)).

^{19}F NMR[a] (471 MHz, $C_6H_4F_2$, 300 K) δ –132.5 (br, s, CF_{ortho}), –163.9 (t, $^{3}J_{FF}$ = 20 Hz, CF_{para}), –167.7 (br, t, $^{3}J_{FF}$ = 17 Hz, CF_{meta}).

[a] NMR data of **7** were recorded in 1,2-difluorobenzene ($C_6H_4F_2$). Reference NMR experiments using $SiMe_4$ were carried out to determine the ^1H and ^{13}C shift of 1,2-difluorobenzene. ^{11}B and ^{19}F chemical shift of **7** are given from 1,2-difluorobenzene without reference NMR being run prior.

[b] Further NMR experiments were carried out for the ^1H NMR characterisation of **7**. ^1H NMR experiment at 278 K allowed assignment of the different N(C*H*$_3$) signals. Hydrogen atoms located on boron centres (B*H*$_2$, *H*B(H)B*H* and HB(*H*)BH) were identified by 2-D ^{11}B–^1H HMQC experiment.

Mass Spectrum: HRMS (ESI+) m/z calculated for $C_6H_{23}B_3N_3^+$: 170.21657; Found: 170.21576.

IR: (vmax (neat)/cm^{-1}) 2538 (B–H), 2465 (B–H), 2403 (B–H).

3 Monomeric borocations equivalents

3.1 Generation of the adduct 8

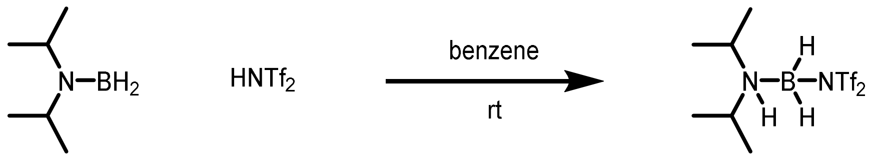

8

3.1.1 Preparation 1

(^{i}Pr)$_2$NBH$_2$ (10 mg, 0.09 mmol, 1.00 equiv.), HNTf$_2$ (25 mg, 0.09 mmol, 1.00 equiv.) and mesitylene (12 µL, 0.09 mmol, 1.00 equiv.) were dissolved in C_6D_6 (0.5 mL) and heated at 60 °C for 3 hours. NMR experiments confirmed the full conversion to a new product, which was assigned to be the adduct **8**.

3.1.2 Preparation 2

(^{i}Pr)$_2$NBH$_2$ (10 mg, 0.09 mmol, 1.00 equiv.) and HNTf$_2$ (25 mg, 0.09 mmol, 1.00 equiv.) were dissolved in C_6D_6 (0.5 mL) and heated at 60 °C for 3 hours. NMR experiments confirmed the full conversion to a new product, which was assigned to be the adduct **8**.

^{1}H NMR (500 MHz, C_6D_6, 300 K) δ 3.86 (br, s, 1H, (^{i}Pr)$_2$N*H*), 3.21-2.31 (br, m, 2H, B*H*$_2$), 2.92 (sep, $^3J_{HH}$ = 6.8 Hz, 2H, C*H*(CH$_3$)$_2$), 0.77 (d, $^3J_{HH}$ = 6.6 Hz, 6H, CH(C*H*$_3$)$_2$), 0.59 (d, $^3J_{HH}$ = 6.8 Hz, 6H, CH(C*H*$_3$)$_2$).

^{1}H {^{11}B} NMR (500 MHz, C_6D_6, 300 K) δ 3.86 (br, s, 1H, (^{i}Pr)$_2$N*H*), 2.91 (sep, $^{3}J_{HH}$ = 6.8 Hz, 2H, C*H*(CH_3)$_2$), 2.77 (br, s, 2H, B*H*$_2$), 0.77 (d, $^{3}J_{HH}$ = 6.6 Hz, 6H, CH(C*H*$_3$)$_2$), 0.58 (d, $^{3}J_{HH}$ = 6.8 Hz, 6H, CH(C*H*$_3$)$_2$).

^{11}B NMR (160 MHz, C_6D_6, 300 K) δ –9.5 (br, t, $^{1}J_{BH}$ = 123 Hz, *B*H$_2$).

^{11}B {^{1}H} NMR (160 MHz, C_6D_6, 300 K) δ –9.5 (s, *B*H$_2$).

^{13}C NMR (151 MHz, C_6D_6, 300 K) δ 120.26 (q, $^{1}J_{CF}$ = 325 Hz, *C*F$_3$), 48.92 (s, *C*H(CH_3)$_2$), 18.99 (s, CH(*C*H$_3$)$_2$), 18.77 (s, CH(*C*H$_3$)$_2$).

^{19}F NMR (471 MHz, C_6D_6, 300 K) δ –71.4 (s, C*F*$_3$).

3.2 Generation of the monomeric borocation (^{i}Pr)$_2$NBHNTf$_2$ 9

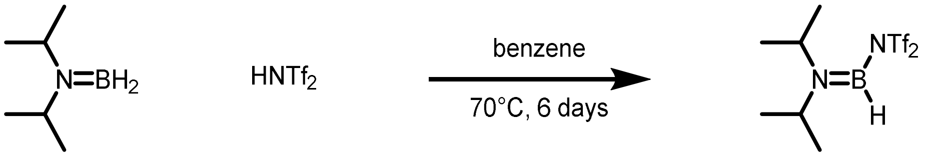

A solution of HNTf$_2$ (1.24 g, 4.40 mmol, 1 equiv.) in benzene (10 mL) was added to a solution of (^{i}Pr)$_2$NBH$_2$ (0.50 g, 4.40 mmol, 1.00 equiv.) in benzene (10 mL) at room temperature. The solution was heated at 70 °C in an unpressurised system (open Schlenk under N_2) for 6 days and monitored periodically by NMR spectroscopy. Very slow conversion of the adduct **8** to the product **9** was observed and after 6 days the reaction mixture was filtered, despite not being completed (2.2:1, **8:9**). Volatiles were removed under vacuum affording a waxy colourless solid. The solid was dissolved in pentane, cooled down to −78 °C and filtered at this temperature. Removal of pentane fraction afforded the product as a volatile colourless crystalline solid, contaminated with only trace of impurities (however, the impurities in the 19F are sufficient that it is not appropriate to report a yield). Partial decomposition was observed after leaving **9** in solution overnight.

^{1}H NMR (500 MHz, C_6D_6, 300 K) δ 5.16-3.74 (br, m, 1H, B*H*), 4.00 (sep, $^{3}J_{HH}$ = 6.6 Hz, 1H, C*H*(CH_3)$_2$), 2.77 (sep, $^{3}J_{HH}$ = 6.8 Hz, 1H, C*H*(CH_3)$_2$), 0.92 (d, $^{3}J_{HH}$ = 6.8 Hz, 1H, CH(C*H*$_3$)$_2$), 0.79 (d, $^{3}J_{HH}$ = 6.6 Hz, 1H, CH(C*H*$_3$)$_2$).

^{11}B NMR (160 MHz, C_6D_6, 300 K) δ 28.9 (d, $^{1}J_{BH}$ = 170 Hz, *B*H).

^{11}B {^{1}H} NMR (160 MHz, C_6D_6, 300 K) δ 28.9 (s, *B*H).

^{13}C NMR (126 MHz, C_6D_6, 300 K) δ 119.65 (q, $^{1}J_{CF}$ = 325 Hz, *C*F$_3$), 49.52 (s, *C*H(CH_3)$_2$), 45.58 (s, *C*H(CH_3)$_2$), 26.40 (s, CH(*C*H$_3$)$_2$), 20.53 (s, CH(*C*H$_3$)$_2$).

^{19}F NMR (471 MHz, C_6D_6, 300 K) δ –73.2 (s, C*F*$_3$).

Mass Spectrum: HRMS (ESI+) m/z calculated for $C_8H_{15}BF_6N_2O_4S_2$: 392.04650; Found: 392.04539.

3.3 NMR Spectra of $[Me_2NBH_2]_2$ 1

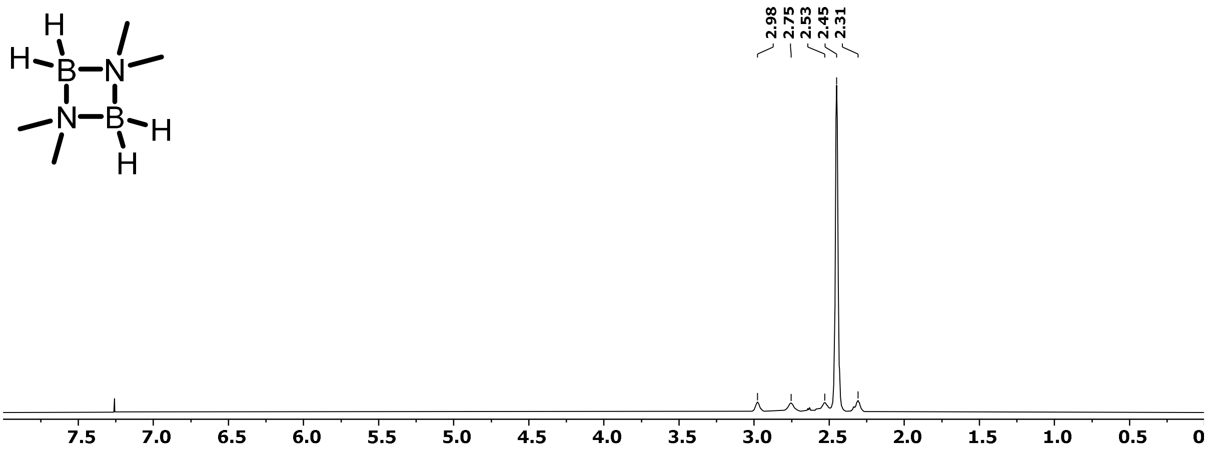

Figure S2: ^{1}H NMR spectrum (500 MHz, $CDCl_3$, 300 K) of $[Me_2NBH_2]_2$ **1**.

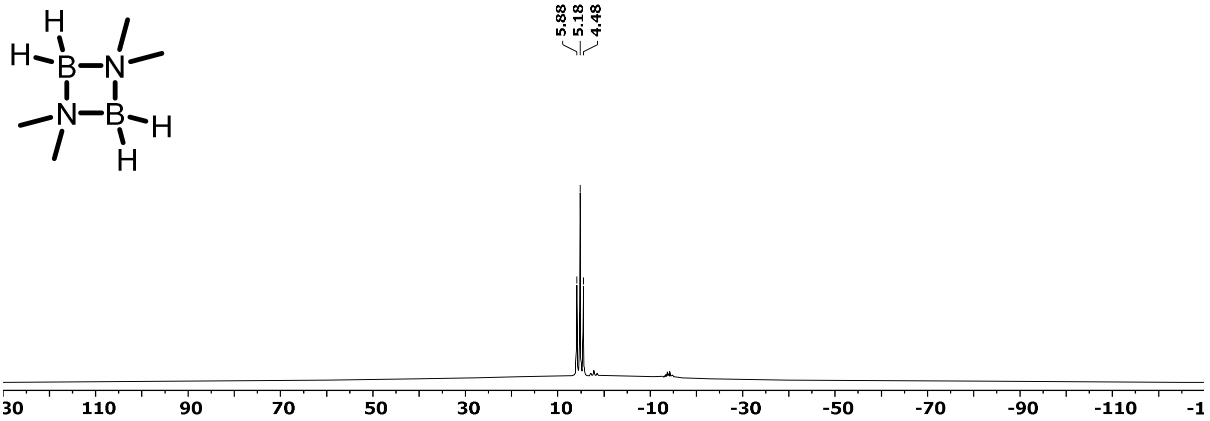

Figure S3: ^{11}B NMR spectrum (160 MHz, $CDCl_3$, 300 K) of $[Me_2NBH_2]_2$ **1**.

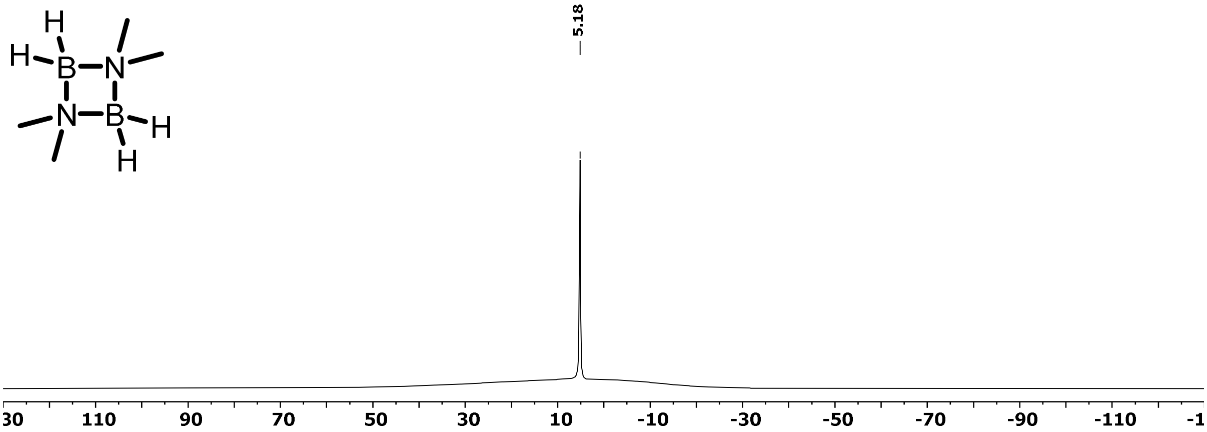

Figure S4: $^{11}B\{^{1}H\}$ NMR spectrum (160 MHz, $CDCl_3$, 300 K) of $[Me_2NBH_2]_2$ **1**.

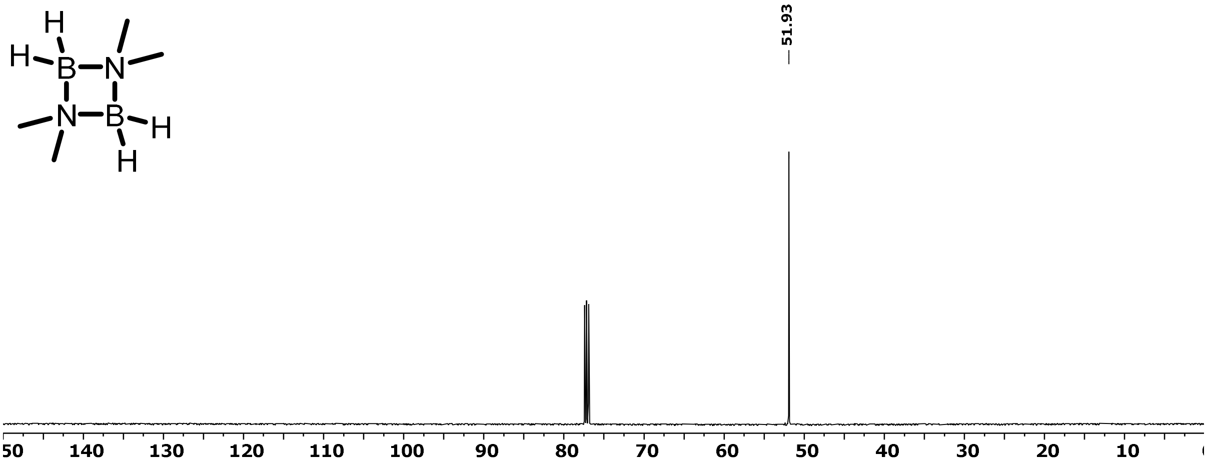

Figure S5: $^{13}C\{^{1}H\}$ NMR spectrum (126 MHz, $CDCl_3$, 300 K) of $[Me_2NBH_2]_2$ **1**.

3.4 NMR Spectra of [Pyrrolidine-BH$_2$]$_2$ 2

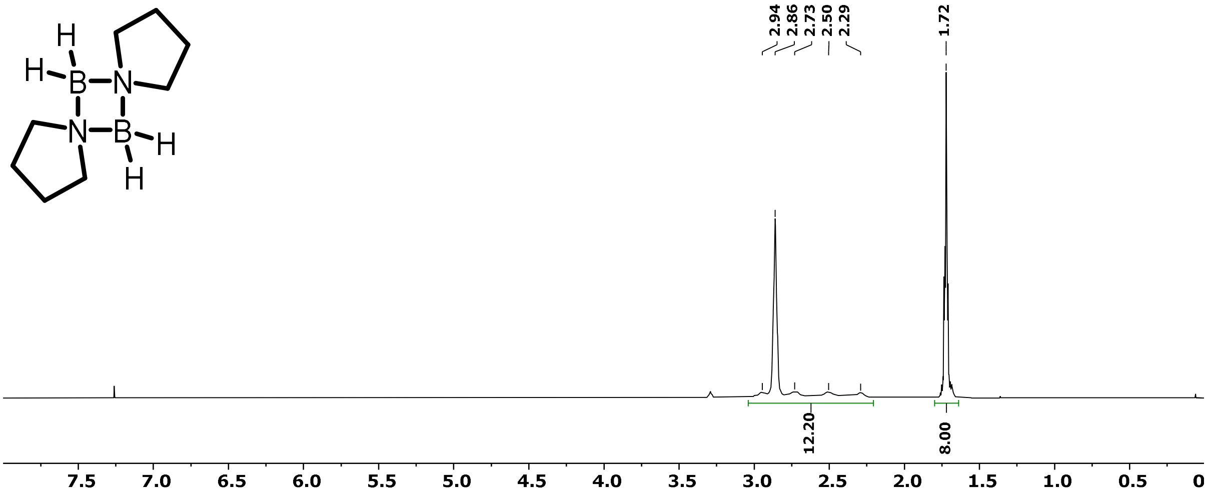

Figure S6: ^{1}H NMR spectrum (500 MHz, $CDCl_3$, 300 K) of [Pyrrolidine-BH$_2$]$_2$ **2**.

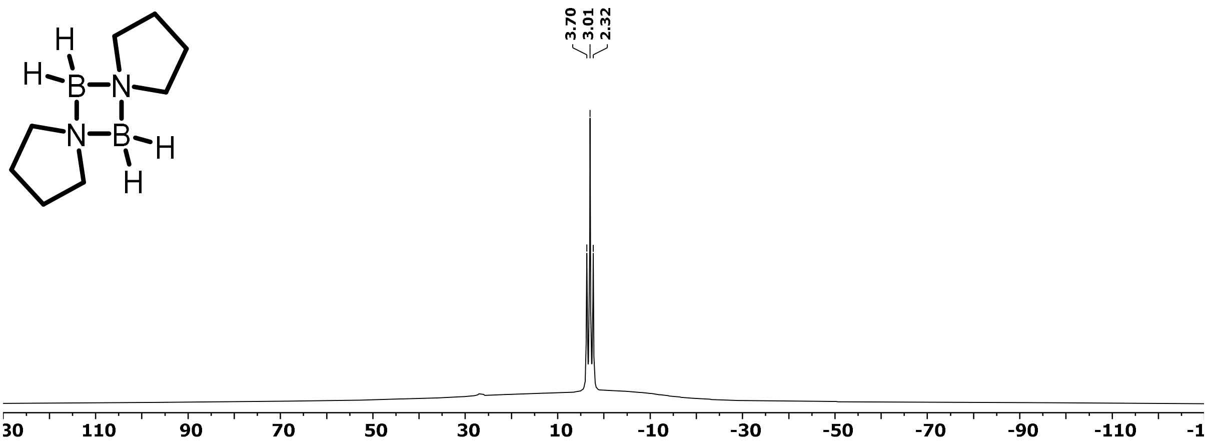

Figure S7: ^{11}B NMR spectrum (160 MHz, $CDCl_3$, 300 K) of [Pyrrolidine-BH_2]$_2$ **2.**

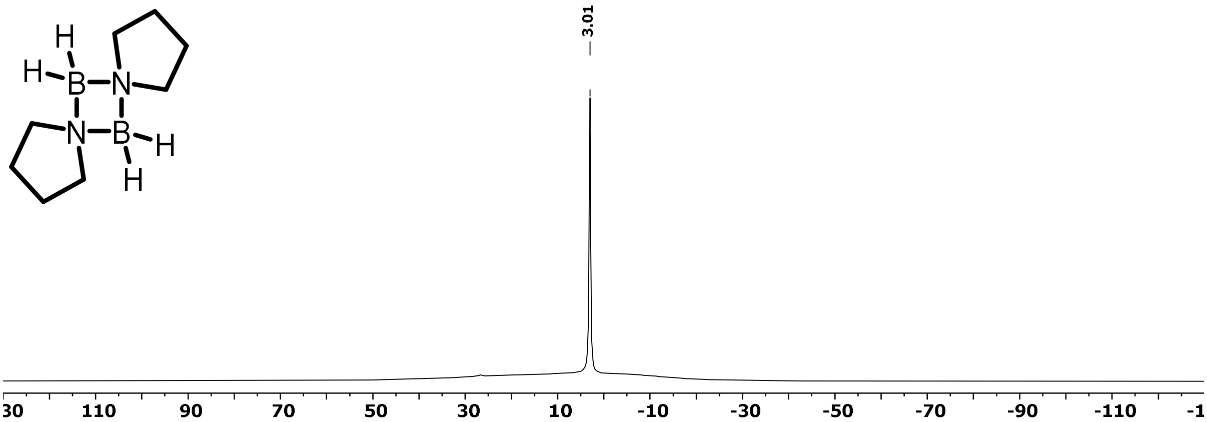

Figure S8: ^{11}B {^{1}H} NMR spectrum (160 MHz, $CDCl_3$, 300 K) of [Pyrrolidine-BH_2]$_2$ **2.**

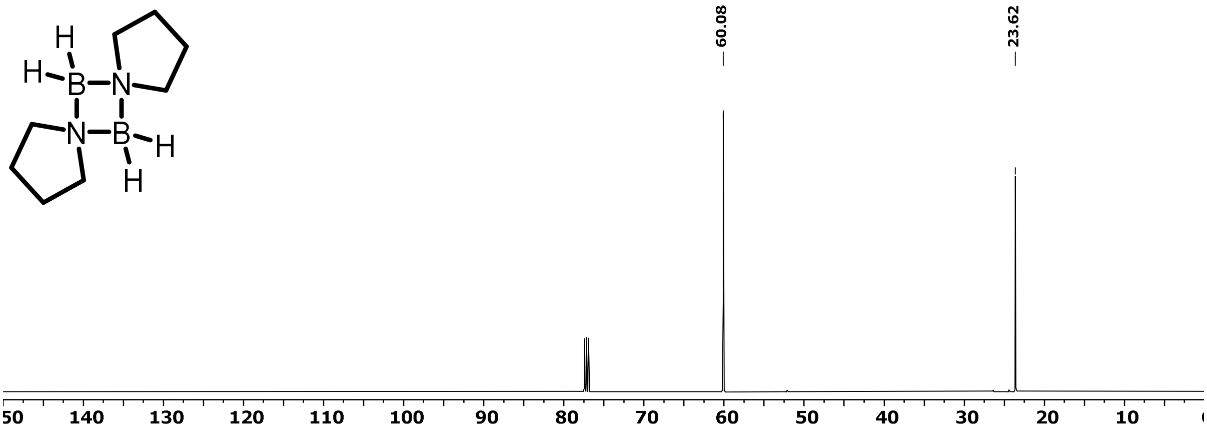

Figure S9: ^{13}C {^{1}H} NMR spectrum (126 MHz, $CDCl_3$, 300 K) of [Pyrrolidine-BH_2]$_2$ **2.**

3.5 **NMR Spectra of $H_2B(\mu\text{-}Me_2N)_2BH(I)$ 3**

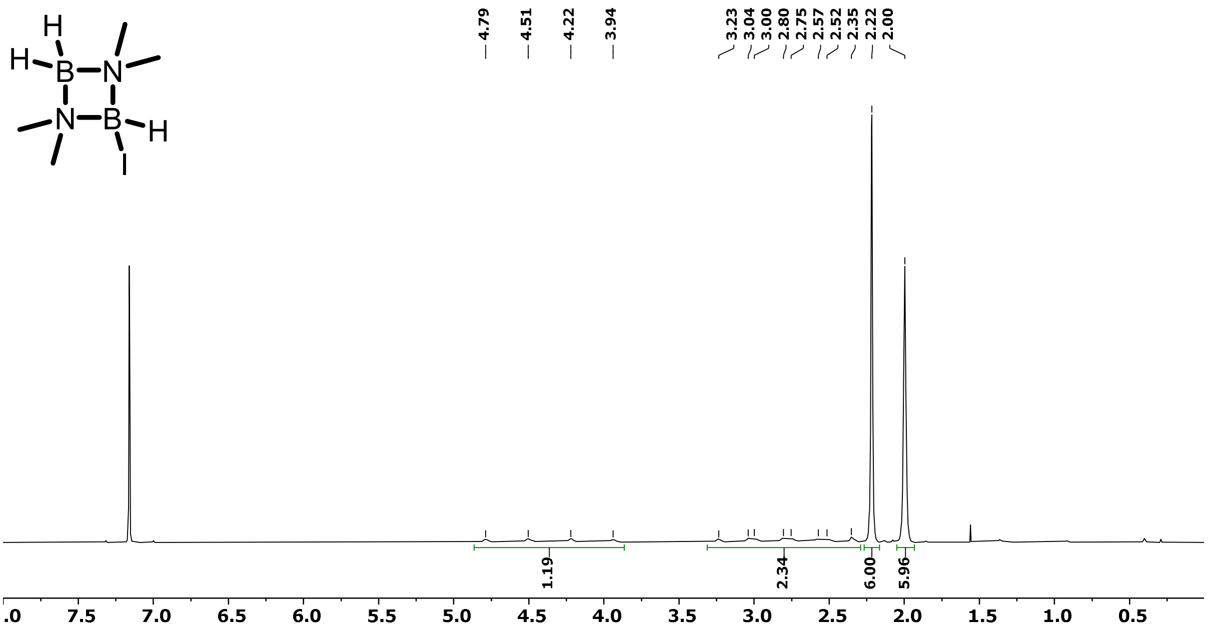

Figure S10: ^{1}H NMR spectrum (500 MHz, C_6D_6, 300 K) of $H_2B(\mu\text{-}Me_2N)_2BH(I)$ **3**.

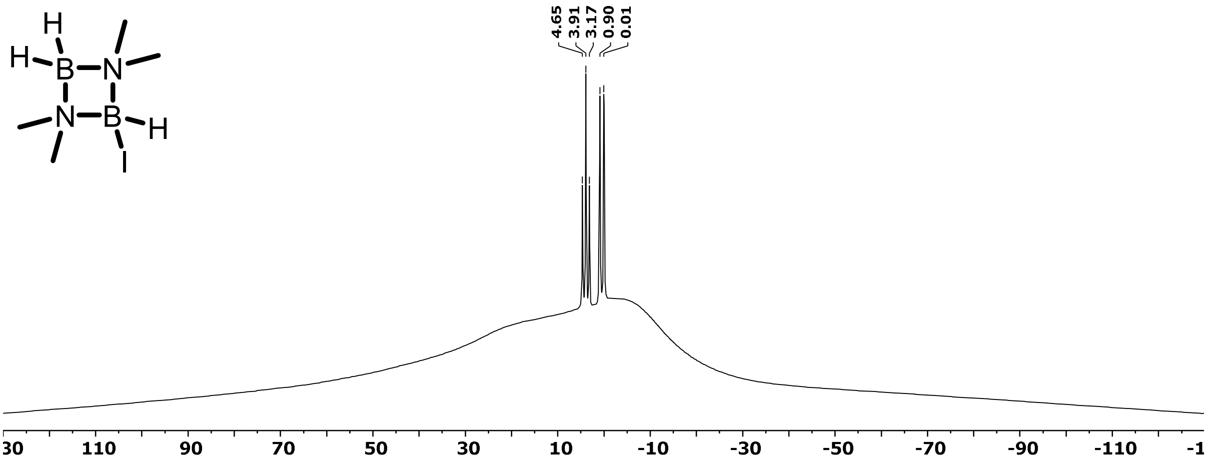

Figure S11: ^{11}B NMR spectrum (160 MHz, C_6D_6, 300 K) of $H_2B(\mu\text{-}Me_2N)_2BH(I)$ **3**.

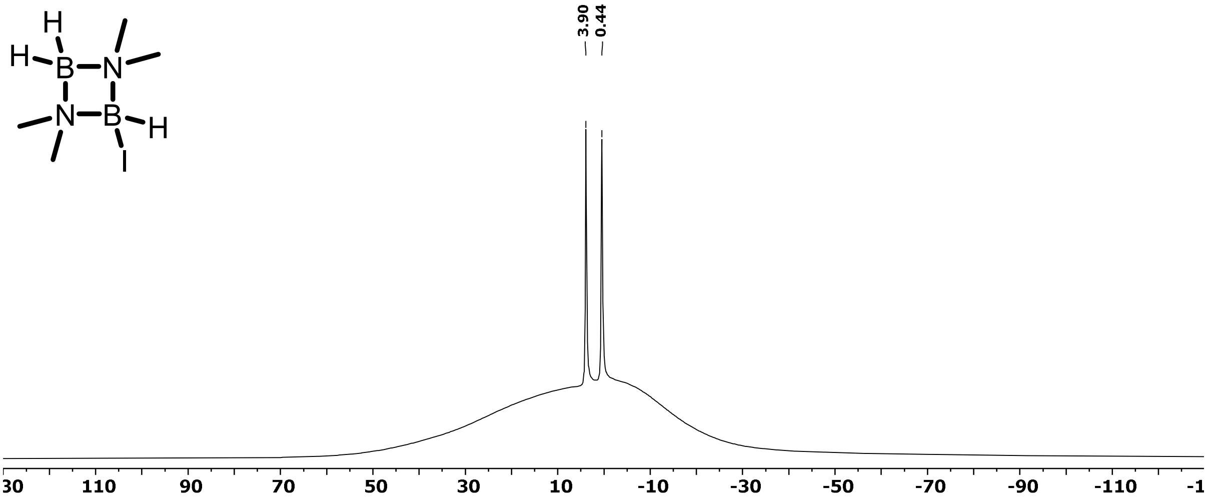

Figure S12: ^{11}B {^{1}H} NMR spectrum (160 MHz, C_6D_6, 300 K) of $H_2B(\mu\text{-}Me_2N)_2BH(I)$ **3**.

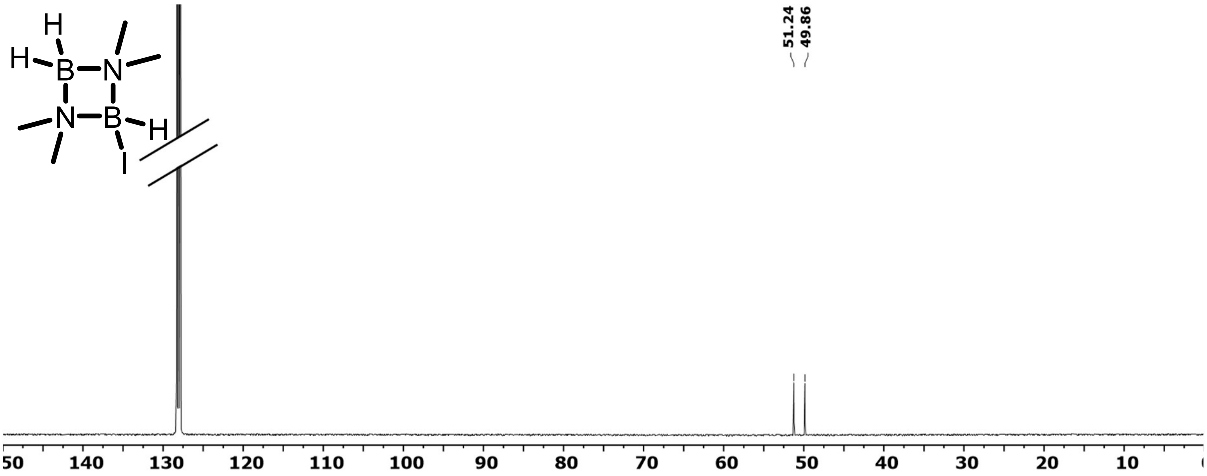

Figure S13: ^{13}C {^{1}H} NMR spectrum (126 MHz, C_6D_6, 300 K) of $H_2B(\mu\text{-}Me_2N)_2BH(I)$ **3**.

3.6 NMR Spectra of $H_2B(\mu\text{-}Me_2N)_2BH(OSOCF_3NTf)$ 4

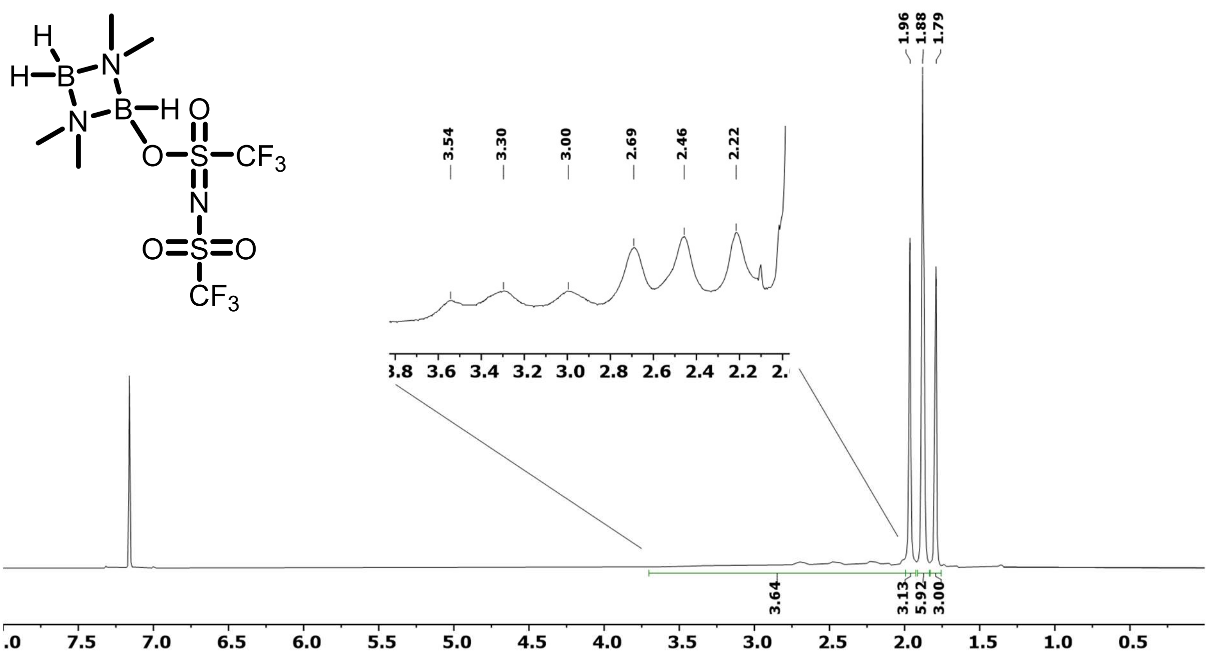

Figure S14: ^1H NMR spectrum (500 MHz, C_6D_6, 300 K) of $H_2B(\mu\text{-}Me_2N)_2BH(OSOCF_3NTf)$ **4**.

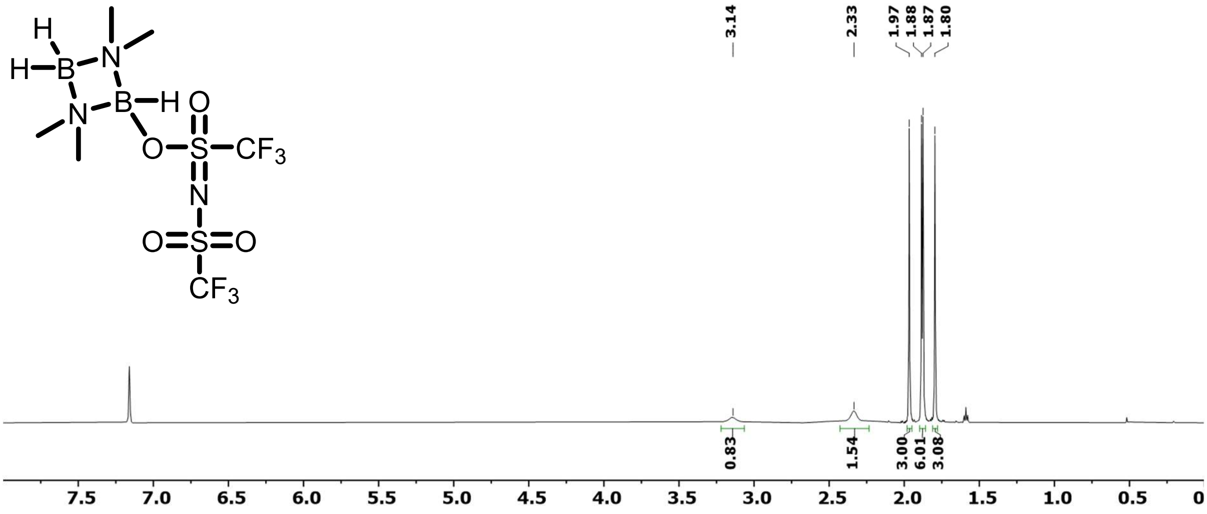

Figure S15: $^1H\{^{11}B\}$ NMR spectrum (500 MHz, C_6D_6, 300 K) of $H_2B(\mu\text{-}Me_2N)_2BH(OSOCF_3NTf)$ **4.**

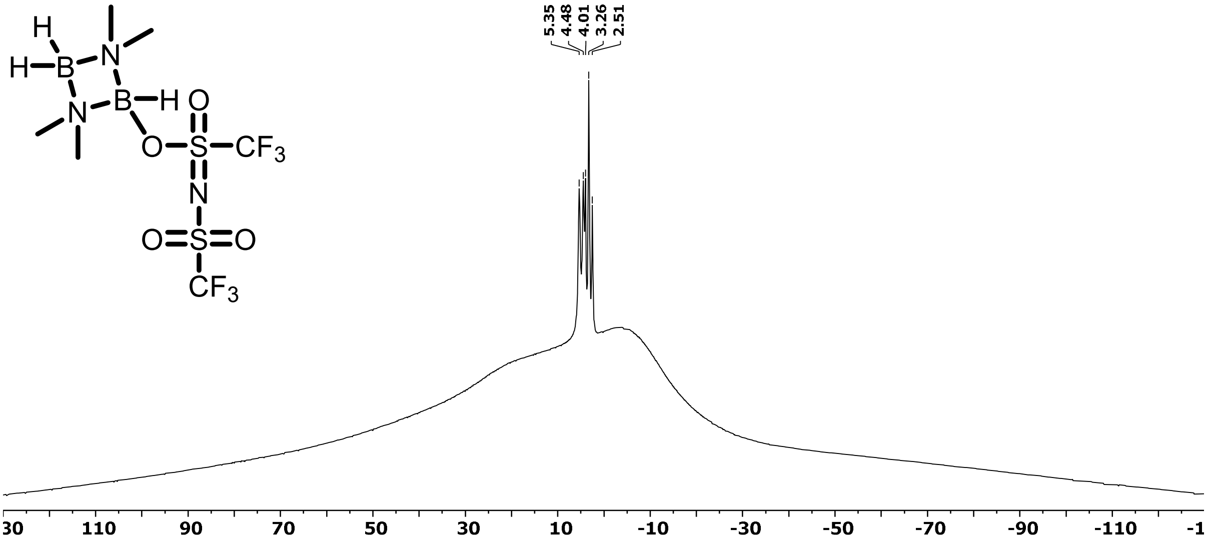

Figure S16: ^{11}B NMR spectrum (160 MHz, C_6D_6, 300 K) of $H_2B(\mu\text{-}Me_2N)_2BH(OSOCF_3NTf)$ **4**.

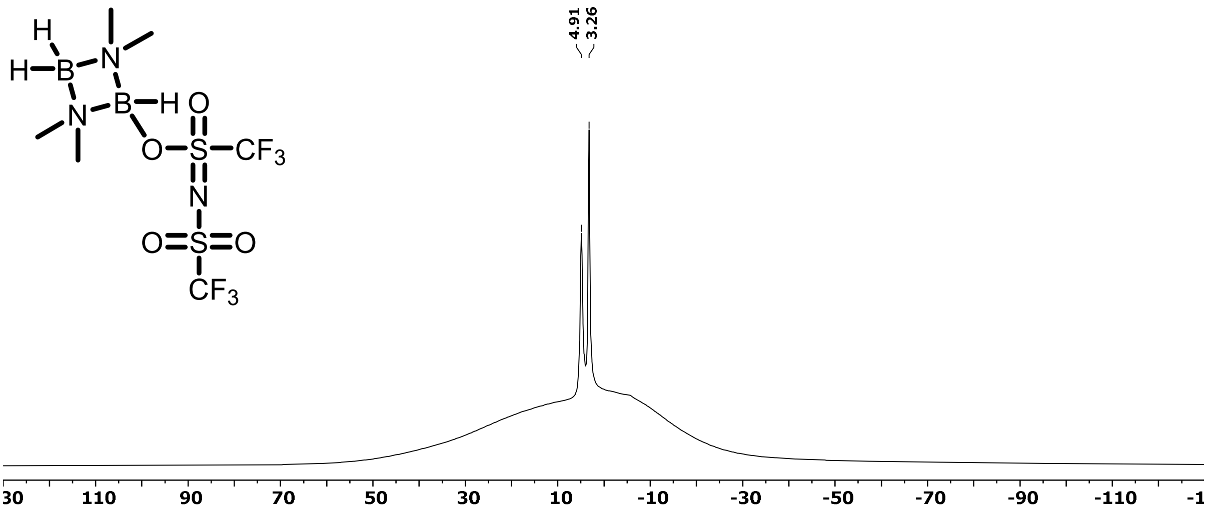

Figure S17: ^{11}B {^{1}H} NMR spectrum (160 MHz, C_6D_6, 300 K) of $H_2B(\mu\text{-}Me_2N)_2BH(OSOCF_3NTf)$ **4**.

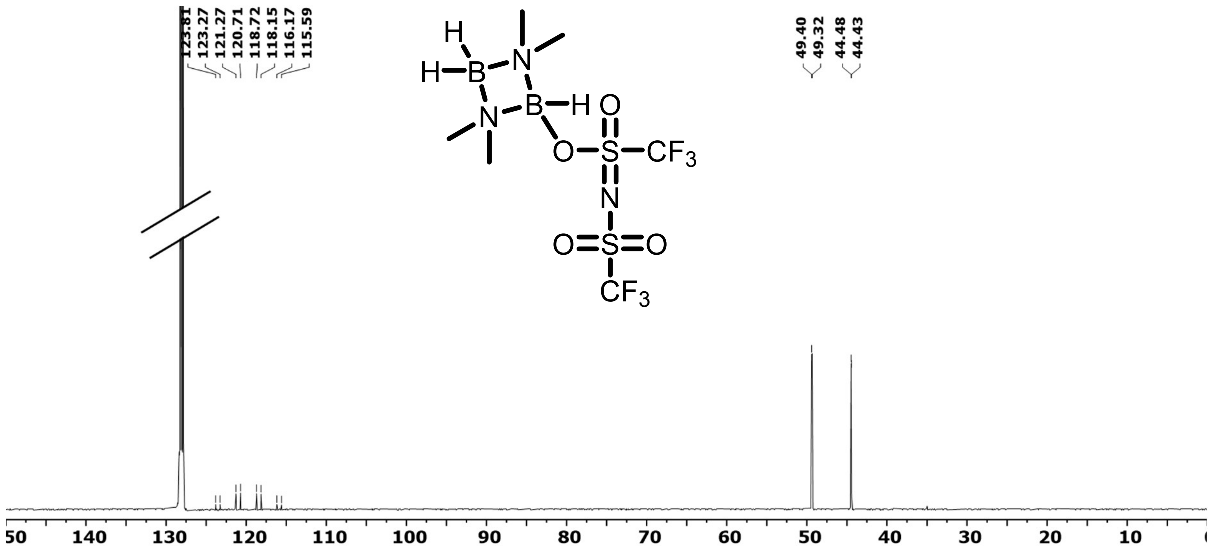

Figure S18: ^{13}C {^{1}H} NMR spectrum (126 MHz, C_6D_6, 300 K) of $H_2B(\mu\text{-}Me_2N)_2BH(OSOCF_3NTf)$ **4**.

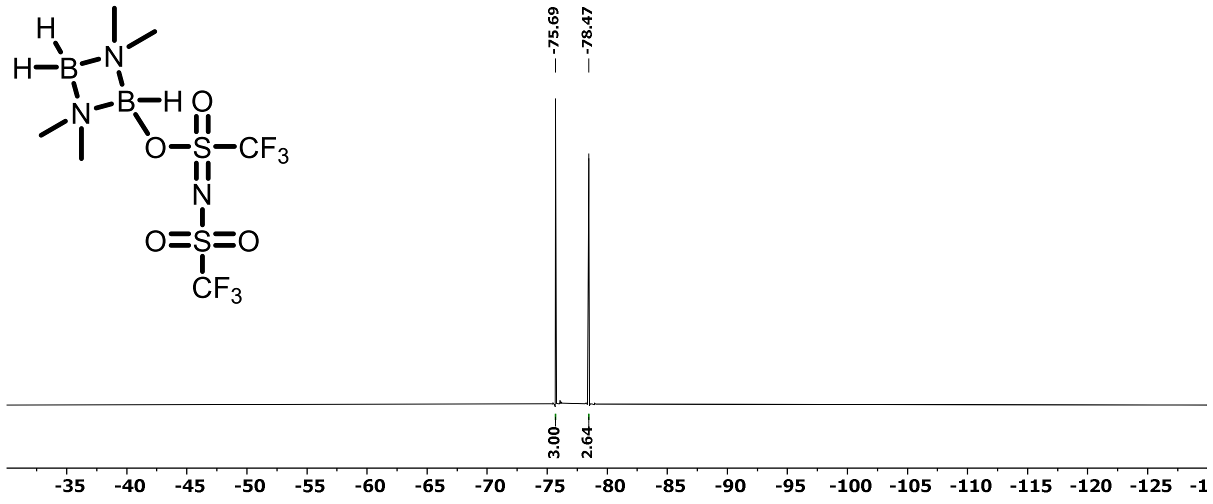

Figure S19: ^{19}F NMR spectrum (471 MHz, C_6D_6, 300 K) of $H_2B(\mu\text{-}Me_2N)_2BH(OSOCF_3NTf)$ **4**.

3.7 **NMR Spectra of H_2B(μ-pyrrolidine)$_2BH(OSOCF_3NTf)$ 5**

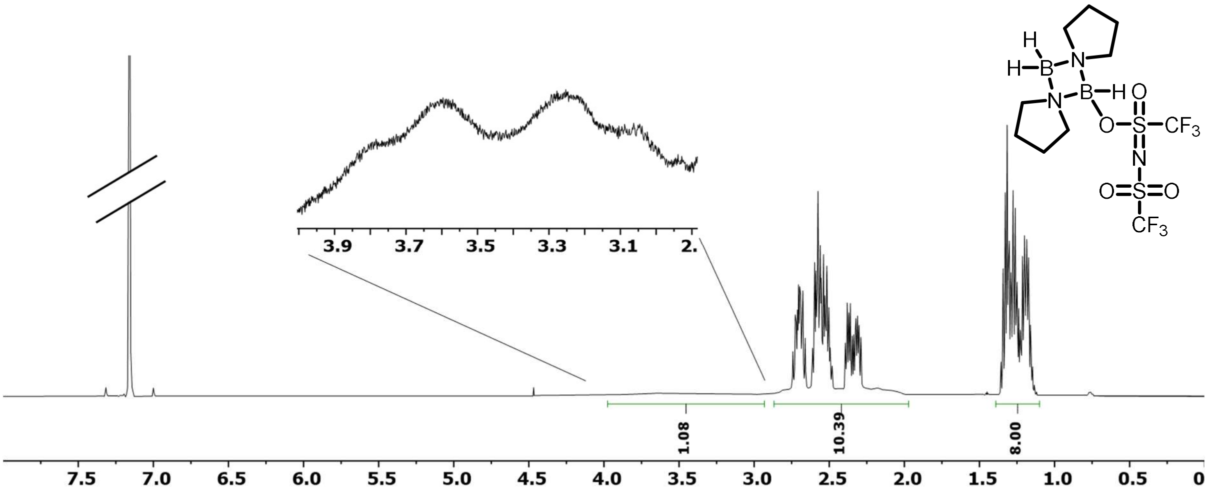

Figure S20: ^{1}H NMR spectrum (500 MHz, C_6D_6, 300 K) of H_2B(μ-pyrrolidine)$_2BH(OSOCF_3NTf)$ **5**.

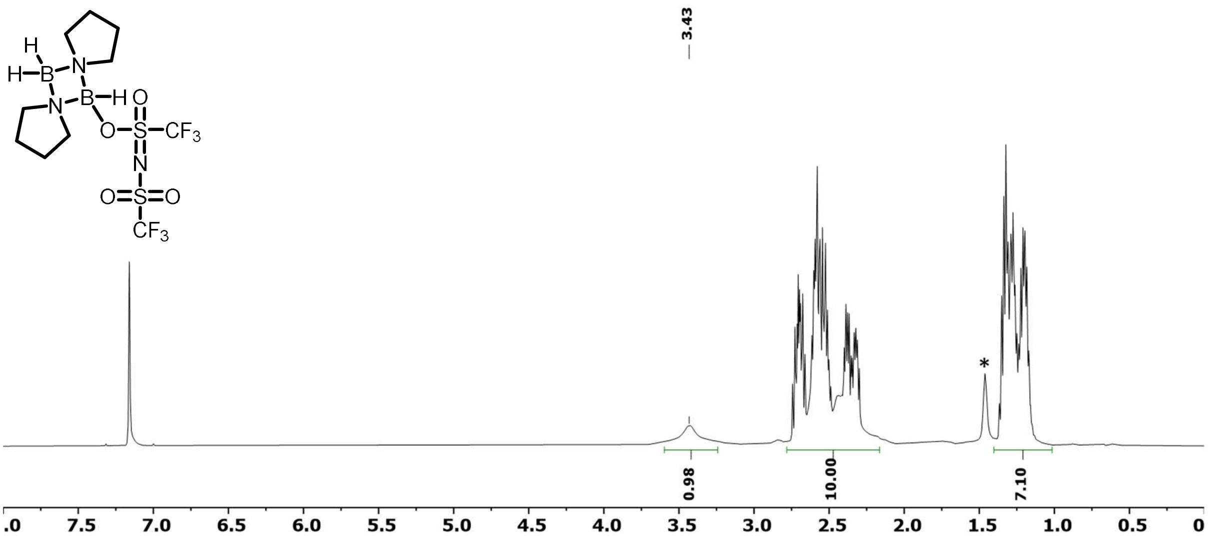

Figure S21: ^{1}H {^{11}B} NMR spectrum (500 MHz, C_6D_6, 300 K) of H_2B(μ-pyrrolidine)$_2BH(OSOCF_3NTf)$ **5**. * This peak is due to decomposition of **5**.

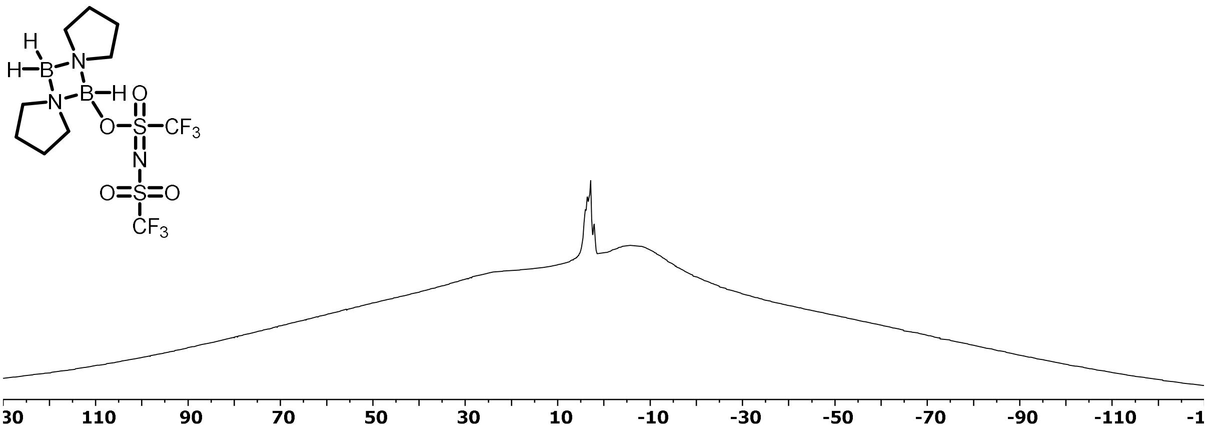

Figure S22: ^{11}B NMR spectrum (160 MHz, C_6D_6, 300 K) of H_2B(μ-pyrrolidine)$_2$BH(OSOCF$_3$NTf) **5**.

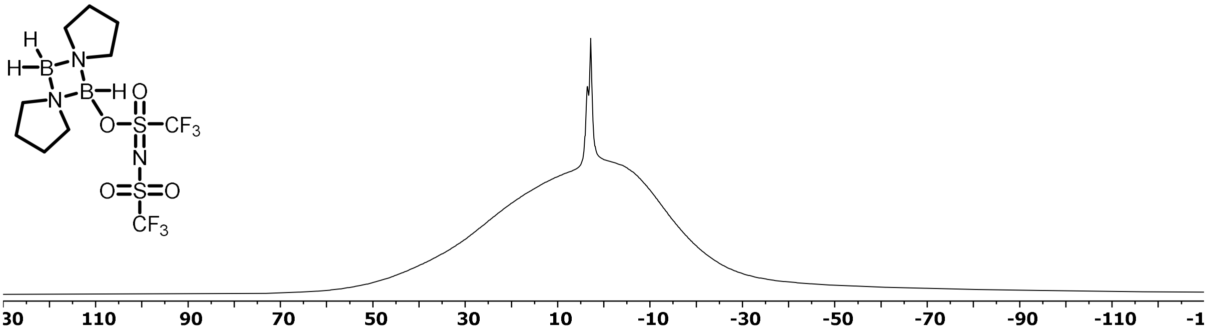

Figure S23: ^{11}B {^1H} NMR spectrum (160 MHz, C_6D_6, 300 K) of H_2B(μ-pyrrolidine)$_2$BH(OSOCF$_3$NTf) **5**.

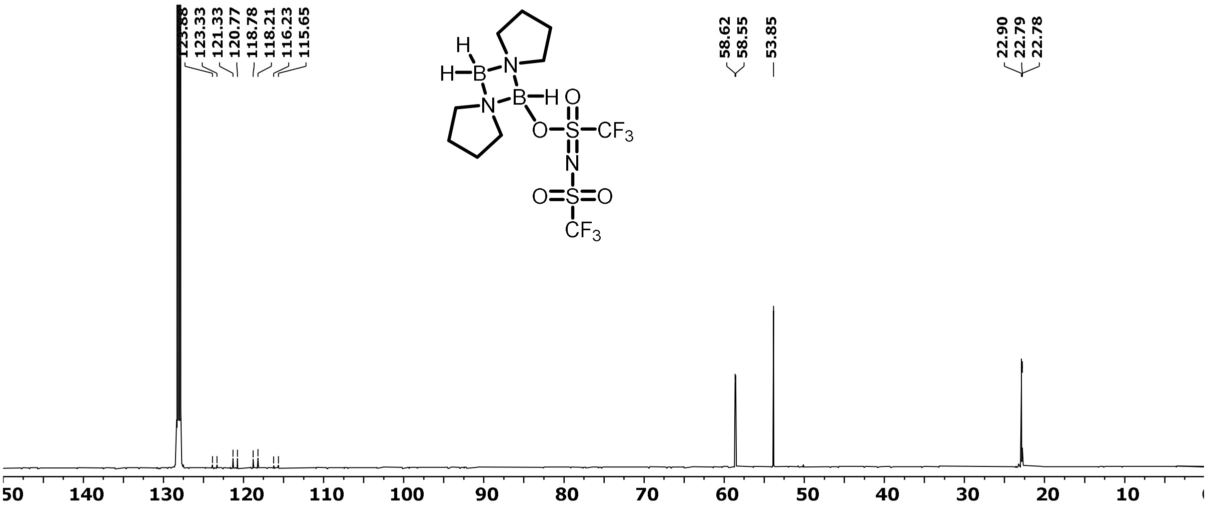

Figure S24: ^{13}C {^1H} NMR spectrum (126 MHz, C_6D_6, 300 K) of H_2B(μ-pyrrolidine)$_2$BH(OSOCF$_3$NTf) **5**.

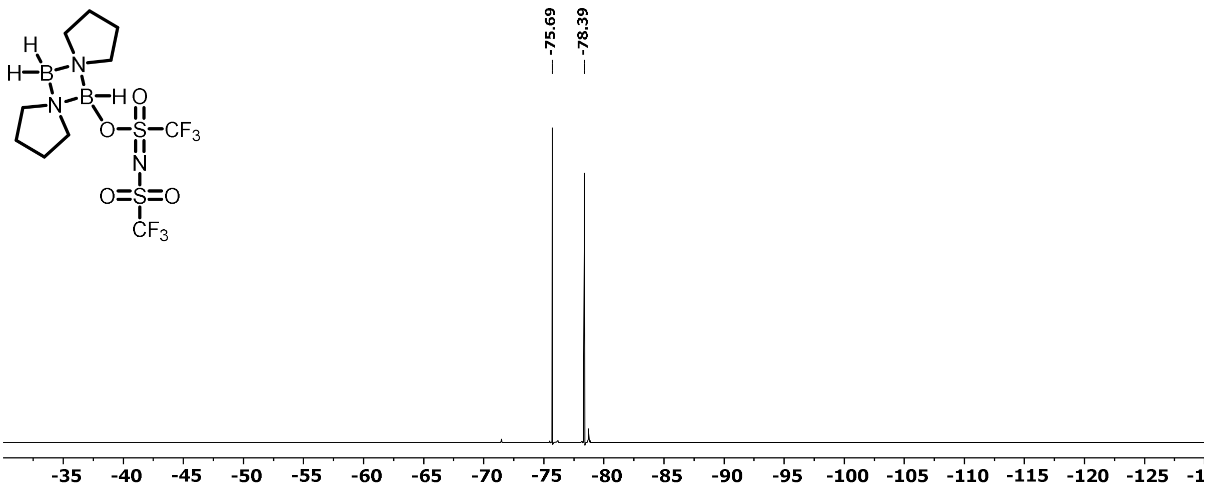

Figure S25: ^{19}F NMR spectrum (471 MHz, C_6D_6, 300 K) of H_2B(µ-pyrrolidine)$_2$BH($OSOCF_3NTf$) **5**.

3.8 **NMR Spectra of $[(Me_2N)_3B_3H_5][B(C_6F_5)_4]$ Cation 7**

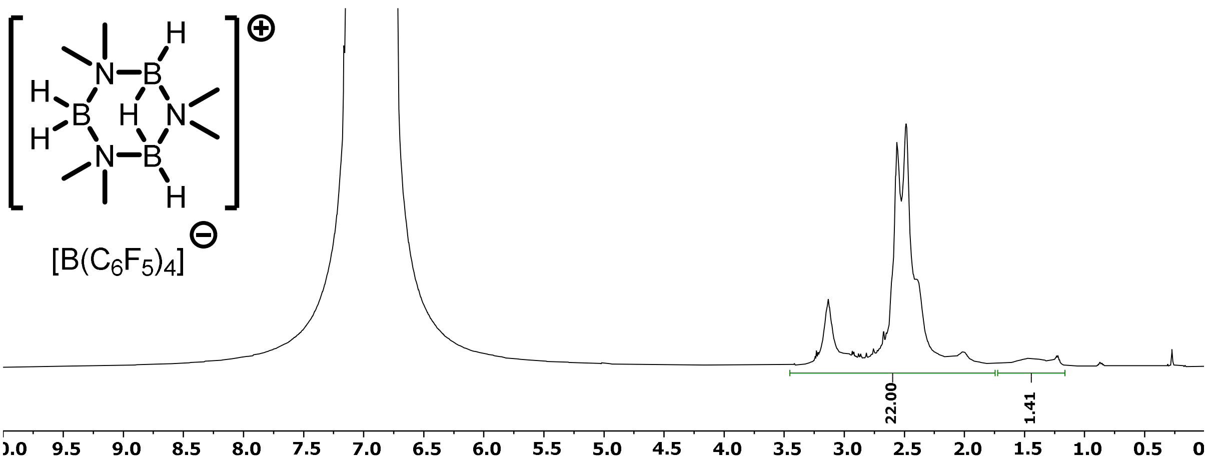

Figure S26: ^{1}H NMR spectrum (500 MHz, $C_6H_4F_2$, **300 K**) of $[(Me_2N)_3B_3H_5][B(C_6F_5)_4]$ **7**.

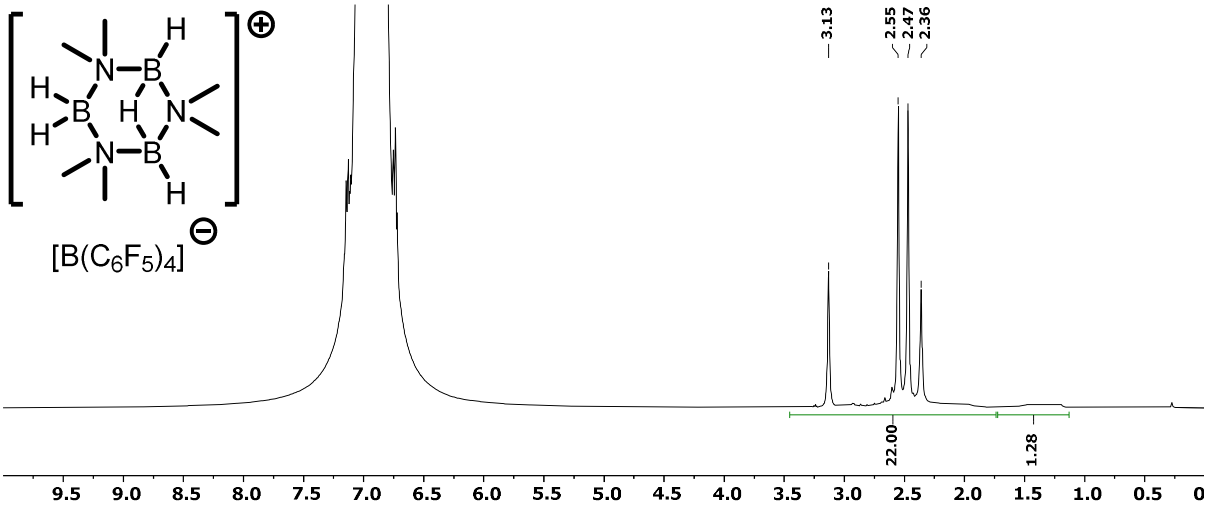

Figure S27: ^{1}H NMR spectrum (500 MHz, $C_6H_4F_2$, **278 K**) of $[(Me_2N)_3B_3H_5][B(C_6F_5)_4]$ **7**.

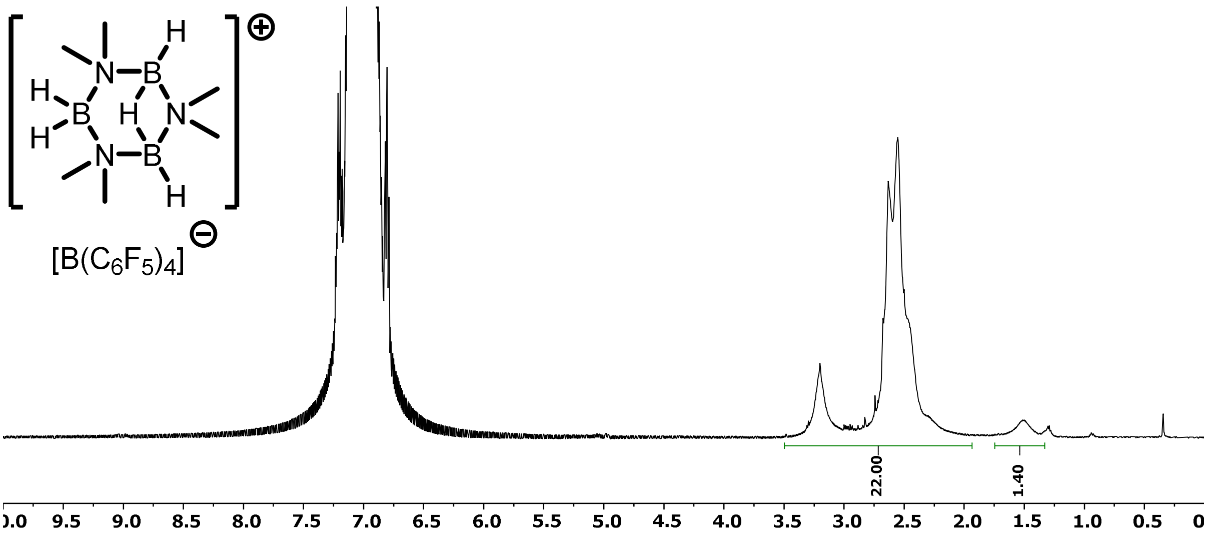

Figure S28: $^{1}H\{^{11}B\}$ NMR spectrum (500 MHz, $C_6H_4F_2$, 300 K) of $[(Me_2N)_3B_3H_5][B(C_6F_5)_4]$ **7**.

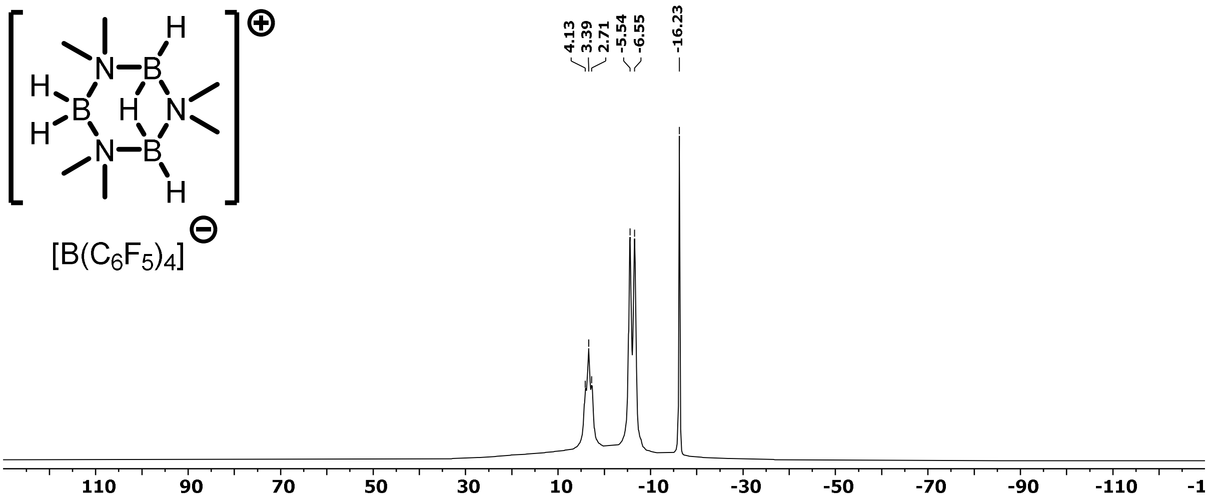

Figure S29: ^{11}B NMR spectrum (160 MHz, $C_6H_4F_2$, 300 K) of $[(Me_2N)_3B_3H_5][B(C_6F_5)_4]$ **7**.

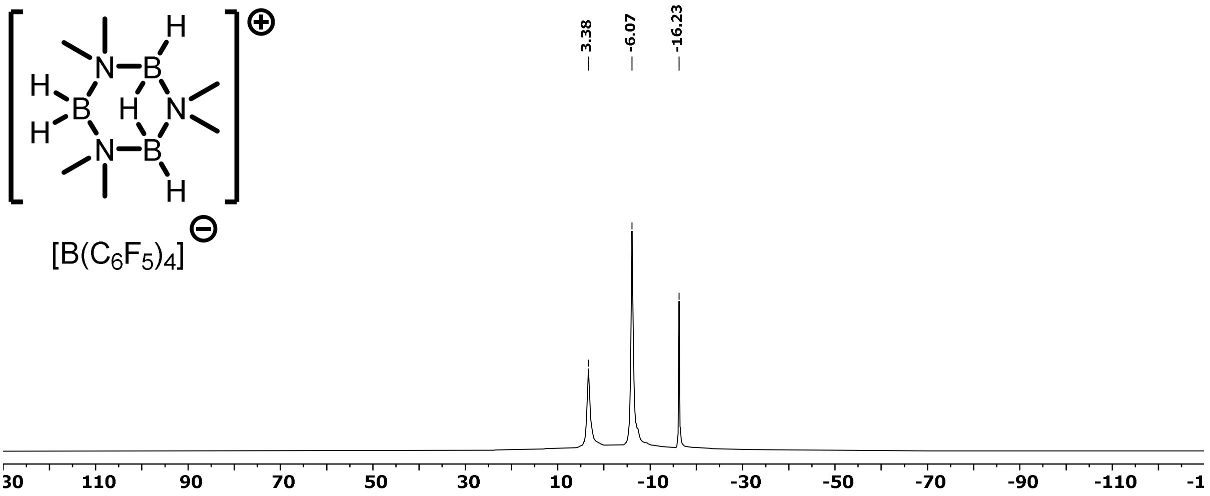

Figure S30: $^{11}B\{^{1}H\}$ NMR spectrum (160 MHz, $C_6H_4F_2$, 300 K) of $[(Me_2N)_3B_3H_5][B(C_6F_5)_4]$ **7**.

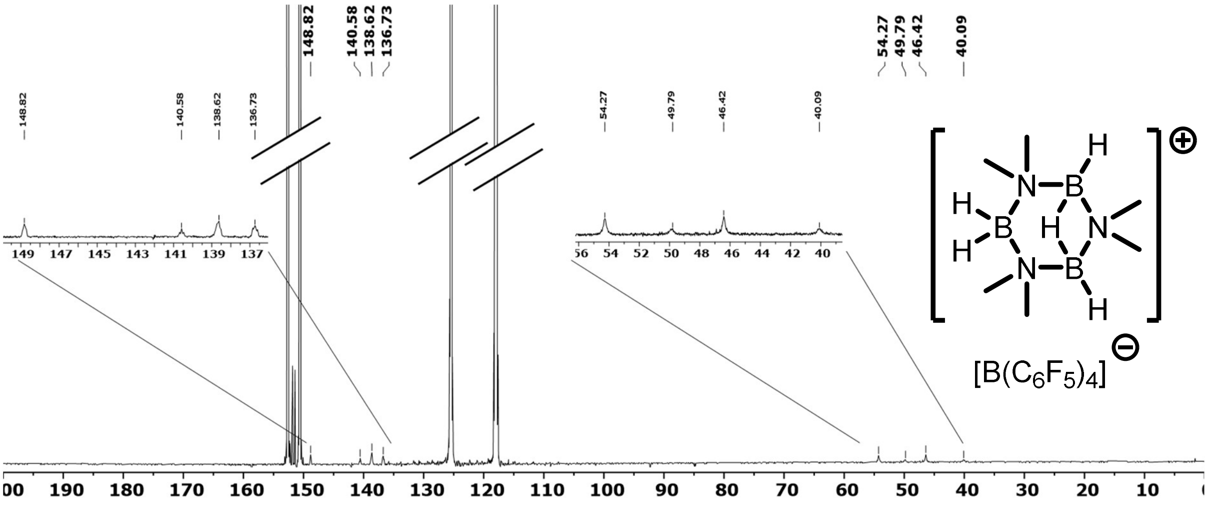

Figure S31: $^{13}C\{^{1}H\}$ NMR spectrum (126 MHz, $C_6H_4F_2$, 300 K) of $[(Me_2N)_3B_3H_5][B(C_6F_5)_4]$ **7**. The intense peaks at δ 152.62, 150.64, 125.46, 117.98 ppm are due to the solvent $C_6H_4F_2$.

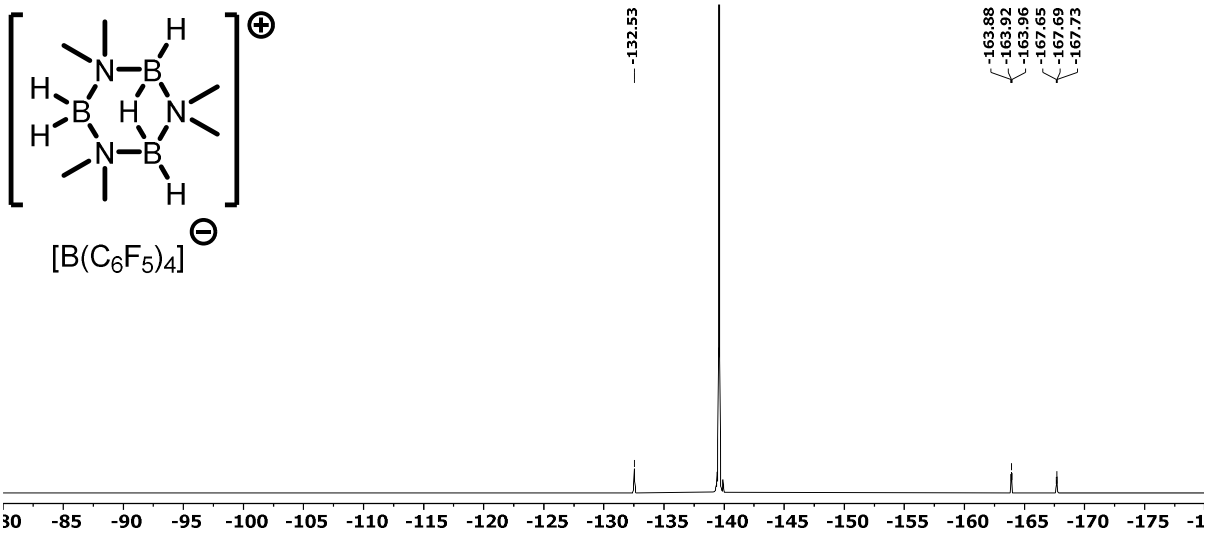

Figure S32: ^{19}F NMR spectrum (471 MHz, $C_6H_4F_2$, 300 K) of $[(Me_2N)_3B_3H_5][B(C_6F_5)_4]$ **7**. The intense peak at δ –139 ppm is due to the solvent $C_6H_4F_2$.

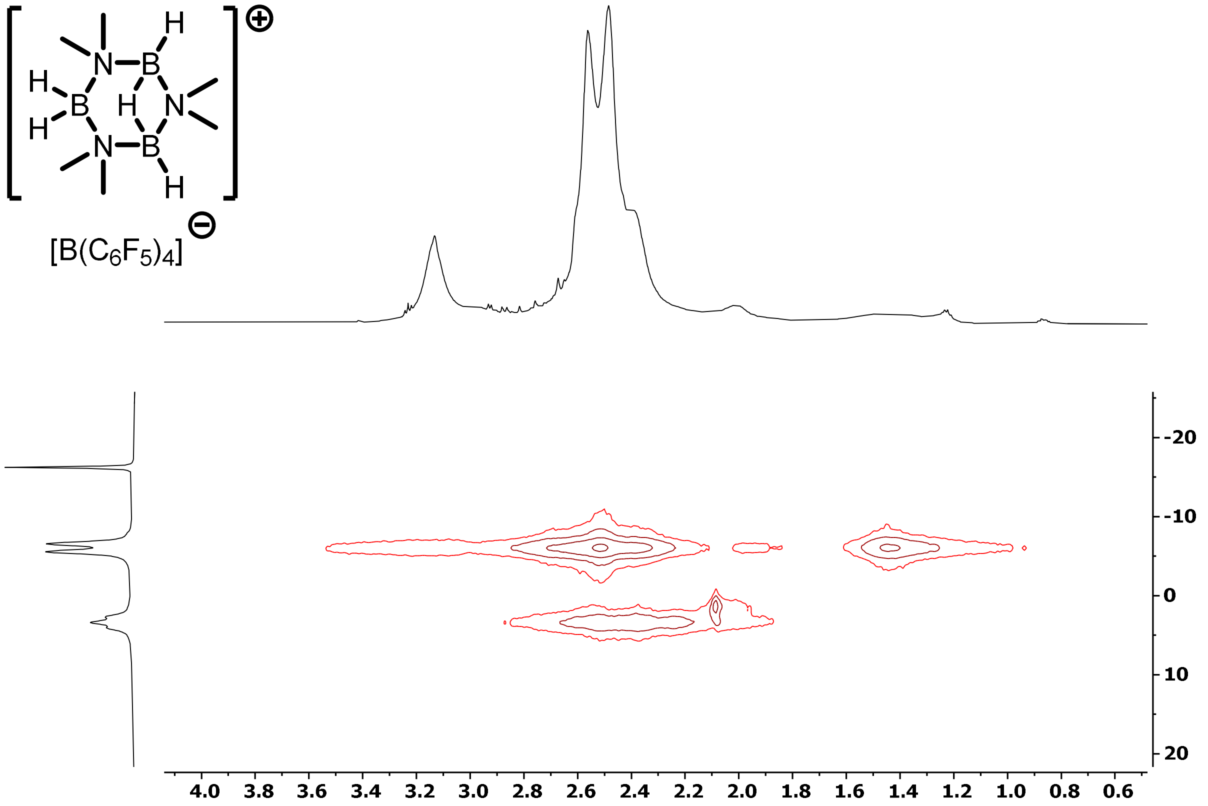

Figure S33: 2-D ^{11}B–^{1}H HMQC NMR spectrum (160–500 MHz, $C_6H_4F_2$, 300 K) of $[(Me_2N)_3B_3H_5][B(C_6F_5)_4]$ **7**.

3.9 Reference NMR Spectra of $SiMe_4$ in difluorobenzene ($C_6H_4F_2$)

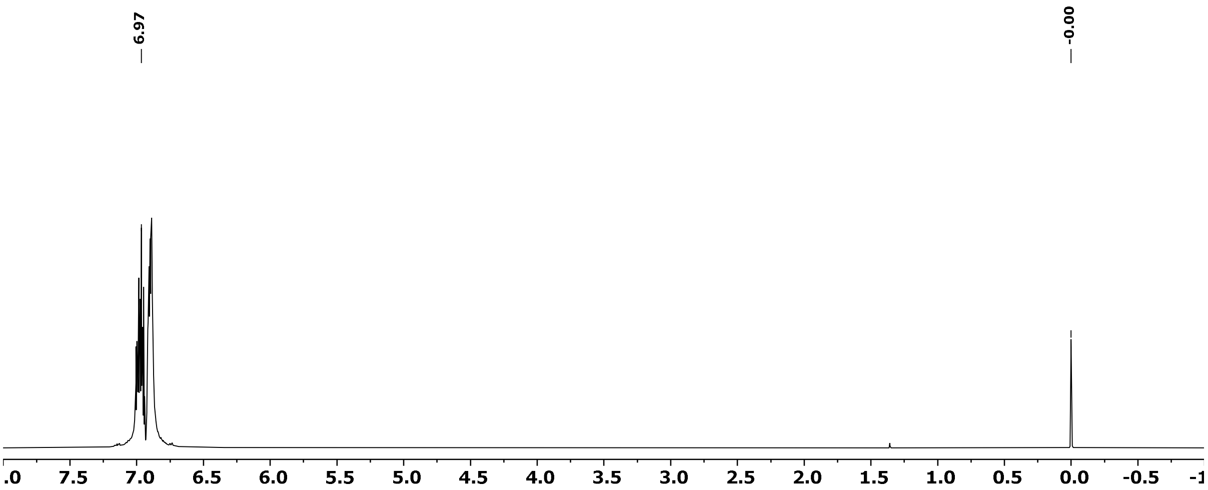

Figure S34: ^1H NMR spectrum (500 MHz, $C_6H_4F_2$, 300 K) of $SiMe_4$.

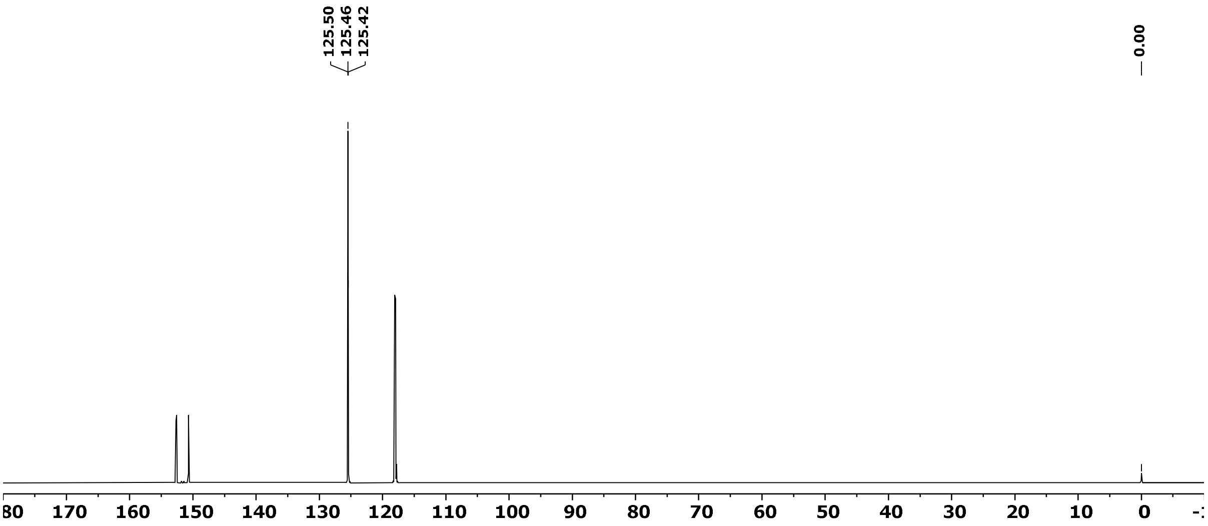

Figure S35: ^{13}C NMR spectrum (126 MHz, $C_6H_4F_2$, 300 K) of $SiMe_4$.

3.10 **FTIR Spectra of boron electrophiles**

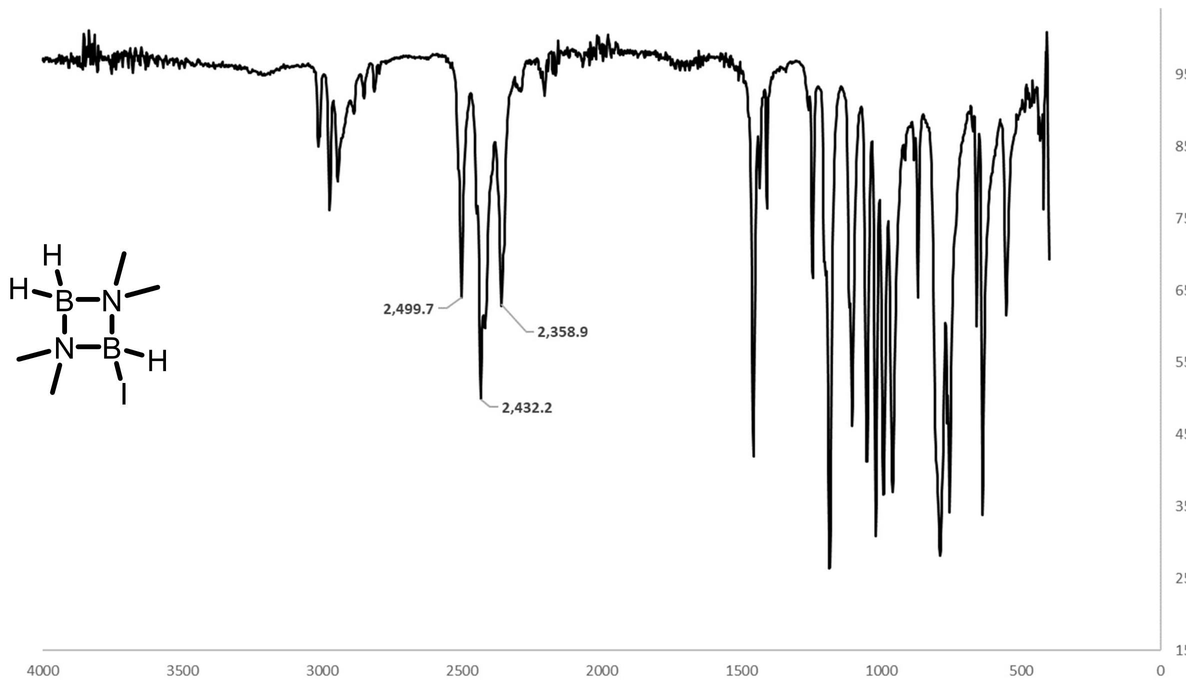

Figure S36: FTIR spectrum of $H_2B(\mu\text{-}Me_2N)_2BH(I)$ **3**.

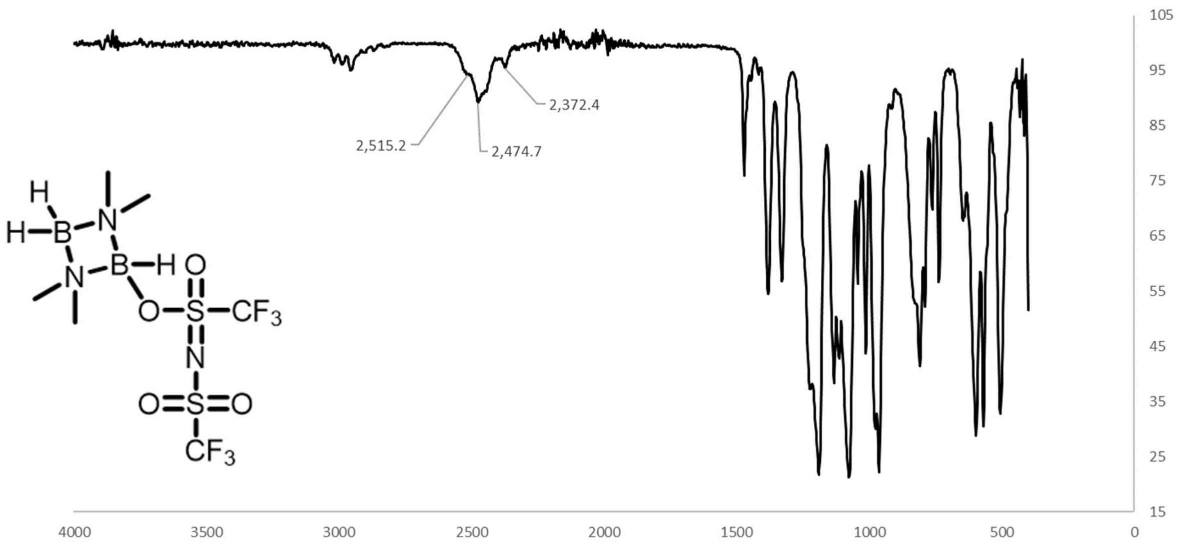

Figure S37: FTIR spectrum of $H_2B(\mu\text{-}Me_2N)_2BH(OSOCF_3NTf)$ **4**.

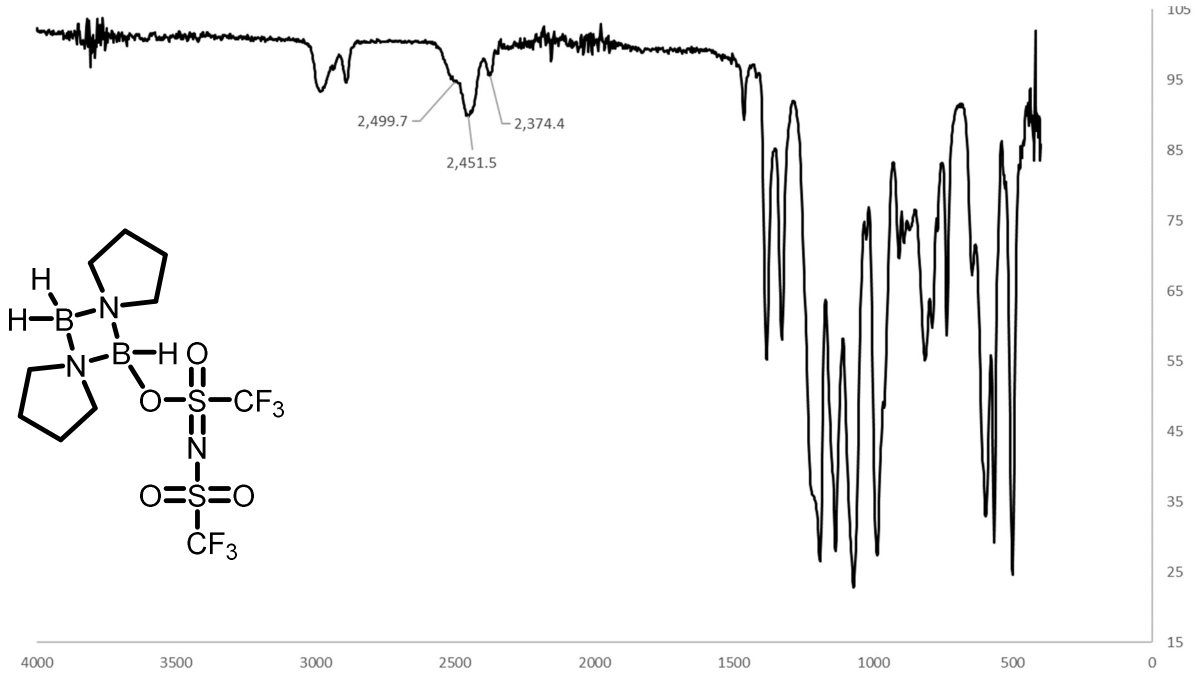

Figure S38: FTIR spectrum of H_2B(μ-pyrrolidine)$_2$BH($OSOCF_3$NTf) **5**.

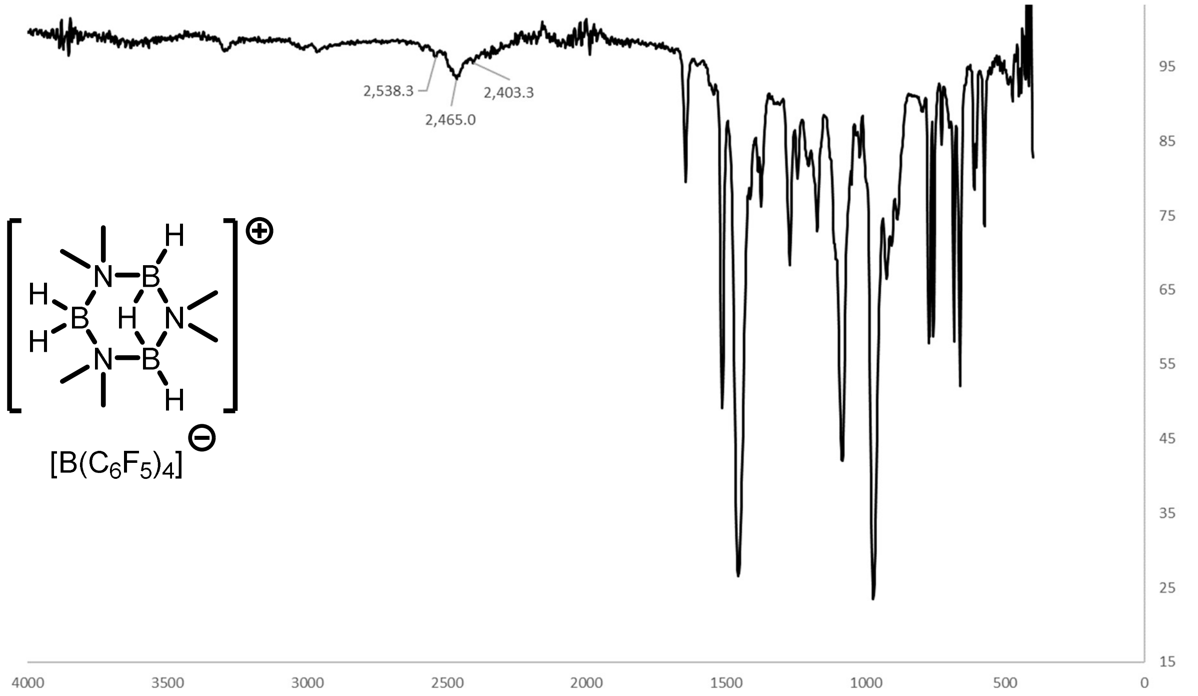

Figure S39: FTIR spectrum of $[(Me_2N)_3B_3H_5][B(C_6F_5)_4]$ **7**.

3.11 **NMR spectra of 8**

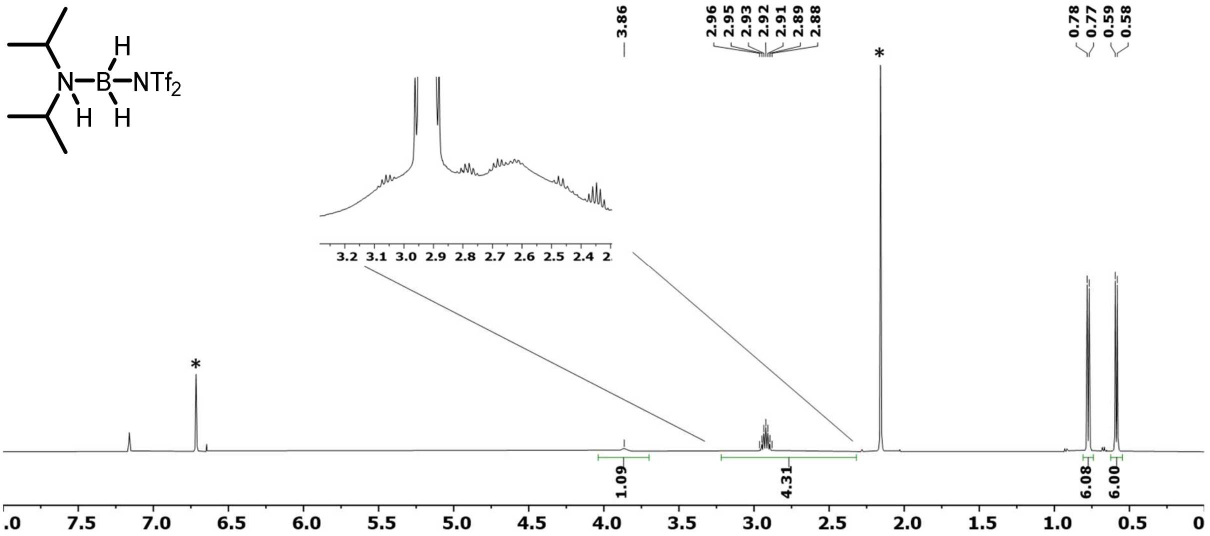

Figure S40: ^{1}H NMR spectrum (500 MHz, C_6D_6, 300 K) of **8** generated *in situ* by the reaction between $(^{i}Pr)_2NBH_2$ and $HNTf_2$. * Signals from Mesitylene (added as an internal standard).

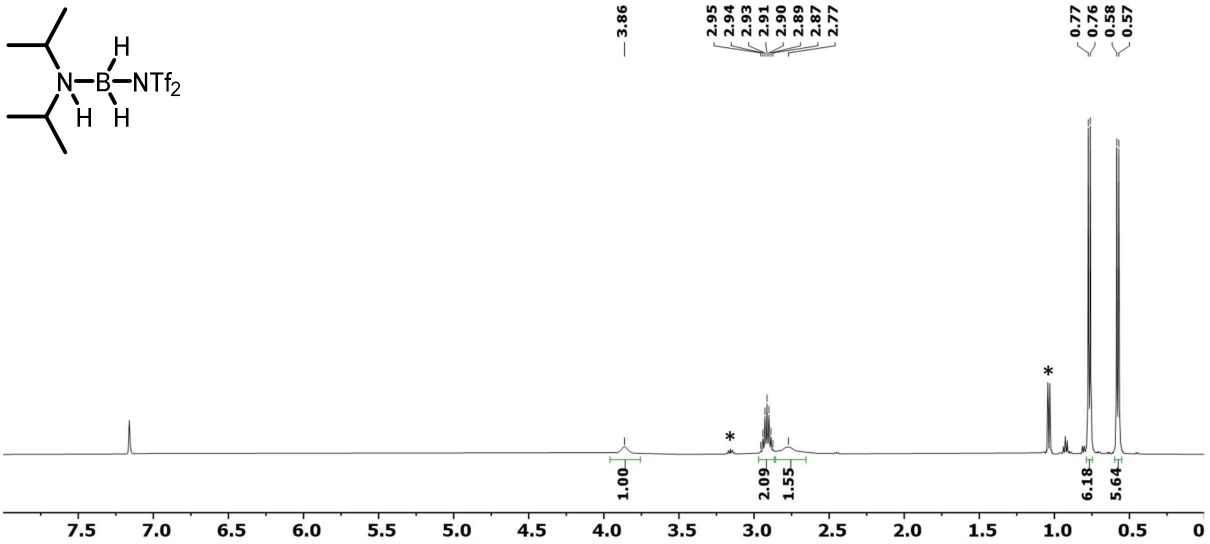

Figure S41: ^{1}H $\{^{11}B\}$ NMR spectrum (500 MHz, C_6D_6, 300 K) of **8** generated *in situ* by the reaction between $(^{i}Pr)_2NBH_2$ and $HNTf_2$. * Signals from trace of unreacted $(^{i}Pr)_2NBH_2$.

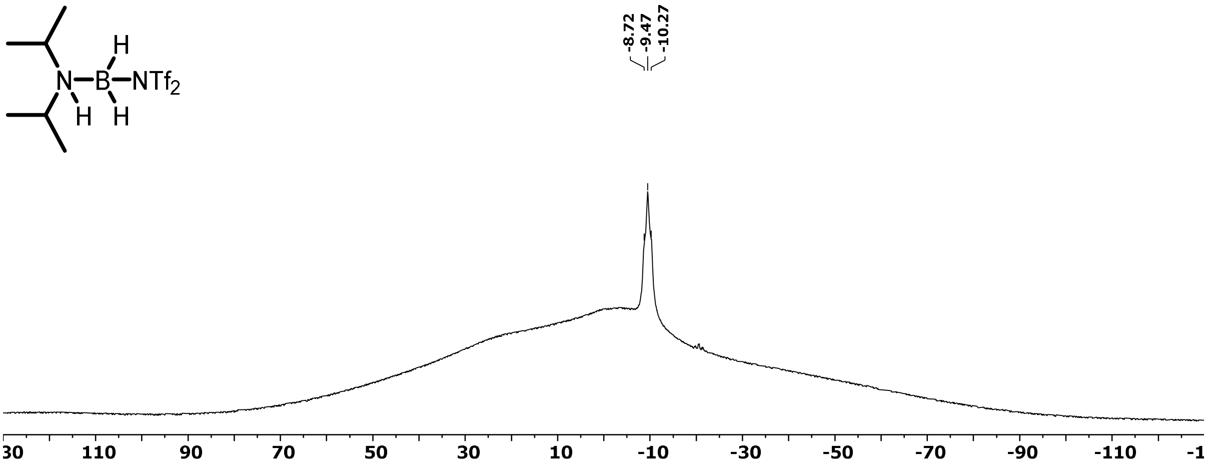

Figure S42: ^{11}B NMR spectrum (160 MHz, C_6D_6, 300 K) of **8** generated *in situ* by the reaction between $(^iPr)_2NBH_2$ and $HNTf_2$.

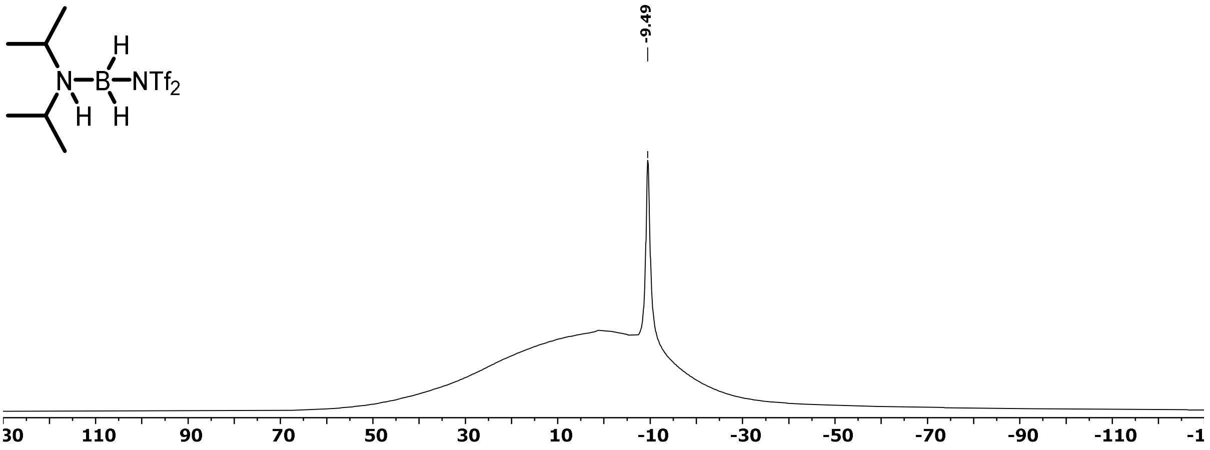

Figure S43: ^{11}B {^1H} NMR spectrum (160 MHz, C_6D_6, 300 K) of **8** generated *in situ* by the reaction between $(^iPr)_2NBH_2$ and $HNTf_2$.

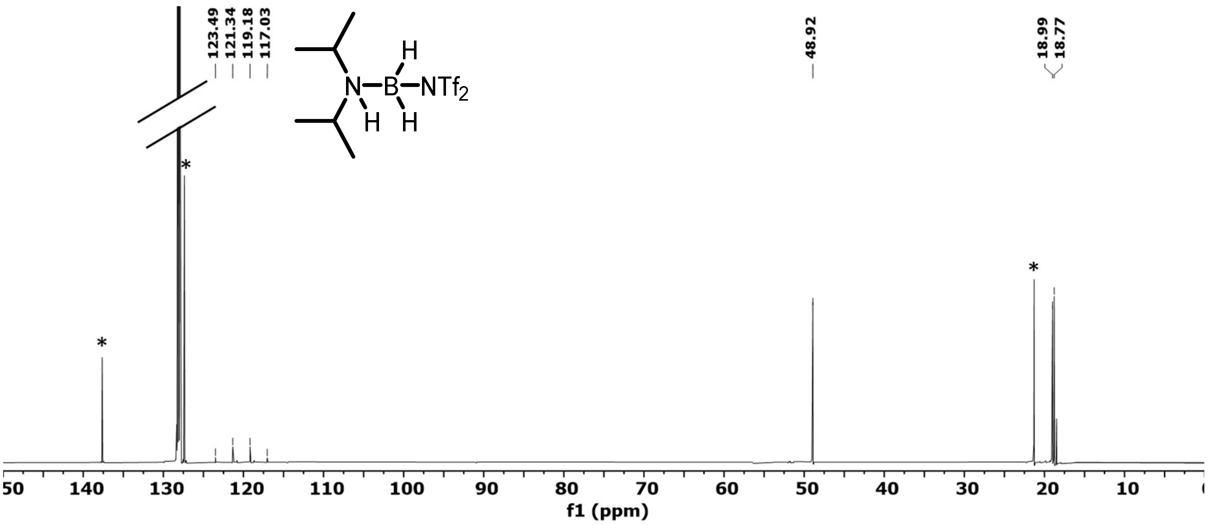

Figure S44: $^{13}C\{^{1}H\}$ NMR spectrum (151 MHz, C_6D_6, 300 K) of **8** generated *in situ* by the reaction between $(^{i}Pr)_2NBH_2$ and $HNTf_2$. * Signals from Mesitylene.

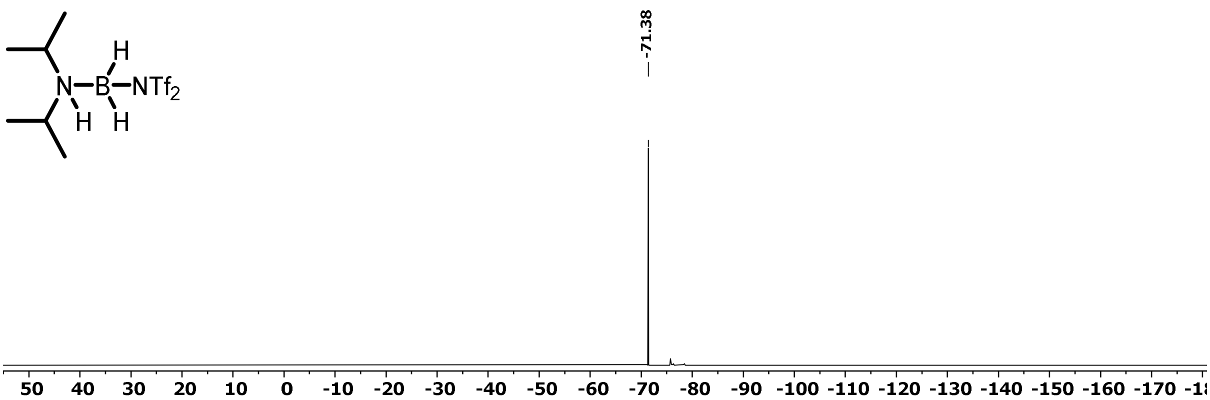

Figure S45: ^{19}F NMR spectrum (471 MHz, C_6D_6, 300 K) of **8** generated *in situ* by the reaction between $(^{i}Pr)_2NBH_2$ and $HNTf_2$.

3.12 **NMR spectra of (^{i}Pr)$_2$NBHNTf$_2$ 9**

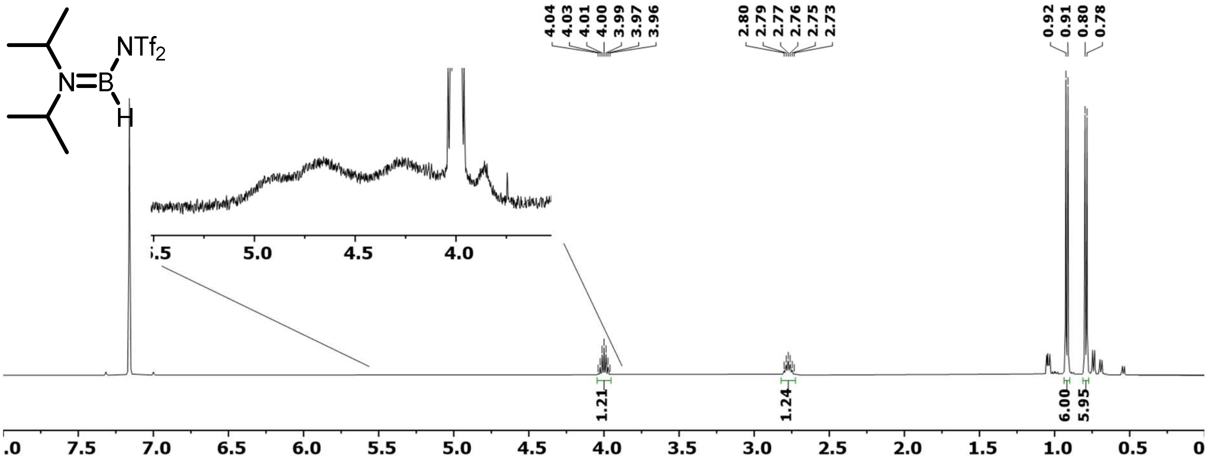

Figure S46: ^{1}H NMR spectrum (500 MHz, C_6D_6, 300 K) of **9**.

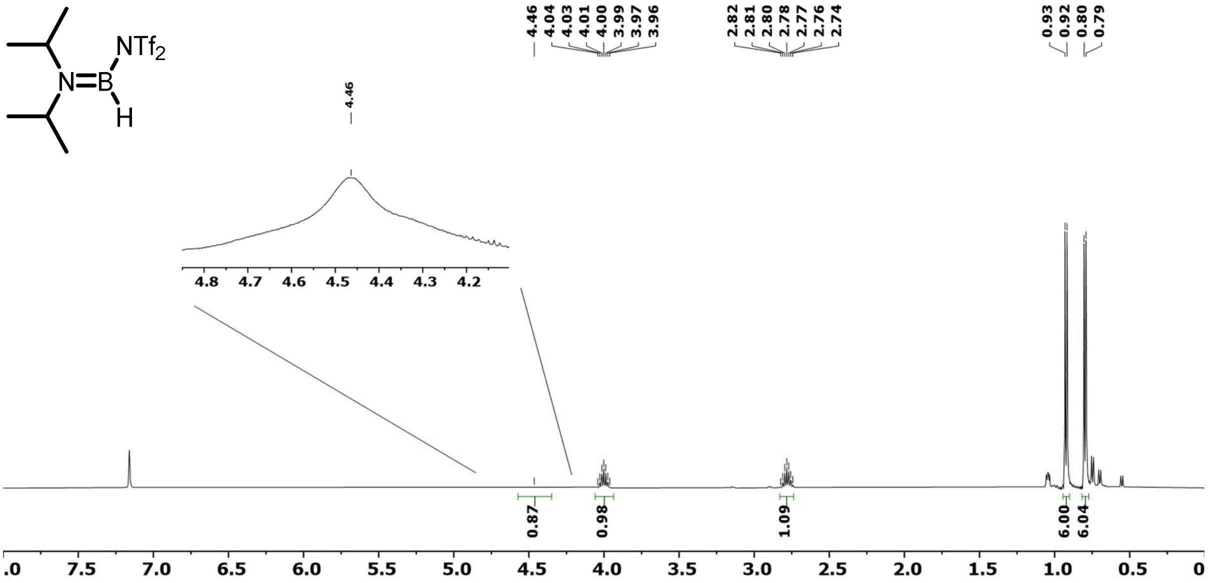

Figure S47: ^{1}H {^{11}B} NMR spectrum (500 MHz, C_6D_6, 300 K) of **9**.

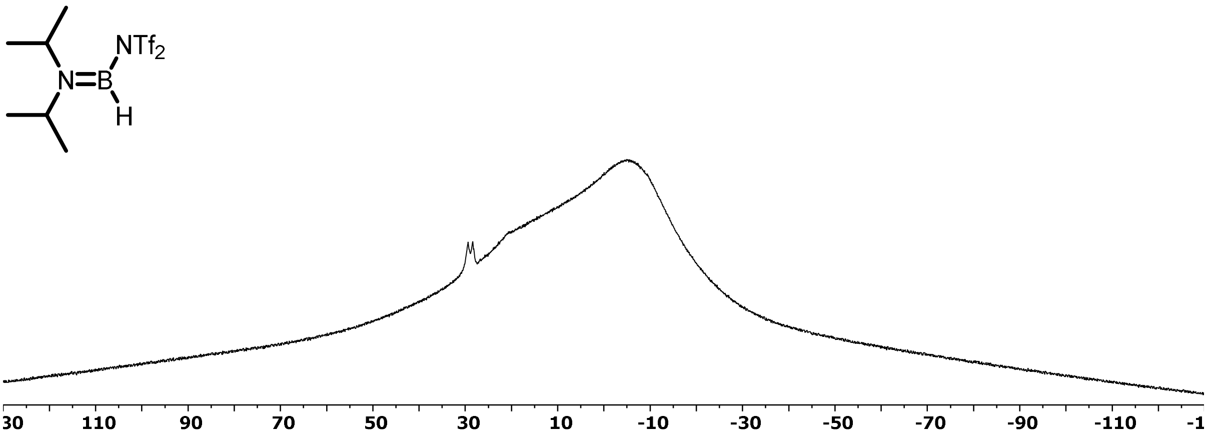

Figure S48: ^{11}B NMR spectrum (160 MHz, C_6D_6, 300 K) of **9**.

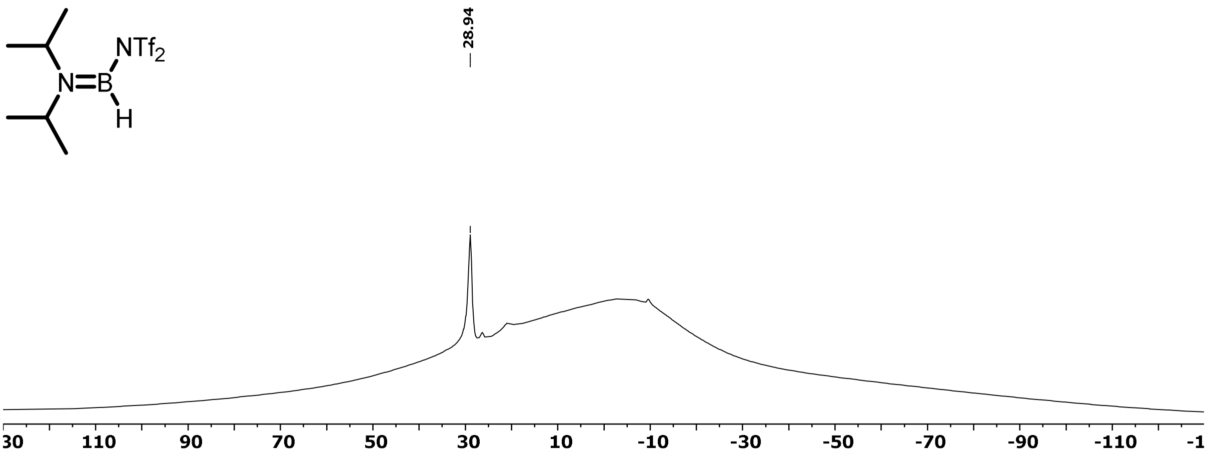

Figure S49: ^{11}B {^{1}H} NMR spectrum (160 MHz, C_6D_6, 300 K) of **9**.

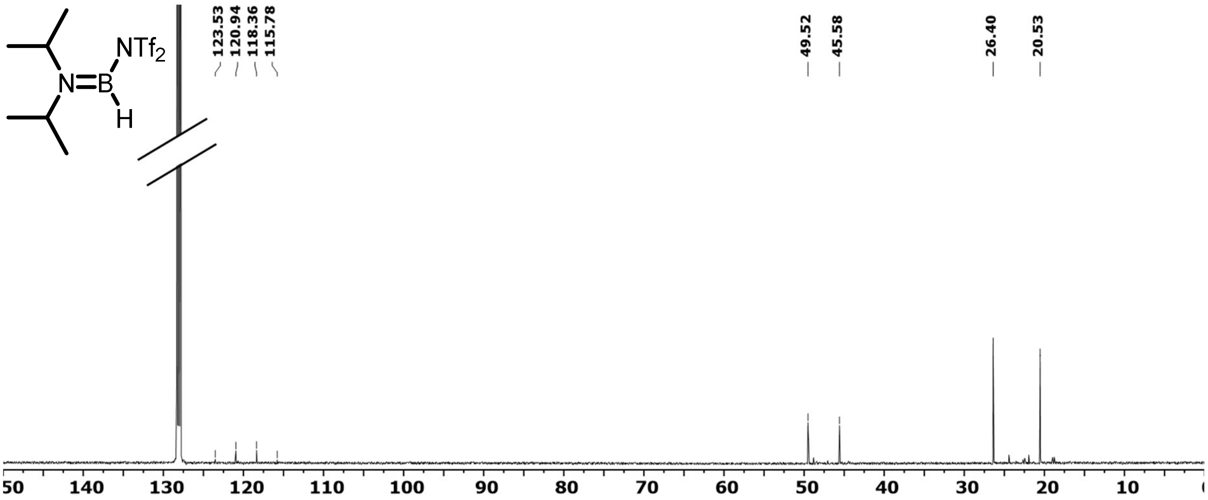

Figure S50: ^{13}C {^{1}H} NMR spectrum (126 MHz, C_6D_6 300 K) of **9**.

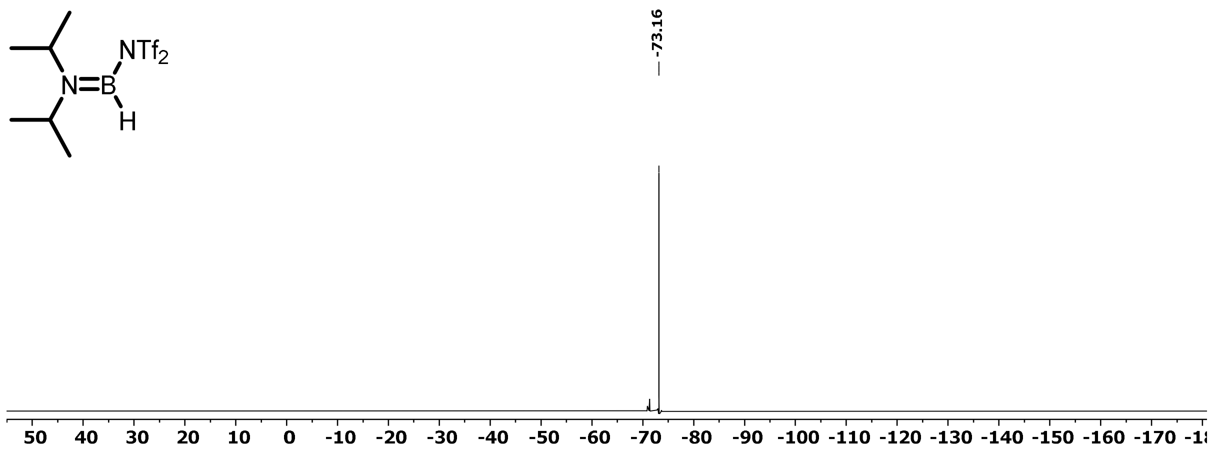

Figure S51: ^{19}F NMR spectrum (471 MHz, C_6D_6, 300 K) of **9.**

4 Hydroboration of alkynes

4.1 Hydroboration of diphenylacetylene with 3

4.1.1 Internal conversion to 10

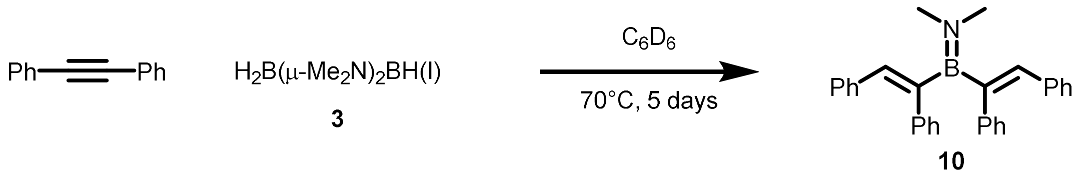

$H_2B(\mu\text{-}Me_2N)_2BH(I)$ **3** (10 mg, 0.04 mmol, 1.00 equiv.), diphenylacetylene (15 mg, 0.08 mmol, 2 equiv.) and mesitylene (internal standard) were dissolved in C_6D_6 (0.5 mL) in an NMR tube and heated at 70 °C for 5 days. The *in situ* conversion to the product **10** was measured to be ca. 5% (conversion was determined by integration of the ^1H NMR relative to internal standard).

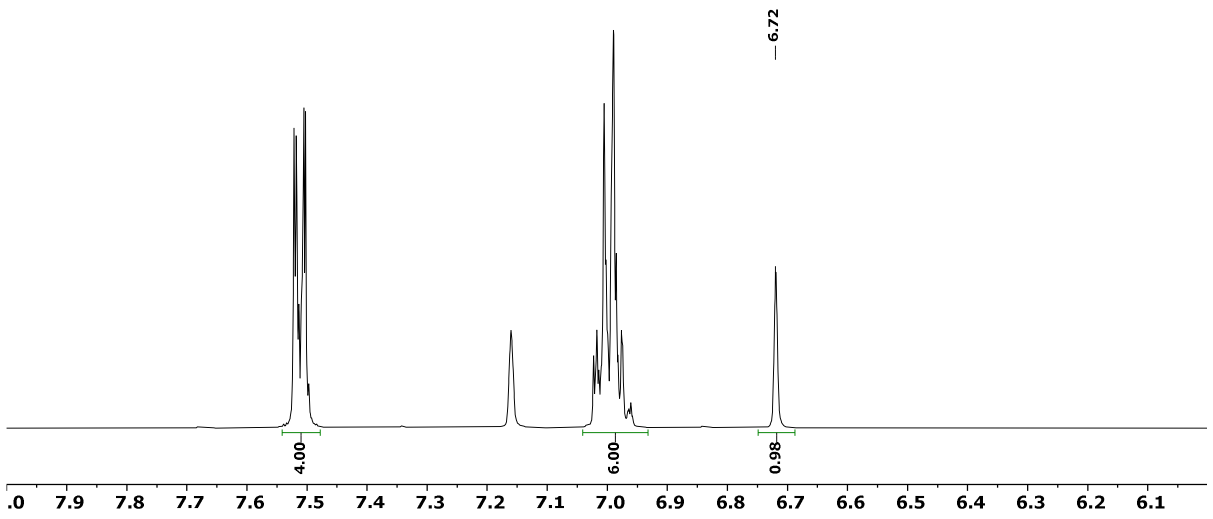

Figure S52: ^1H NMR spectrum (500 MHz, C_6D_6, 300 K) focusing on the aromatic region of the reaction between diphenylacetylene and **3**, with mesitylene as internal standard, before heating. The ratio diphenylacetylene:mesitylene (3.05:1) was calculated by comparison of the integration values between the diphenylacetylene peaks and the aromatic C*H* peak from mesitylene at 6.72 ppm.

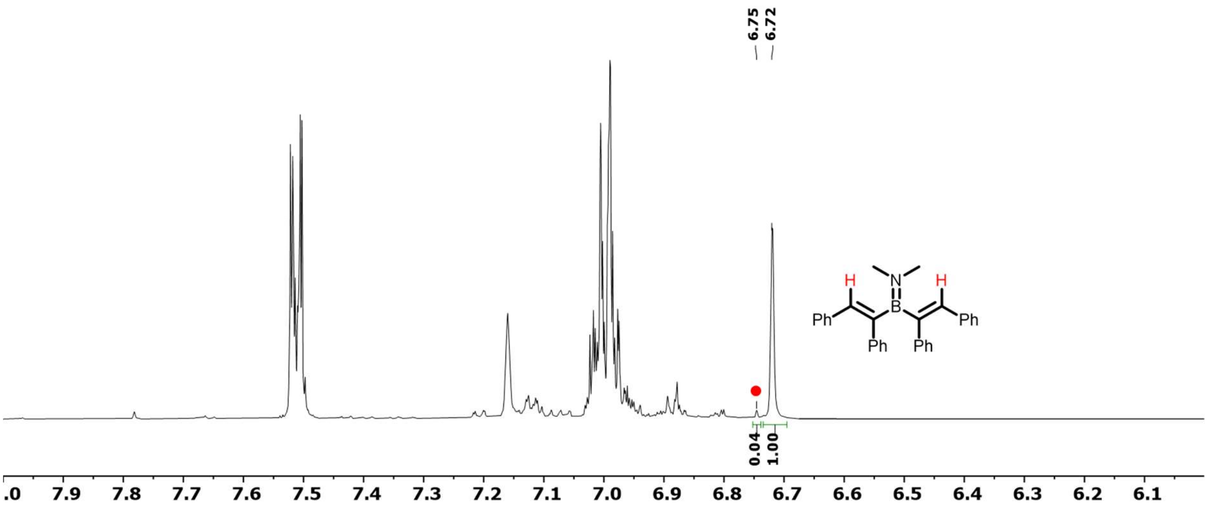

Figure S53: ^{1}H NMR spectrum (500 MHz, C_6D_6, 300 K) focusing on the aromatic region of the reaction between diphenylacetylene and **3**, with mesitylene as internal standard, after 5 days at 70 °C. The ratio of **10**:mesitylene (0.04:1) was calculated by comparison of the integration values between the alkene protons peak from **10** (labelled in red on spectrum) and the aromatic C*H* peak from mesitylene at 6.72 ppm.

4.2 **Hydroboration of diphenylacetylene with 4**

4.2.1 ***Internal conversion to 10***

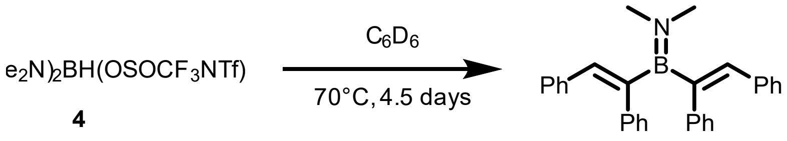

10

$H_2B(\mu\text{-}Me_2N)_2BH(OSOCF_3NTf)$ **4** (25 mg, 0.06 mmol, 1.00 equiv.), diphenylacetylene (23 mg, 0.12 mmol, 2.00 equiv.) and mesitylene (internal standard) were dissolved in C_6D_6 (0.5 mL) in an NMR tube and heated at 70 °C for 4.5 days. The *in situ* conversion to the product **10** was measured to be 95% (conversion was determined by integration of the ^{1}H NMR spectrum relative to internal standard).

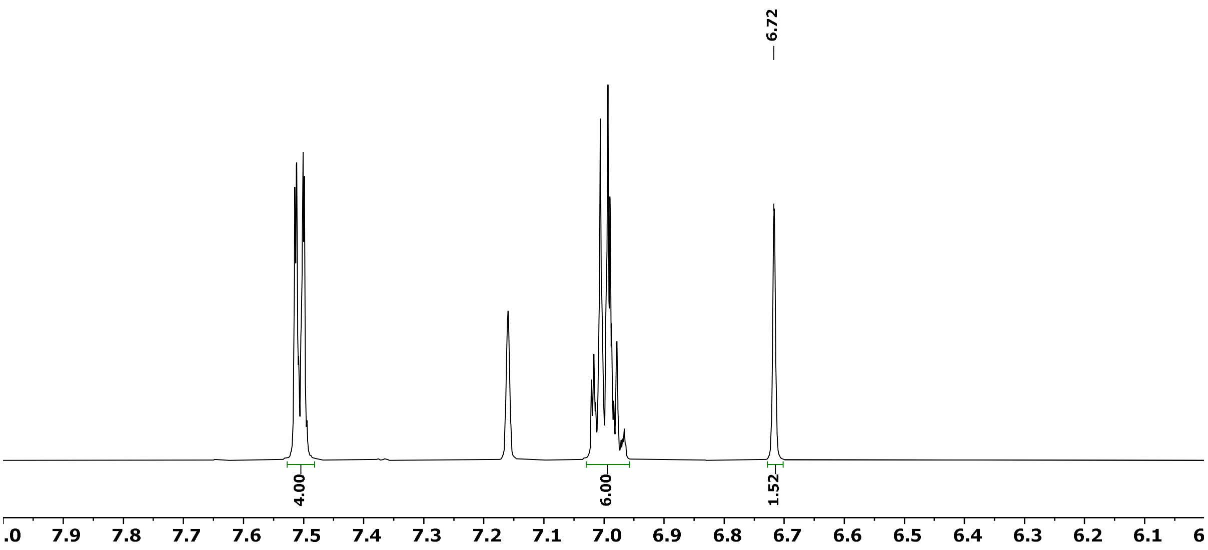

Figure S54: ^{1}H NMR spectrum (500 MHz, C_6D_6, 300 K) focussing on the aromatic region of the reaction between diphenylacetylene and **4**, with mesitylene as internal standard, before heating. The ratio diphenylacetylene:mesitylene (1.97:1) was calculated by comparison of the integration values between the diphenylacetylene peaks and the aromatic C*H* peak from mesitylene at 6.72 ppm.

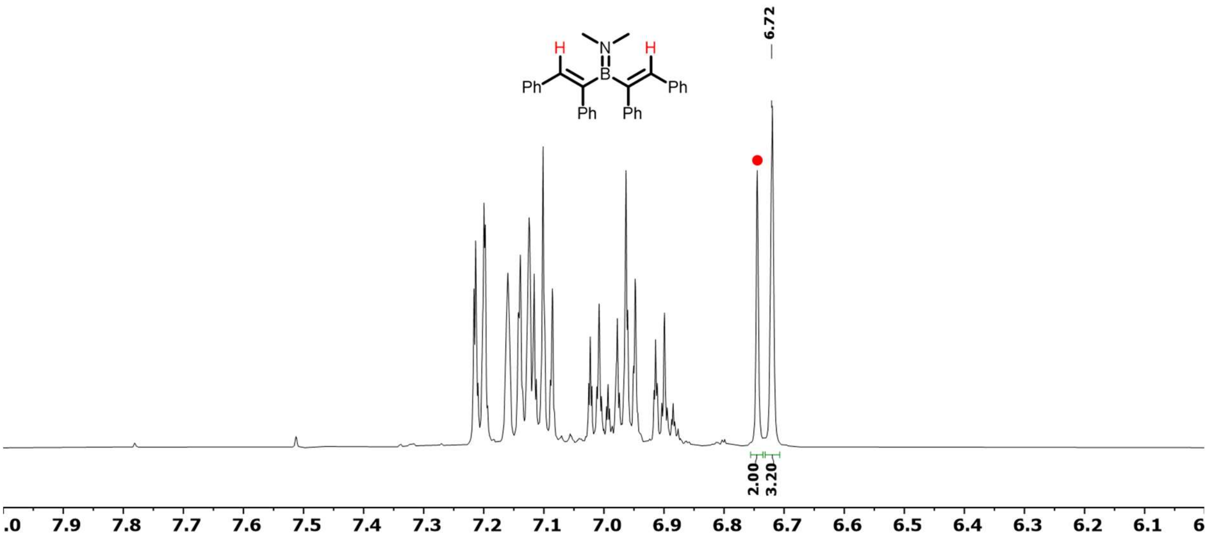

Figure S55: ^{1}H NMR spectrum (500 MHz, C_6D_6, 300 K) focussing on the aromatic region of the reaction between diphenylacetylene and **4**, with mesitylene as internal standard, after 4.5 days at 70 °C. The ratio **10**:mesitylene (0.94:1) was calculated by comparison of the integration values between the alkene protons peak from **10** (labelled in red on spectrum) and the aromatic C*H* peak from mesitylene at 6.72 ppm.

4.2.2 *Characterisation of 10*

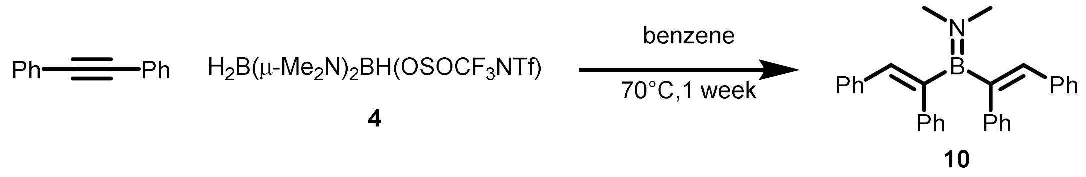

A solution of $H_2B(\mu\text{-}Me_2N)_2BH(OSOCF_3NTf)$ **4** (0.22 g, 0.56 mmol, 1.00 equiv.) in benzene (1 mL) was added to a solution of diphenylacetylene (0.20 g, 1.12 mmol, 2.00 equiv.) in benzene (1 mL). The resulting solution was heated at 70 °C for 1 week. While heating, the solution turned slowly from colourless to dark orange. The volatiles were removed under vacuum, affording an oil. The oil was extracted with pentane (2 mL) giving a clear orange solution. The solution was cooled down to –35 °C and filtered at this temperature. Removal of volatiles *in vacuo* afforded the product **10** as an orange oil (0.11 g), contaminated with remaining trace of $-NTf_2$ side-products.

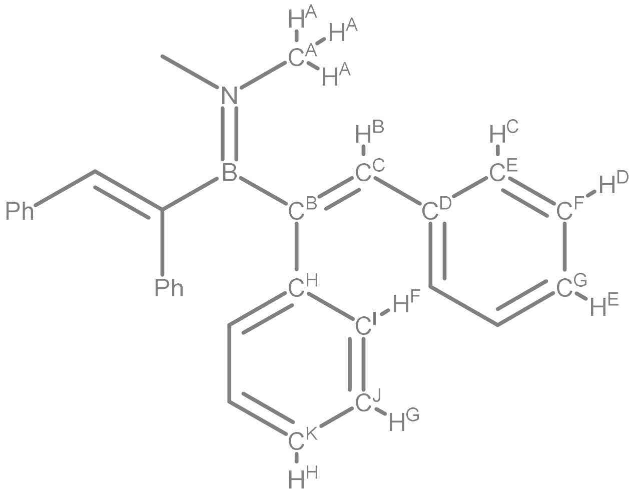

Figure S56: Scheme of **10**.

^{1}H NMR (500 MHz, C_6D_6, 300 K) δ 7.23-7.19 (m, 4H, H^F), 7.15-7.12 (m, 4H, H^C), 7.12-7.08 (m, 4H, H^G), 7.03-6.99 (m, 2H, H^H), 6.99-6.94 (m, 4H, H^D), 6.92-6.88 (m, 2H, H^E), 6.75 (s, 2H, H^B), 2.68 (s, 6H, H^A).

^{11}B NMR (160 MHz, C_6D_6, 300 K) δ 40.0 (br, s, *B*N(Me)$_2$).

^{13}C {^{1}H} NMR (126 MHz, C_6D_6, 300 K) δ 147.43 (br, s, C^B), 142.95 (s, C^H), 138.50 (s, C^D), 134.72 (s, C^C), 129.77 (s, C^E), 129.03 (s, C^I), 128.82 (s, C^J), 128.27 (s, C^F)^a, 126.90 (s, C^G), 126.29 (s, C^K), 40.99 (s, C^A).

^{a} The carbon signal of C^F was hidden by the solvent peaks, its assignment was done using a HSQC experiment.

Mass Spectrum: HRMS (ESI+) m/z calculated for $C_{30}H_{28}BN$: 413.23093; Found: 413.23142.

4.2.3 NMR Spectra of 10

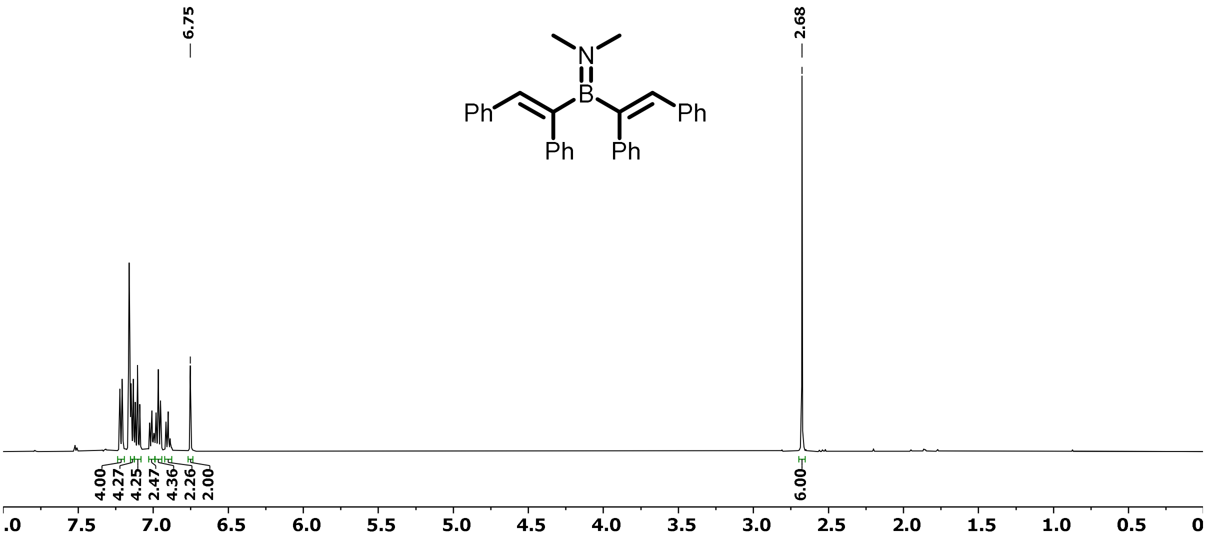

Figure S57: ^{1}H NMR spectrum (500 MHz, C_6D_6, 300 K) of **10**.

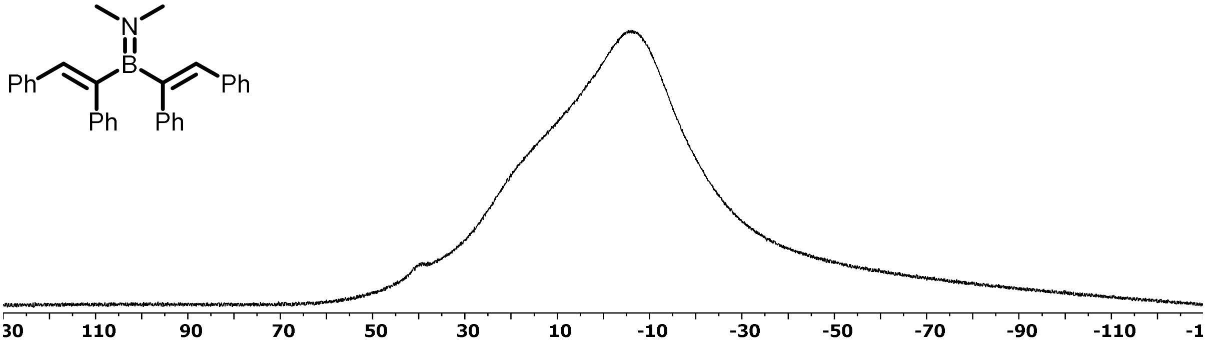

Figure S58: ^{11}B NMR spectrum (160 MHz, C_6D_6, 300 K) of **10**.

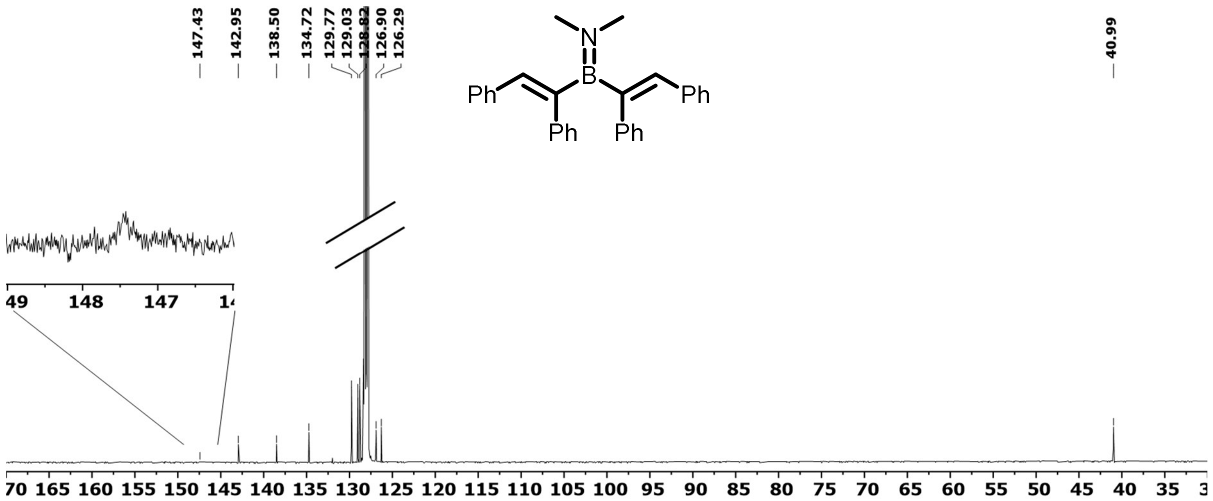

Figure S59: ^{13}C NMR spectrum (126 MHz, C_6D_6, 300 K) of **10**.

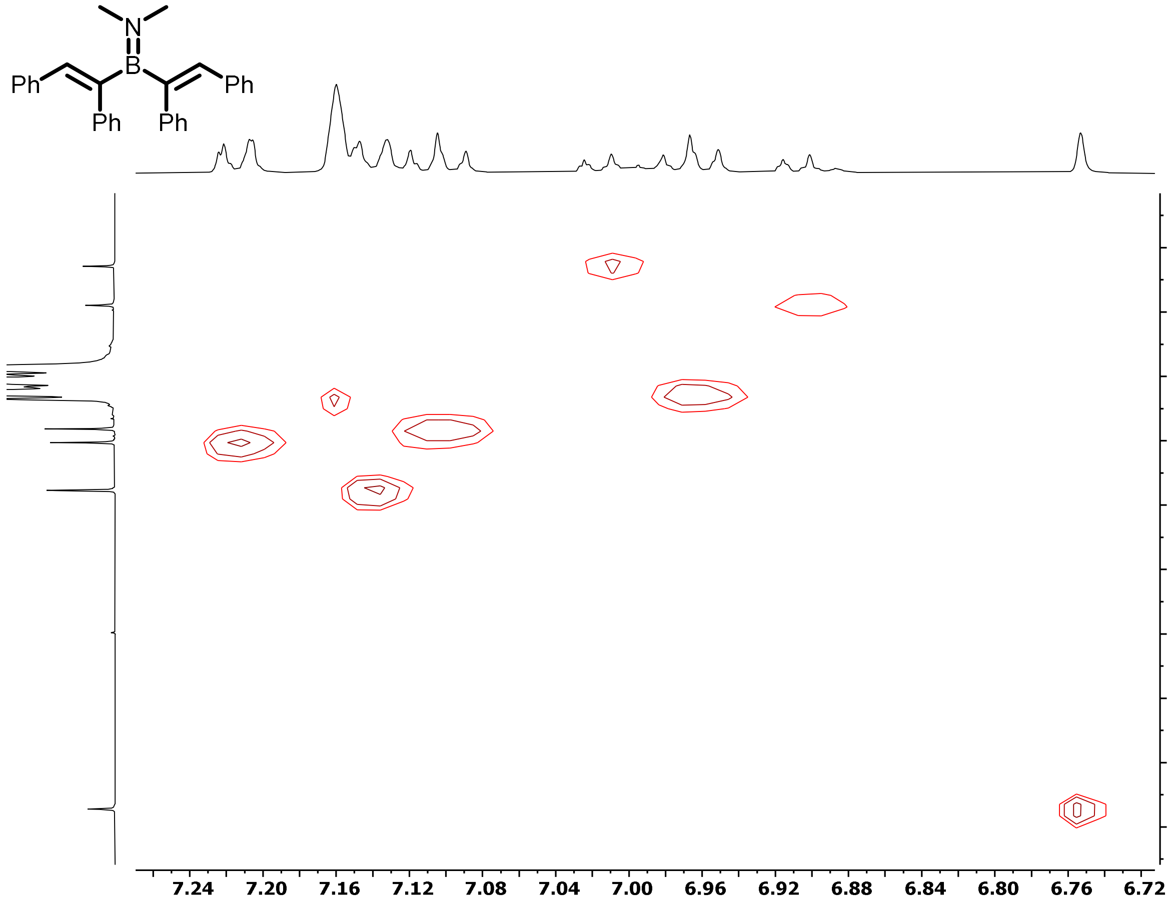

Figure S60: 2-D ^{13}C–^{1}H HSQC NMR spectrum (126–500 MHz, C_6D_6, 300 K) focussed on the aromatic region of **10**.

4.3 Hydroboration of 1-phenyl-1-propyne with 4

4.3.1 Internal conversion to 12a-c

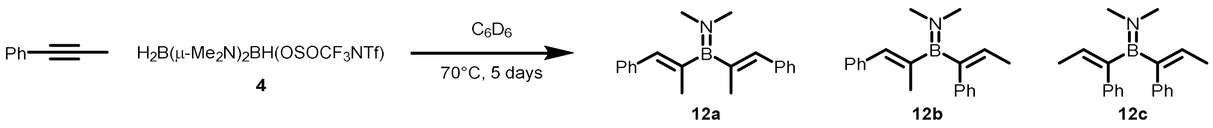

$H_2B(\mu\text{-}Me_2N)_2BH(OSOCF_3NTf)$ **4** (25 mg, 0.06 mmol, 1.00 equiv.), 1-phenyl-1-propyne (16 µL, 0.12 mmol, 2.00 equiv.) and mesitylene (internal standard) were dissolved in C_6D_6 (0.5 mL) in an NMR tube and heated at 70 °C for 5 days. The *in situ* conversion to the product **12a-c** was measured to be 81% (conversion was determined by integration of the ^1H NMR relative to internal standard), with a ratio of 29:54:17% (**12a:12b:12c**).

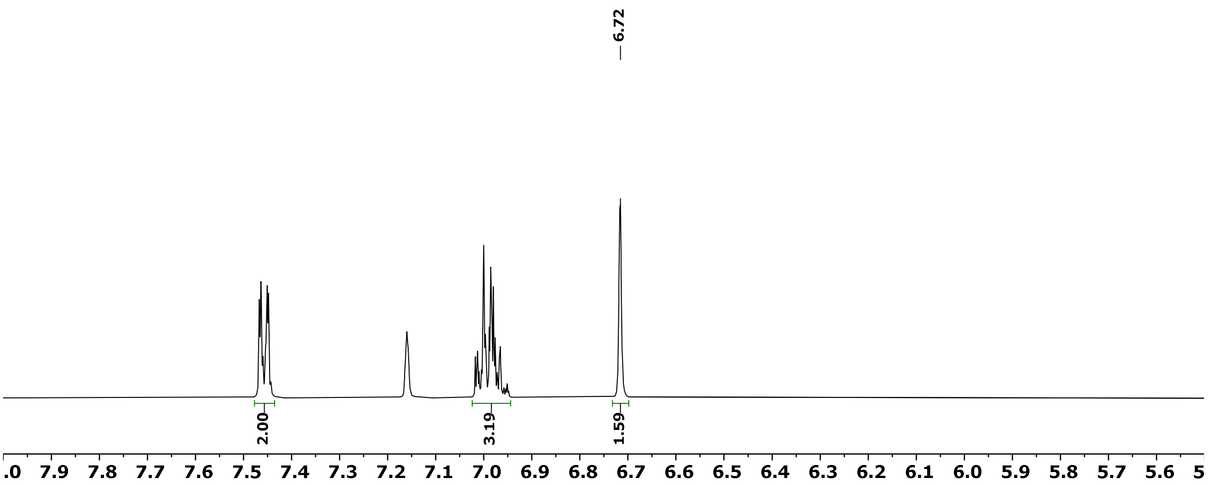

Figure S61: ^1H NMR spectrum (500 MHz, C_6D_6, 300 K) focussing on the aromatic region of the reaction between 1-phenyl-1-propyne and **4**, with mesitylene as internal standard, before heating. The ratio 1-phenyl-1-propyne:mesitylene (1.89:1) was calculated by comparison of the integration values between the 1-phenyl-propyne multiplet at 7.5-7.4 ppm and the aromatic C*H* peak from mesitylene at 6.72 ppm.

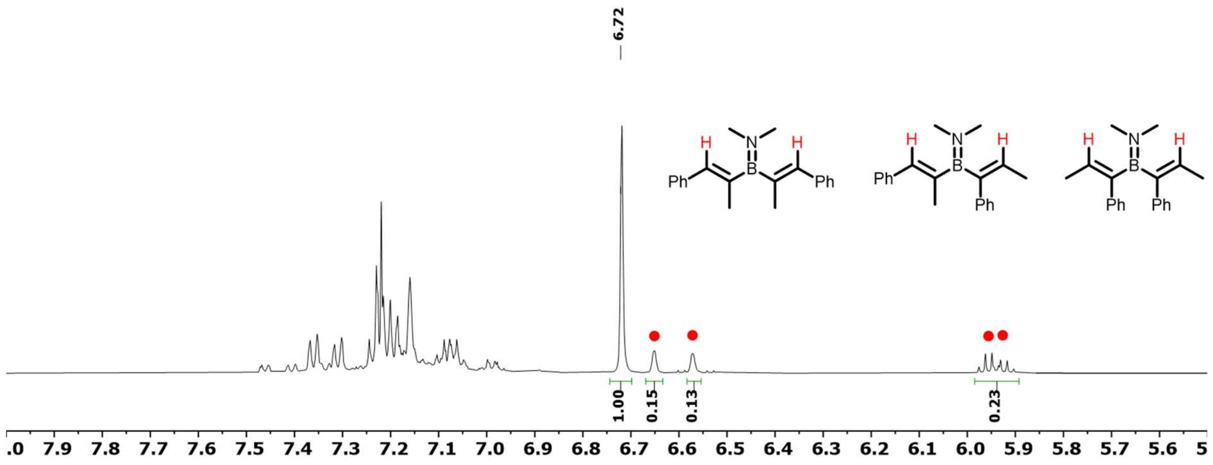

Figure S62: ^{1}H NMR spectrum (500 MHz, C_6D_6, 300 K) focussing on the aromatic region of the reaction between 1-phenyl-1-propyne and **4**, with mesitylene as internal standard, after 5 days at 70 °C. The ratio **12a-c**:mesitylene (0.77:1) was calculated by comparison of the integration values between the alkene protons peaks from **12a-c** (labelled in red on spectrum) and the aromatic C*H* peak from mesitylene at 6.72 ppm.

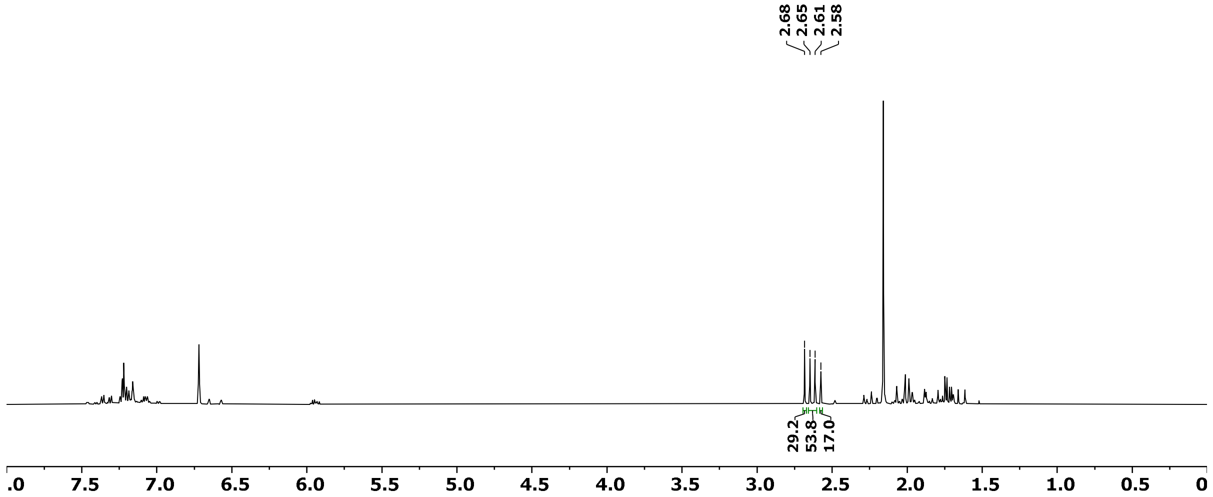

Figure S63: ^{1}H NMR spectrum (500 MHz, C_6D_6, 300 K) of the reaction between 1-phenyl-1-propyne and **4**, with mesitylene as internal standard, after 5 days at 70 °C. Ratio of the different isomers **12a-c** was determined by relative integration of the signal from the $N(CH_3)_2$ moieties.

4.3.2 Characterisation of isomers of 12a-c

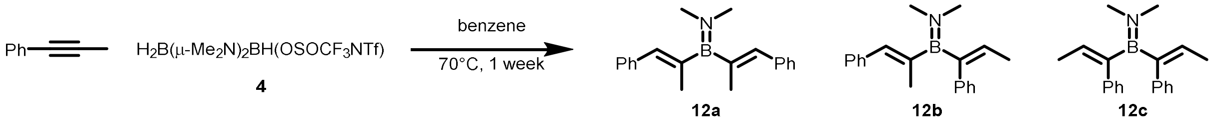

To a solution of $H_2B(\mu\text{-}Me_2N)_2BH(OSOCF_3NTf)$ **4** (0.20 g, 0.50 mmol, 1.00 equiv.) in benzene (2 mL), was added 1-phenyl-1-propyne (130 μL, 1.00 mmol, 2.00 equiv.) using a micro syringe. The resulting solution was heated at 70 °C for 1 week. Volatiles were removed under vacuum, affording an oil. The oil was extracted with pentane (2 mL) affording a clear orange solution. The solution was cooled down to –35 °C and filtered at this temperature. Removal of volatiles *in vacuo* afforded the product **12a-c** as an orange oil (0.018 g) contaminated with remaining trace of side-products.

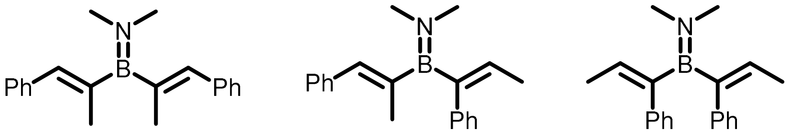

12a **12b** **12c**

^{1}H NMR (400 MHz, C_6D_6, 300 K) **12a**: δ 7.55-6.82 (m, 10H, C_6H_5), 6.66 (d, $^{4}J_{HH}$ = 1.5 Hz, 2H, C=C*H*Ph), 2.68 (s, 6H, N(CH_3)$_2$), 2.02 (d, $^{4}J_{HH}$ = 1.7 Hz, 6H, C=C(BNMe$_2$)CH_3). **12b**: δ 7.55-6.82 (m, 10H, C_6H_5), 6.58 (d, $^{4}J_{HH}$ = 1.2 Hz, 1H, C=C*H*Ph), 5.96 (q, $^{3}J_{HH}$ = 6.7 Hz, 1H, C=C*H*CH$_3$), 2.64 (s, 3H, NCH_3), 2.61 (s, 3H, NCH_3), 2.00 (d, $^{4}J_{HH}$ = 1.7 Hz, 3H, C=C(BNMe$_2$)CH_3), 1.74 (d, $^{3}J_{HH}$ = 6.7 Hz, 3H, C=CHCH_3). **12c**: δ 7.55-6.82 (m, 10H, C_6H_5), 5.93 (q, $^{3}J_{HH}$ = 6.8 Hz, 2H, C=C*H*CH$_3$), 2.57 (s, 6H, N(CH_3)$_2$), 1.72 (d, $^{3}J_{HH}$ = 6.8 Hz, 6H, C=CHCH_3).

^{11}B NMR[a] (160 MHz, C_6D_6, 300 K) 47.1-37.8 (br, *B*N(CH$_3$)$_2$).

^{13}C NMR[b] (126 MHz, C_6D_6, 300 K) **12a**: δ 132.96 (s, C=*C*HPh), 40.58 (s, N(*C*H$_3$)$_2$), 17.36 (s, C=C(BNMe$_2$)*C*H$_3$). **12b**: δ 133.10 (s, C=*C*HPh), 131.50 (s, C=*C*HCH$_3$), 40.88 (s, N*C*H$_3$), 40.70 (s, N*C*H$_3$), 17.58 (s, C=C(BNMe$_2$)*C*H$_3$), 15.69 (s, C=CH*C*H$_3$). **12c**: δ 131.99 (s, C=*C*HCH$_3$), 41.09 (s, N(*C*H$_3$)$_2$), 15.68 (s, C=CH*C*H$_3$).

[a] Each isomer exhibits a boron signal in similar region of the spectrum, it was not possible to assign the boron signals individually to their respective isomer.

[b] The C–B and the aromatic carbons signals could not be assigned, even by 2-D NMR experiments, due to the overlapping of the aromatic proton and aromatic carbon signals of the different isomers and impurities.

Mass Spectrum: HRMS (ESI+) m/z calculated for $C_{20}H_{24}BN$: 289.19963; Found: 289.19920.

4.3.3 NMR Spectra of 12a-c

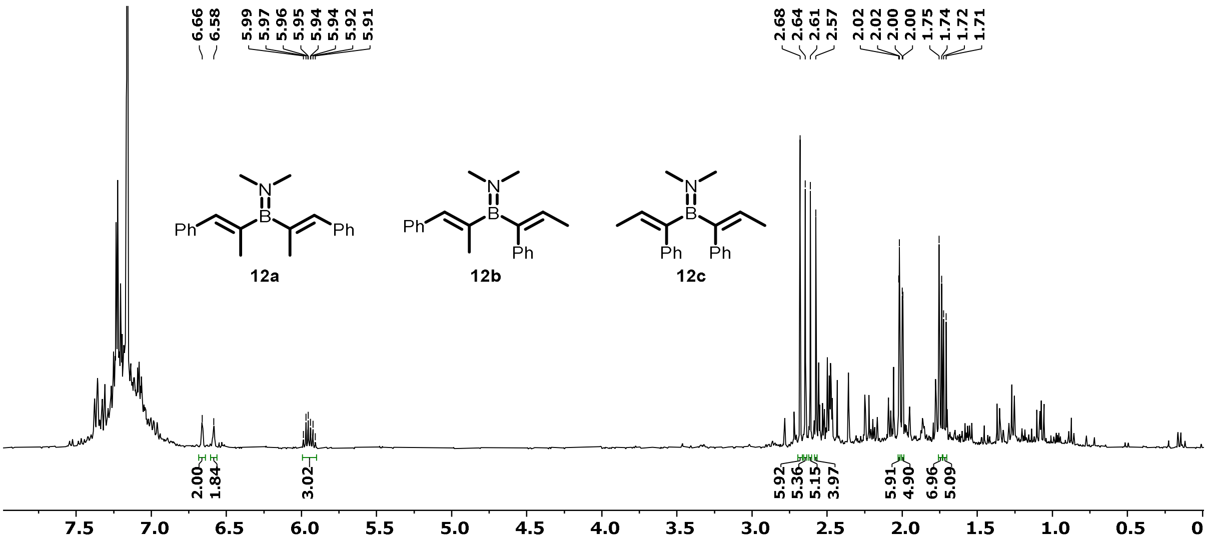

Figure S64: ^{1}H NMR spectrum (400 MHz, C_6D_6, 300 K) of **12a-c**.

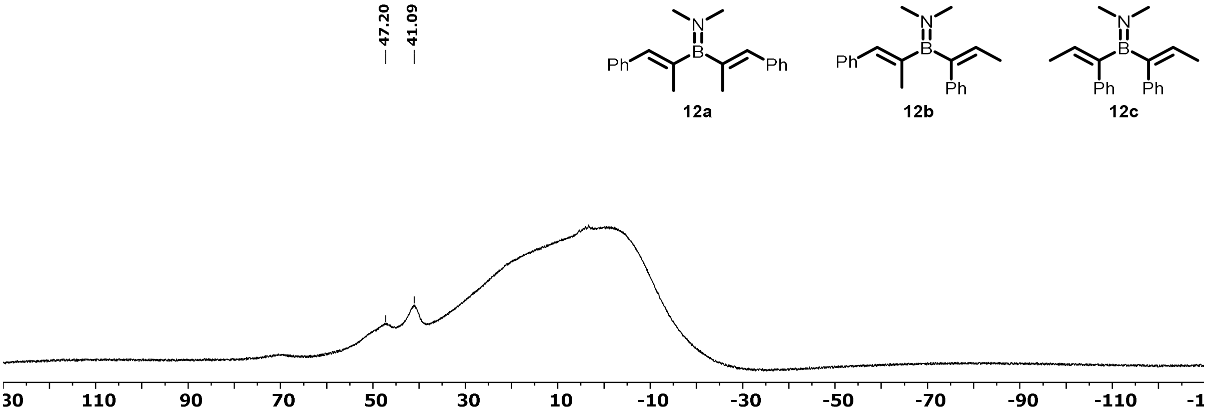

Figure S65: ^{11}B NMR spectrum (160 MHz, C_6D_6, 300 K) of **12a-c**.

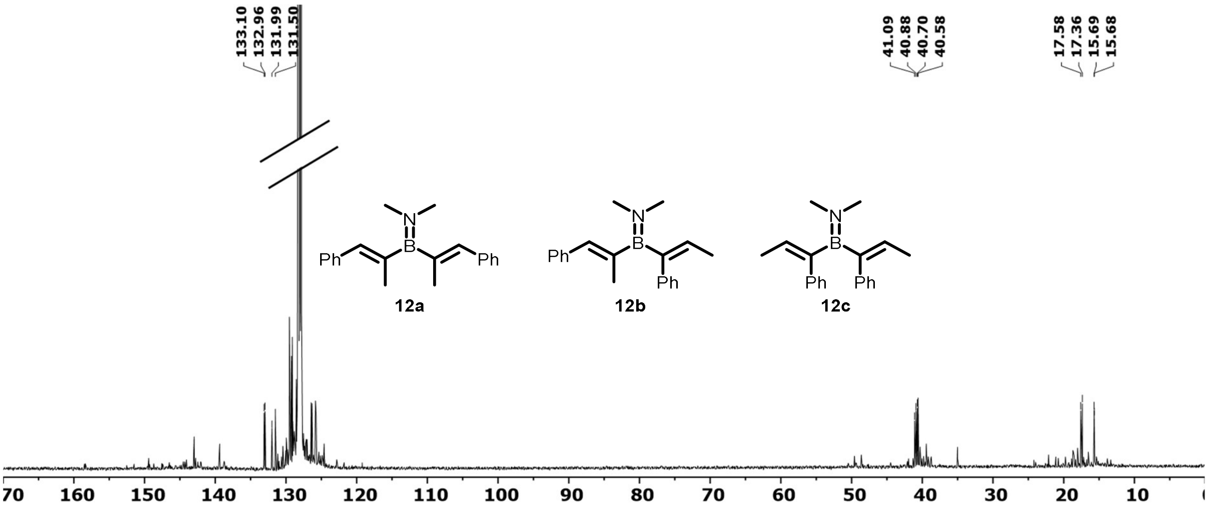

Figure S66: ^{13}C NMR spectrum (126 MHz, C_6D_6, 300 K) of **12a-c**.

5 Complementary studies

5.1 Attempt of synthesis of $[Me_2NBH(I)]_2$

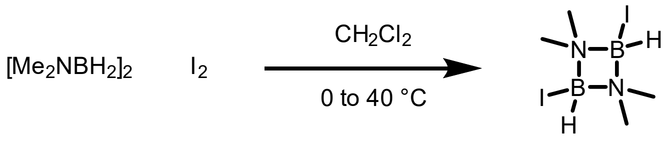

A solution of iodine (0.28 g, 1.00 mmol, 1 equiv.) in CH_2Cl_2 (12 mL) was added dropwise to a solution of $[Me_2NBH_2]_2$ **1** (0.11 g, 1.00 mmol, 1 equiv.) in CH_2Cl_2 (1 mL) at 0 °C. The resulting solution was warmed up to room temperature and stirred for 3 days and finally heated 1 day at 40 °C. The reaction was monitored by NMR spectroscopy.

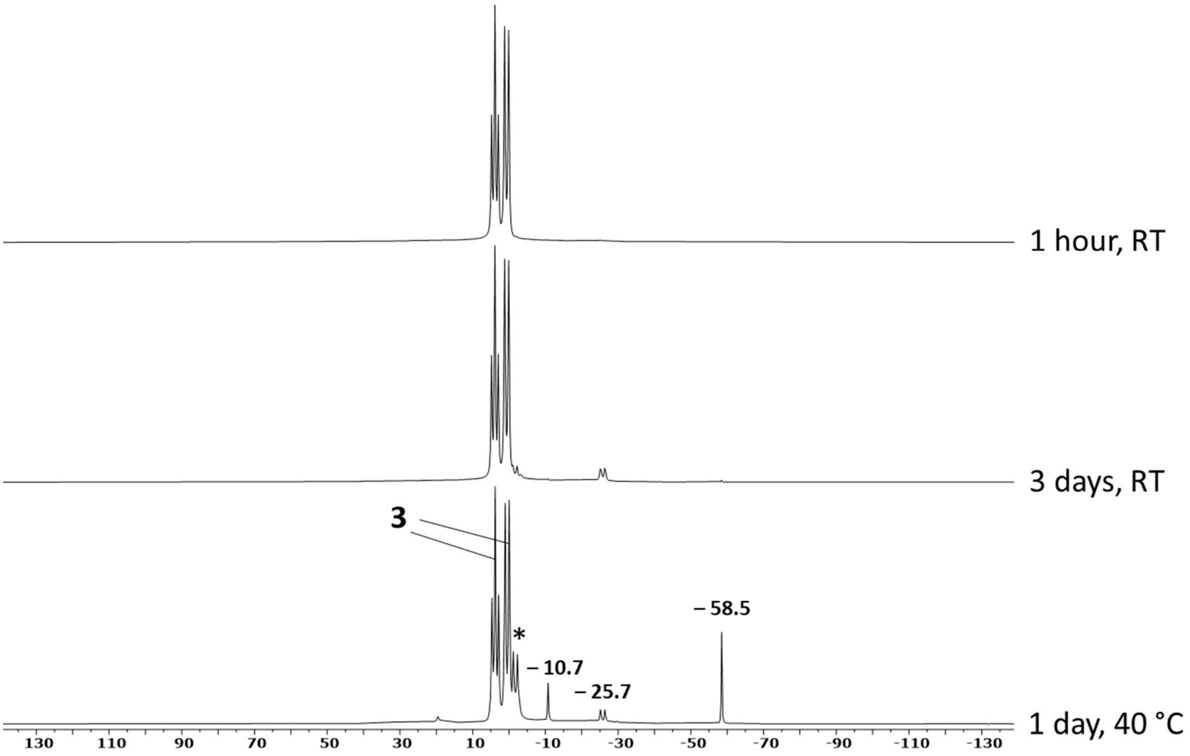

Figure S67: ^{11}B NMR spectrum (128 MHz, CH_2Cl_2, 300 K) of the reaction between **1** and iodine, after 1 hour at room temperature (top), after 3 days at room temperature (middle) and after 1 day at 40 °C. Even with excess iodine, **3** is obtained as the major product. The singlet at δ –58.5 ppm was assigned to be the adduct $Me_2HN\text{-}BI_3$ (by comparison with the reported $Me_3N\text{-}BI_3$ at δ –54 ppm).[S6] The doublet at δ – 25.7 ($^1J_{BH}$ = 158.5 Hz) could not be assigned, we propose that it may be the adduct $Me_2HN\text{-}BHI_2$. The singlet at –10.7 could not be assigned, we suggest that it may be the iodo-analogue of the reported salt $[Cl_2B(HNMe_2)_2]Cl$.[S7] * The doublet at δ –1.73 ppm ($^1J_{BH}$ = 144 Hz) came in a very similar region to the doublet observed for **3** (δ 0.4 ppm, $^1J_{BH}$ = 142 Hz) and exhibits an almost identical coupling constant; we propose that this signal may come from the bis iodide derivative, $[Me_2NBH(I)]_2$. However, the formation of $[Me_2NBH(I)]_2$ was slow and not clean.

5.2 Control reactions

5.2.1 Hydroboration of diphenylacetylene with [Me2NBH2]2 1

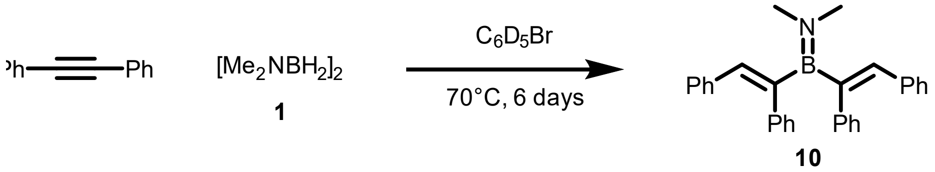

[Me_2NBH_2]$_2$ **1** (10 mg, 0.09 mmol, 1.00 equiv.) and diphenylacetylene (31 mg, 0.18 mmol, 2 equiv.) were dissolved in C_6D_5Br (0.5 mL) in an NMR tube and heated at 70 °C for 6 days.

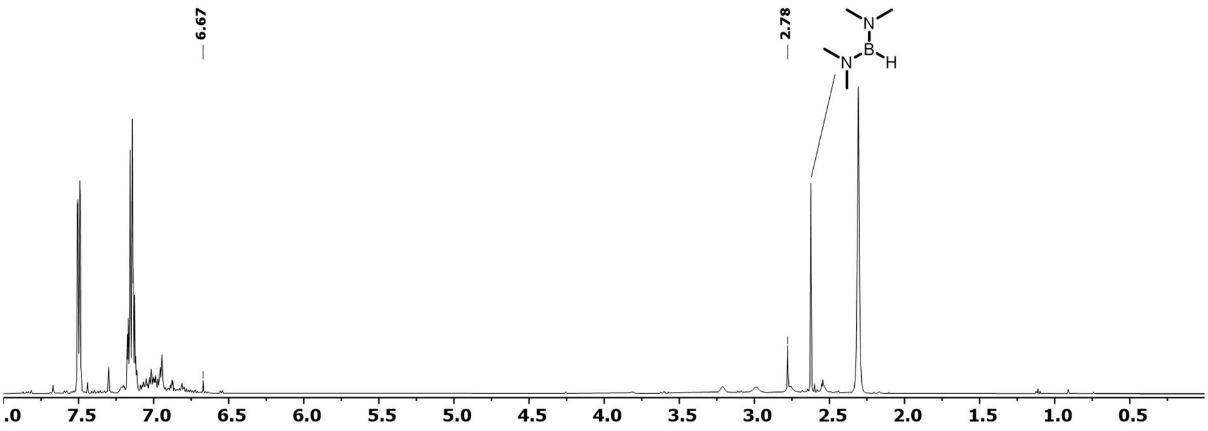

Figure S68: ^{1}H NMR spectrum (500 MHz, C_6D_5Br, 300 K) of the reaction between **1** and diphenylacetylene after 6 days at 70 °C. The diphenylacetylene and **1** remains mostly unreacted. Traces of the product **10** is observed (asserted by the two singlets at δ 6.67 and 2.78) along with $HB(N(CH_3)_2)_2$ forming by decomposition of **1**.

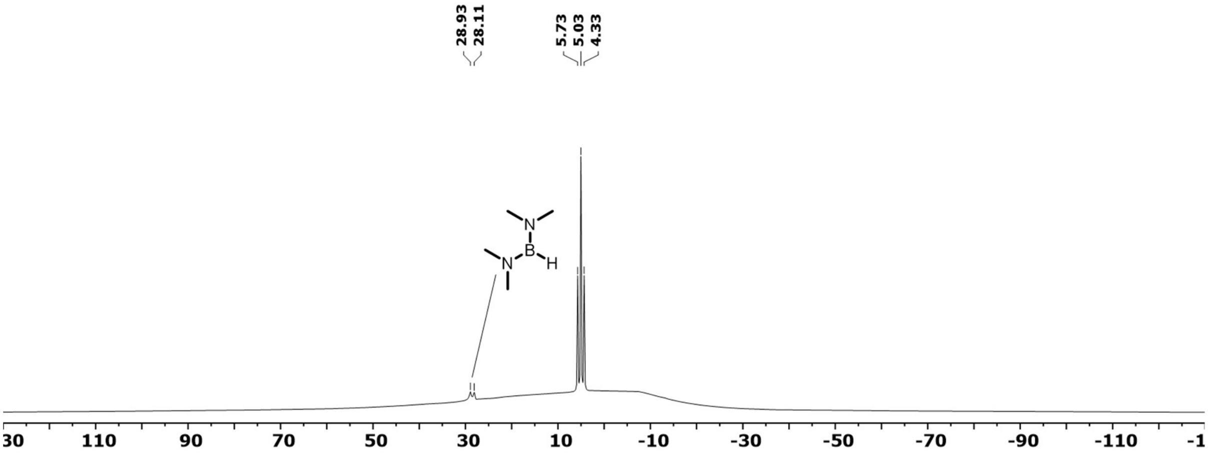

Figure S69: ^{11}B NMR spectrum (160 MHz, C_6D_5Br, 300 K) of the reaction between **1** and diphenylacetylene after 6 days at 70 °C. Only **1** is observed along with trace of its decomposition product $HB(N(CH_3)_2)_2$ (asserted by the doublet at δ 28.5, $^1J_{BH}$ = 130 Hz).

5.2.2 *Hydroboration of diphenylacetylene with [Me2NBH2]2 1 and catalytic amount of 4*

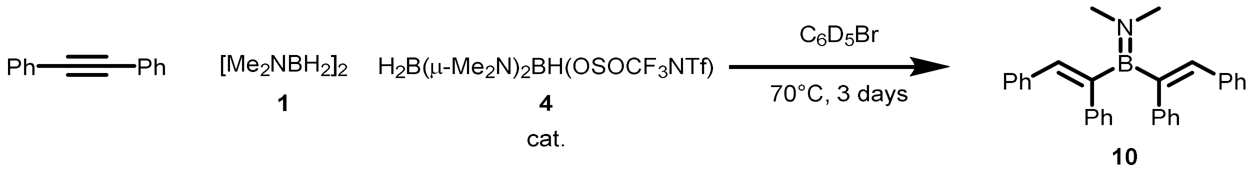

$[Me_2NBH_2]_2$ **1** (10 mg, 0.09 mmol, 0.90 equiv.), Diphenylacetylene (36 mg, 0.20 mmol, 2 equiv.) and $H_2B(\mu\text{-}Me_2N)_2BH(OSOCF_3NTf)$ **4** were dissolved in C_6D_5Br (0.5 mL) in an NMR tube and heated at 70 °C for 3 days.

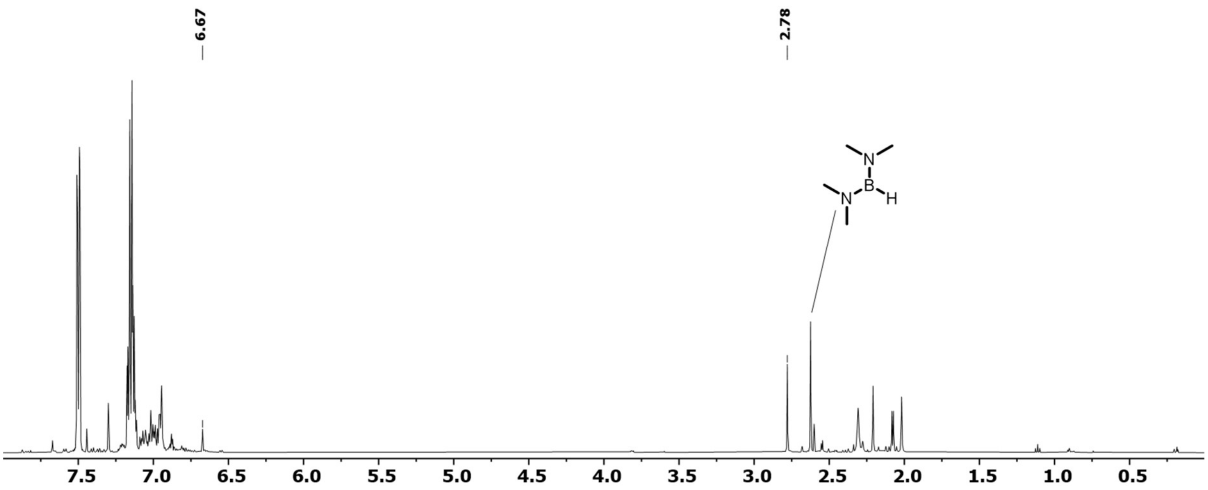

Figure S70: ^{1}H NMR spectrum (500 MHz, C_6D_5Br, 300 K) of the reaction between **1**, **4** and Diphenylacetylene after 3 days at 70 °C. The diphenylacetylene and **1** remains mostly unreacted. Traces of the product **10** is observed (asserted by the two singlets at δ 6.67 and 2.78) along with $HB(N(CH_3)_2)_2$ forming by decomposition of **1**.

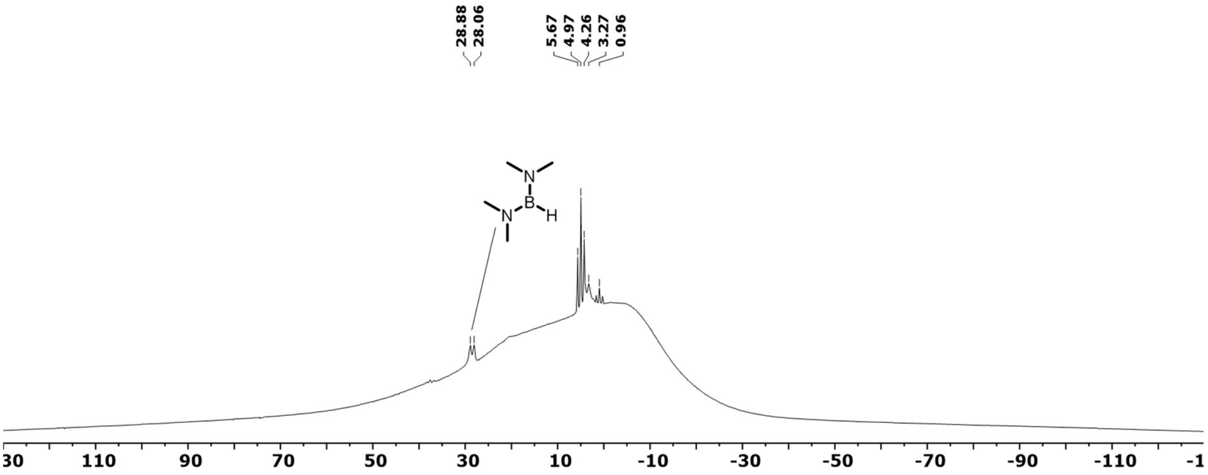

Figure S71: ^{11}B NMR spectrum (160 MHz, C_6D_5Br, 300 K) of the reaction between **1, 4** and diphenylacetylene after 3 days at 70 °C. Only **1** is observed along with trace of its decomposition product $HB(N(CH_3)_2)_2$ (asserted by the doublet at δ 28.5, $^{1}J_{BH}$ = 131 Hz) and trace of by-products from the reaction between **4** and diphenylacetylene.

5.2.3 Hydroboration of diphenylacetylene with (^{i}Pr)$_2$NBHNTf$_2$ 9

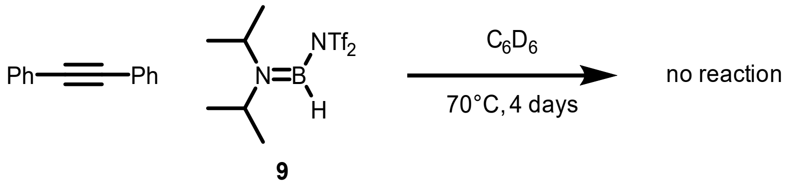

Diphenylacetylene (10 mg, 0.06 mmol, 2.00 equiv.) and (^{i}Pr)$_2$NBHNTf$_2$ **9** (11 mg, 0.03 mmol, 1.00 equiv.) were dissolved in C_6D_6 (0.5 mL) and heated at 70°C for 4 days.

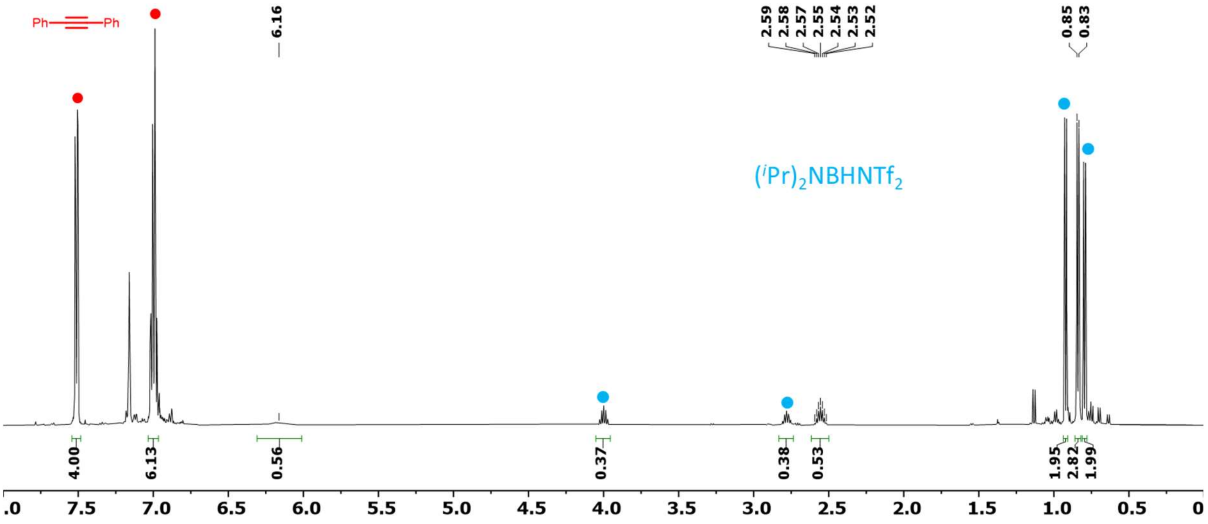

Figure S72: ^{1}H NMR spectrum (500 MHz, C_6D_6, 300 K) of the reaction between **9** and diphenylacetylene after 4 days at 70 °C. Diphenylacetylene (labelled in red) is shown mostly unreacted, while **9** (labelled in blue) is slowly decomposing to an unknown by-product.

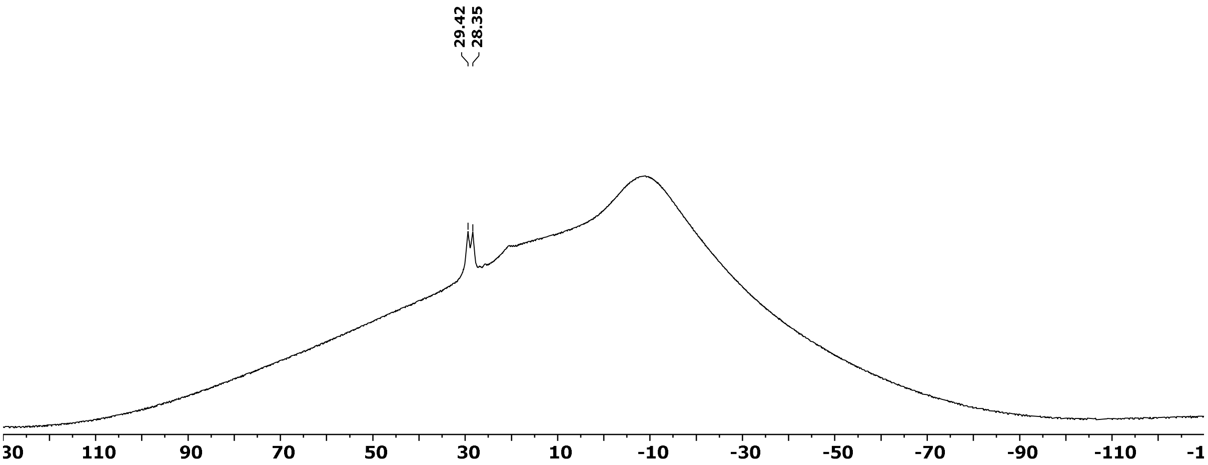

Figure S73: ^{11}B NMR spectrum (500 MHz, C_6D_6, 300 K) of the reaction between **9** and Diphenylacetylene after 4 days at 70 °C. Only **9** is observed confirming the absence of hydroboration of diphenylacetylene and simple decomposition occurring under heating condition.

5.2.4 Hydroboration of diphenylacetylene with (^{i}Pr)$_2$NBH$_2$

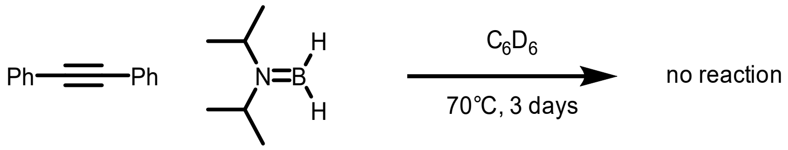

Diphenylacetylene (25 mg, 0.14 mmol, 2.00 equiv.) and (^{i}Pr)$_2$NBH$_2$ (8 mg, 0.07 mmol, 1.00 equiv.) were dissolved in C_6D_6 (0.5 mL) and heated at 70°C for 3 days.

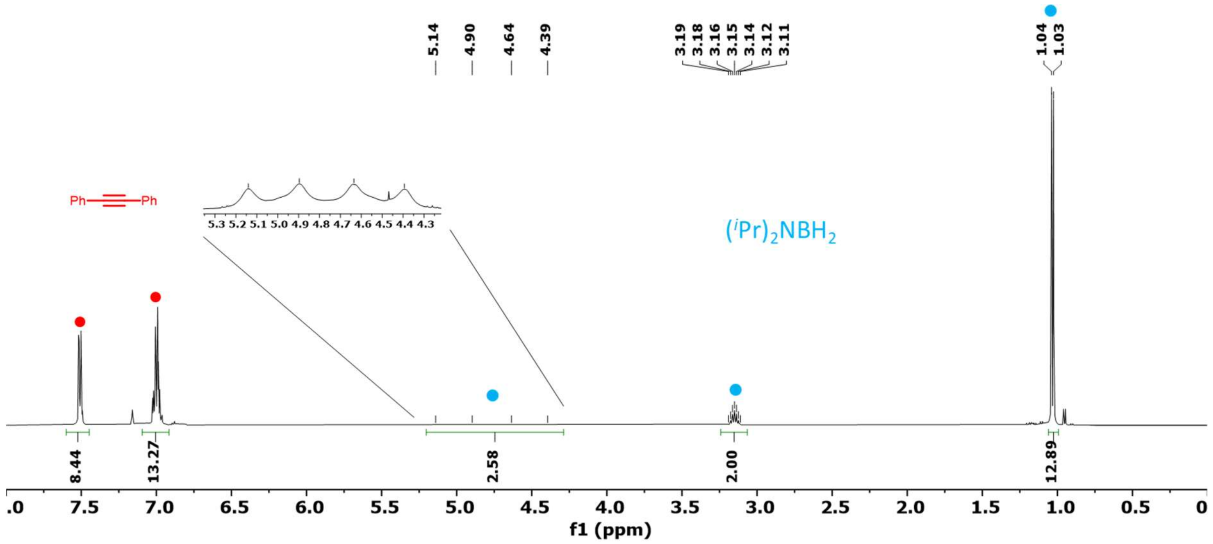

Figure S74: ^{1}H NMR spectrum (500 MHz, C_6D_6, 300 K) of the reaction between (^{i}Pr)$_2$NBH$_2$ and diphenylacetylene after 3 days at 70 °C. Diphenylacetylene and (^{i}Pr)$_2$NBH$_2$ (labelled in red and blue respectively) are shown mostly unreacted.

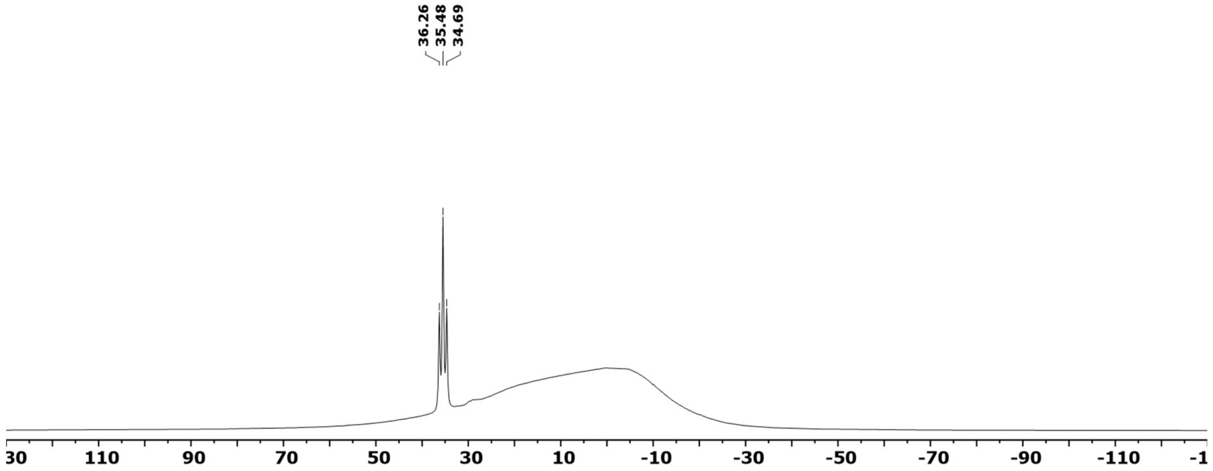

Figure S75: ^{11}B NMR spectrum (500 MHz, C_6D_6, 300 K) of the reaction between (^{i}Pr)$_2$NBH$_2$ and diphenylacetylene after 3 days at 70 °C. Only (^{i}Pr)$_2$NBH$_2$ is observed confirming the absence of hydroboration of diphenylacetylene.

5.3 **Solvent effect**: Hydroboration of Diphenylacetylene with **4 in polar solvent**

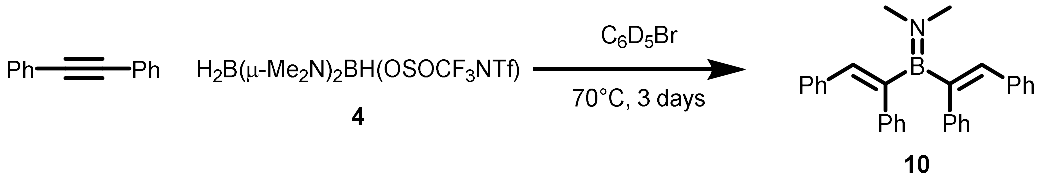

Diphenylacetylene (15 mg, 0.08 mmol, 2.00 equiv.) and $H_2B(\mu\text{-}Me_2N)_2BH(OSOCF_3NTf)$ **4** (16.5 mg, 0.04 mmol, 1.00 equiv.) were dissolved in C_6D_5Br (0.5 mL) in an NMR tube and heated at 70 °C for 3 days.

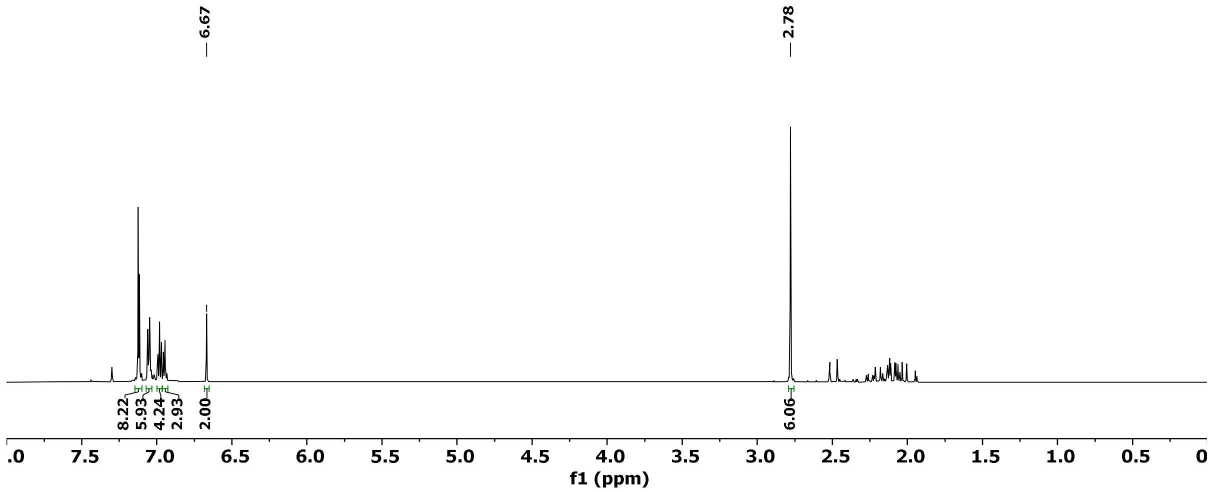

Figure S76: ^{1}H NMR spectrum (500 MHz, C_6D_5Br, 300 K) of the reaction between **4** and diphenylacetylene after 3 days at 70 °C. All the diphenylacetylene is consumed after 3 days and only the product **10** is seen by ^{1}H NMR spectroscopy. The reaction rate increases with the solvent polarity, however the reaction is cleaner in benzene.

5.4 Temperature effect: Hydroboration of Diphenylacetylene with 4 in polar solvent at 40 °C

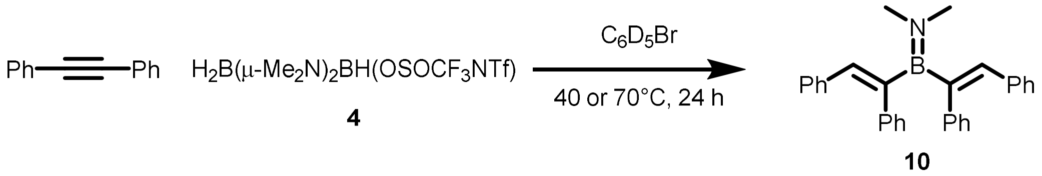

Diphenylacetylene (15 mg, 0.08 mmol, 2.00 equiv.) and $H_2B(\mu\text{-}Me_2N)_2BH(OSOCF_3NTf)$ **4** (16.5 mg, 0.04 mmol, 1.00 equiv.) were dissolved in C_6D_5Br (0.5 mL) in an NMR tube and heated at 40 or 70 °C for 24 hours.

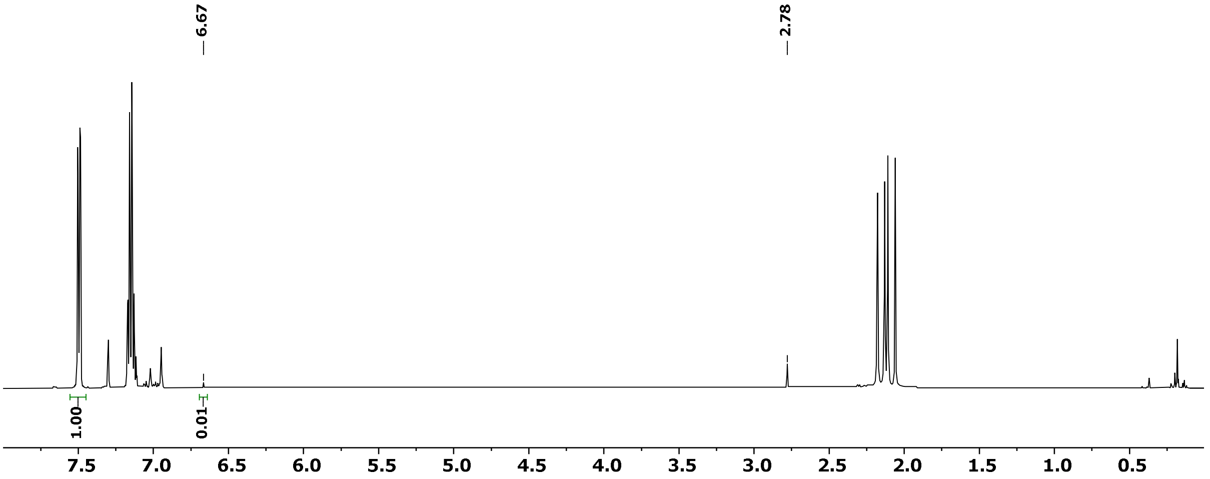

Figure S77: ^{1}H NMR spectrum (500 MHz, C_6D_5Br, 300 K) of the reaction between **4** and Diphenylacetylene after 24 hours at 40 °C. The slow formation of **10** is observed with a ratio of Diphenylacetylene:**10** of 98:2.

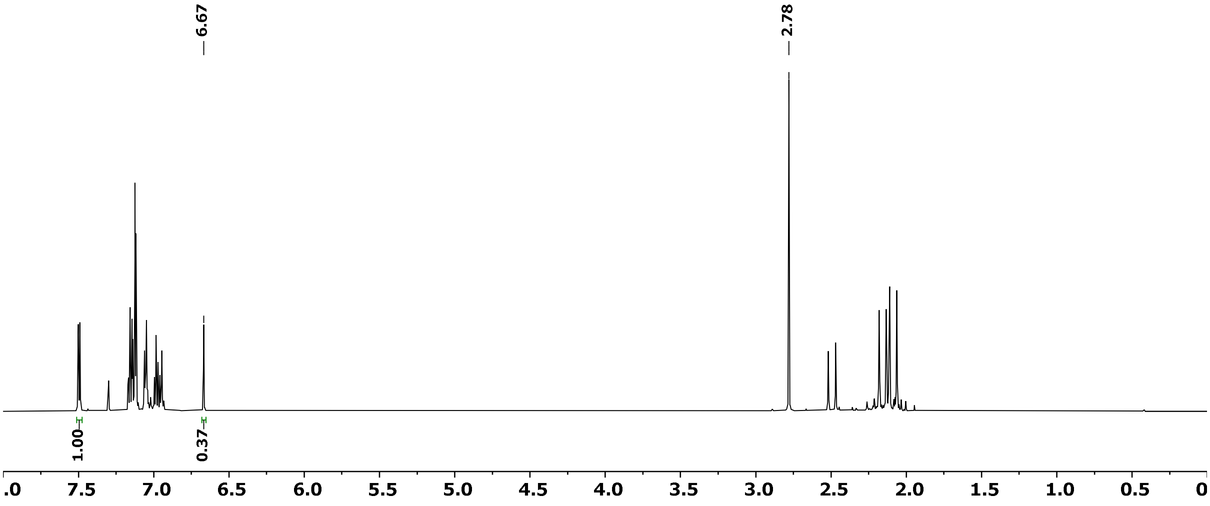

Figure S78: ^{1}H NMR spectrum (600 MHz, C_6D_5Br, 300 K) of the reaction between **4** and diphenylacetylene after 24 hours at 70 °C. The quick formation of **10** is observed with a ratio of diphenylacetylene:**10** of 57:43.

5.5 Study of the By-product from hydroboration reaction

5.5.1 In situ observation of by-product 11

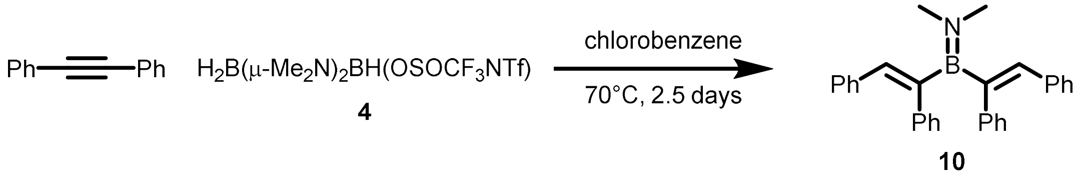

To a solution of diphenylacetylene (0.20 g, 1.12 mmol, 2.00 equiv.) in chlorobenzene (15 mL) was added a solution of $H_2B(\mu\text{-}Me_2N)_2BH(OSOCF_3NTf)$ **4** (0.22 g, 0.56 mmol, 1.00 equiv.) in chlorobenzene (15 mL). The resulting solution was heated at 70 °C for 2.5 days.

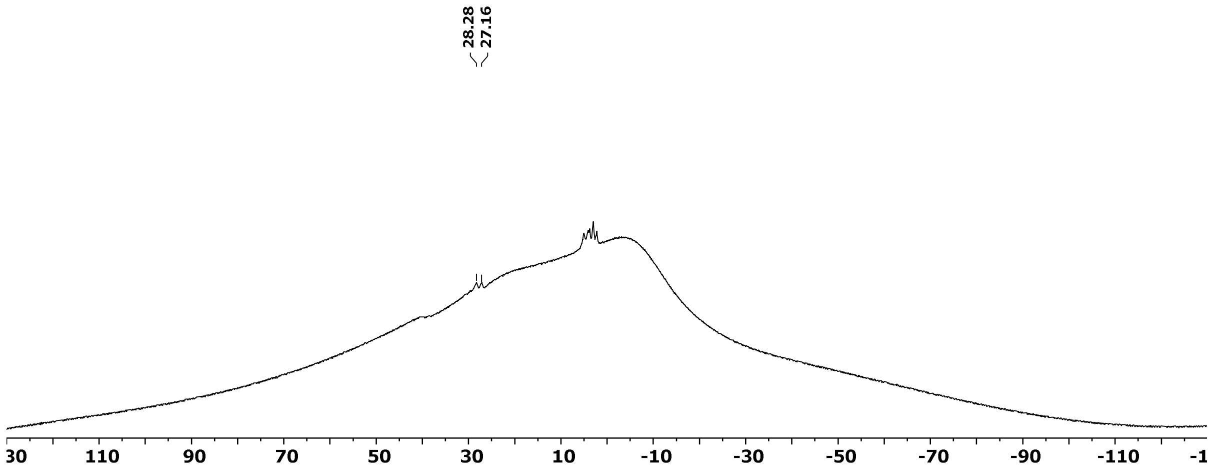

Figure S79: ^{11}B NMR spectrum (160 MHz, Chlorobenzene, 300 K) of the reaction between diphenylacetylene and **4** after 2.5 days at 70 °C. In addition to the signal from starting material **4** and product **10**, a doublet at δ 27.7 ($^1J_{BH}$ = 180 Hz) is observed *in situ*, latter assigned to **11**.

5.5.2 In situ generation of 11 from 1

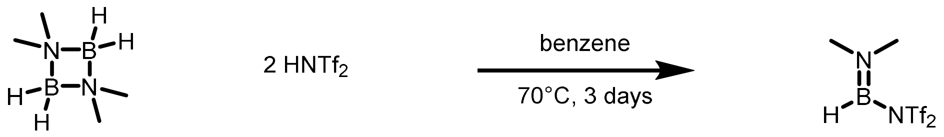

A solution of $HNTf_2$ (0.49 g, 1.75 mmol, 2.00 equiv.) in benzene (5 mL) was slowly added to a solution of $[Me_2NBH_2]_2$ **1** (0.10 g, 0.88 mmol, 1.00 equiv.) in benzene (5 mL) and heated at 70 °C for 3 days.

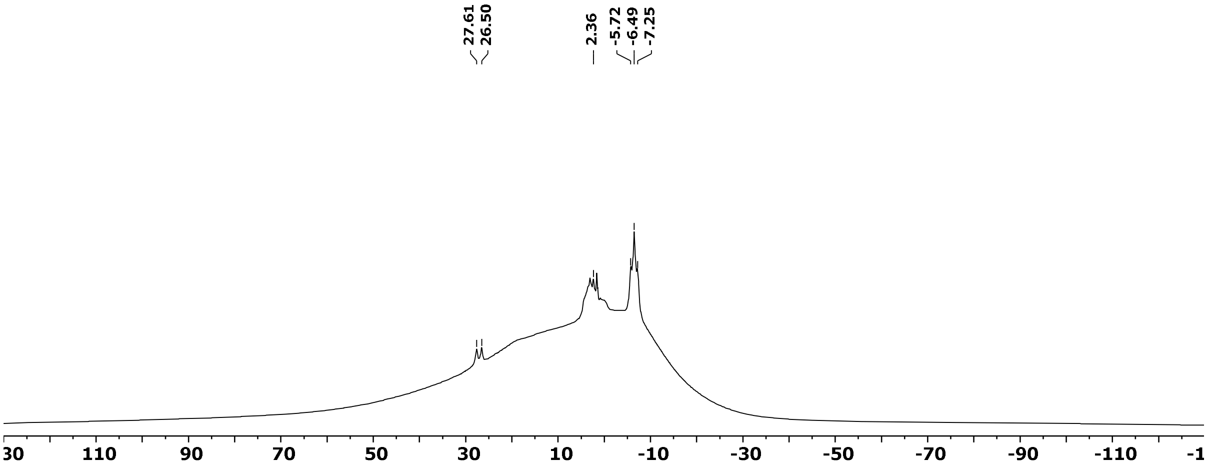

Figure S80: ^{11}B NMR spectrum (160 MHz, benzene, 300 K) of the reaction between $[Me_2NBH_2]_2$ and excess $HNTf_2$ after 3 days at 70 °C. The doublet from **11** is observed at δ 27.1 ($^1J_{BH}$ = 170 Hz) along with other unknown decomposition side-products.

5.5.3 In situ generation of 11 from 4

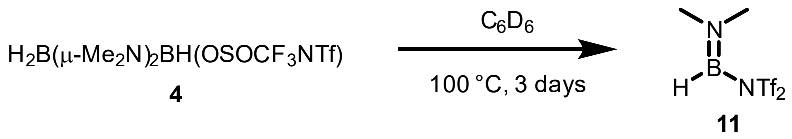

$H_2B(\mu\text{-}Me_2N)_2BH(OSOCF_3NTf)$ **4** (20 mg, 0.05 mmol) was dissolved in C_6D_6 and heated at 100 °C for 3 days.

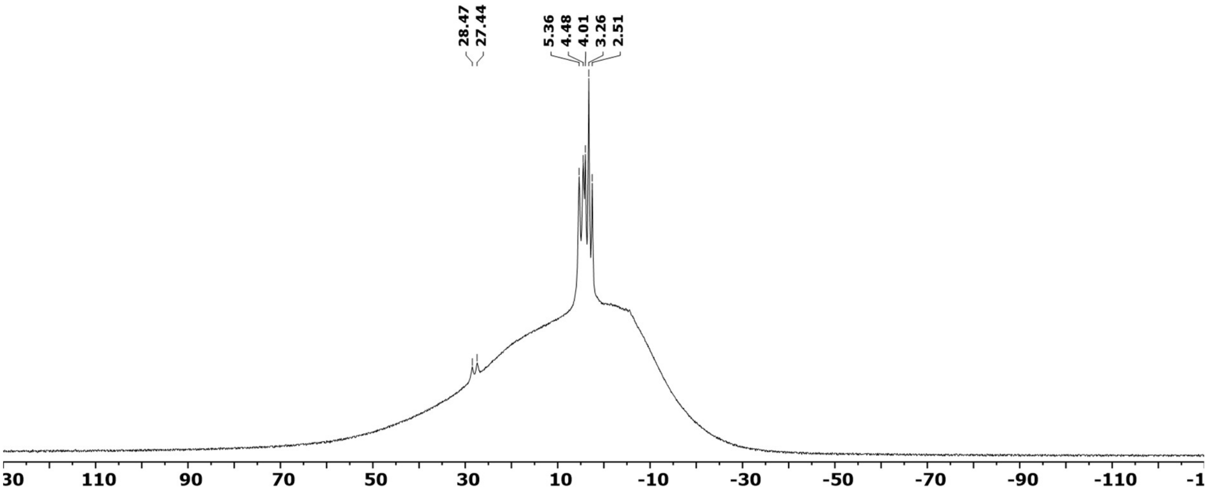

Figure S81: ^{11}B NMR spectrum (160 MHz, C_6D_6, 300 K) of the thermal stability study of **4**. The doublet assigned as **11** is observed at δ 27.9 ($^1J_{BH}$ = 173 Hz).

5.5.4 *Formation of 6*

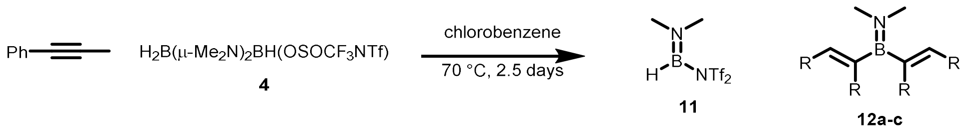

To a solution of $H_2B(\mu\text{-}Me_2N)_2BH(OSOCF_3NTf)$ **4** (0.20 g, 0.50 mmol, 1.00 equiv.) in benzene (2 mL), was added 1-phenyl-1-propyne (130 μL, 1.00 mmol, 2.00 equiv.) using micro syringe. The resulting solution was heated at 70 °C for 1 week. Volatiles were removed under vacuum, affording an oil. The oil was extracted with pentane (2 mL) affording a clear orange solution. The solution was cooled down to 0 °C. Colourless needles started to crystallise out of the solution. After one hour at 0 °C, the crystals were isolated by filtration, affording the by-product **11** in its dimeric form **6** as colourless crystals in extremely small quantity, contaminated with other impurities.

6 Crystallographic data

6.1 Crystal structure of 3

CCDC Deposition Number: 2192971

Experimental: Single colourless plate-shaped crystals of **3** recrystallised by sublimation. A suitable crystal with dimensions 0.20 × 0.09 × 0.05 mm^3 was selected and mounted on a MITIGEN holder in Paratone oil on a Rigaku Oxford Diffraction Excalibur diffractometer equipped with Eos CCD detector. The crystal was kept at a steady T = 120.0(2) K during data collection. The structure was solved with the ShelXS[S8] (Sheldrick, 2008) solution program using dual methods and by using Olex2 1.5-beta[S9] as the graphical interface. The model was refined with ShelXL 2018/3[S10] using full matrix least squares minimisation on F^2.

**Compound**	**3**
Formula	$C_4H_{15}B_2IN_2$
$D_{calc.}$/ g cm^{-3}	1.669
μ/mm^{-1}	3.286
Formula Weight	239.70
Colour	colourless
Shape	plate-shaped
Size/mm^3	0.202x0.095x0.051
T/K	120.0(2)
Crystal System	orthorhombic
Space Group	Cmce
a/Å	8.57117(14)
b/Å	11.8624(2)
c/Å	18.7668(3)
α/°	90
β/°	90
γ/°	90
V/Å^3	1908.11(5)
Z	8
Wavelength/Å	0.71073
Radiation type	Mo K_α
Θ_{min}/°	6.87
Θ_{max}/°	58.798
Measured Refl's.	20187
Indep't Refl's	1333
R_{int}	0.0402
Parameters	57
Restraints	0
Largest Peak	1.03
Deepest Hole	-0.42
GooF	1.144
wR_2 (all data)	0.0534
wR_2	0.0505
R_1 (all data)	0.0363
R_1	0.0268

6.2 Crystal structure of 6

CCDC Deposition Number: 2192968

Experimental: Single colourless plate-shaped crystals of **6** recrystallised from pentane by slow cooling. A suitable crystal with dimensions 0.50 × 0.23 × 0.12 mm^3 was selected and mounted on a MITIGEN holder in Paratone oil on a Rigaku Oxford Diffraction XCalibur diffractometer. The crystal was kept at a steady T = 120.00(10) K during data collection. The structure was solved with the ShelXT 2018/2[S11] solution program using dual methods and by using Olex2 1.5-beta[S9] as the graphical interface. The model was refined with ShelXL 2018/3[S10] using full matrix least squares minimisation on F^2.

**Compound**	**6**
Formula	$C_8H_{14}B_2F_{12}N_4O_8S_4$
$D_{calc.}$/ g cm^{-3}	1.858
μ/mm^{-1}	0.533
Formula Weight	672.09
Colour	colourless
Shape	plate-shaped
Size/mm^3	0.50×0.23×0.12
T/K	120.00(10)
Crystal System	monoclinic
Space Group	$P2_1/c$
a/Å	8.1082(6)
b/Å	19.4886(11)
c/Å	8.5332(7)
α/°	90
β/°	117.024(10)
γ/°	90
V/Å^3	1201.17(18)
Z	2
Z'	0.5
Wavelength/Å	0.71073
Radiation type	Mo K_α
Θ_{min}/°	3.399
Θ_{max}/°	27.103
Measured Refl's.	13881
Indep't Refl's	2647
Refl's I≥2 σ(I)	2186
R_{int}	0.0274
Parameters	335
Restraints	315
Largest Peak	0.431
Deepest Hole	-0.571
GooF	1.061
wR_2 (all data)	0.1742
wR_2	0.1651
R_1 (all data)	0.0845
R_1	0.0710

6.3 **Crystal structure of 7**

CCDC Deposition Number: 2192969

Experimental: Single colourless crystals of **7** were obtained by layering C_6H_5Cl with pentane. A suitable crystal with dimensions 0.162 x 0.133 x 0.09 mm^3 was selected and mounted on a MITIGEN holder in Paratone oil on a Rigaku Oxford Diffraction SuperNova diffractometer. The crystal was kept at a steady T = 120.01(10) K during data collection. The structure was solved with the ShelXT[S11] solution program using dual methods and by using Olex2 1.5-beta[S9] as the graphical interface. The model was refined with ShelXL 2018/3[S10] using full matrix least squares minimisation on F^2.

**Compound**	**7**
Formula	$C_{30}H_{23}B_4F_{20}N_3$
$D_{calc.}$/ g cm^{-3}	1.679
μ/mm^{-1}	1.591
Formula Weight	848.75
Colour	colourless
Shape	block
Size/mm^3	0.162x0.133x0.09
T/K	120.01(10)
Crystal System	triclinic
Space Group	P-1
a/Å	8.9462(2)
b/Å	13.1696(4)
c/Å	15.0006(4)
α/°	77.036(2)
β/°	86.572(2)
γ/°	77.166(2)
V/Å^3	1679.17(8)
Z	2
Wavelength/Å	1.54184
Radiation type	Cu K_α
Θ_{min}/°	7.052
Θ_{max}/°	152.822
Measured Refl's.	55243
Indep't Refl's	6969
R_{int}	0.0712
Parameters	538
Restraints	0
Largest Peak	1.33
Deepest Hole	-0.35
GooF	1.052
wR_2 (all data)	0.1594
wR_2	0.1550
R_1 (all data)	0.0578
R_1	0.0545

6.4 **Crystal structure of 9**

CCDC Deposition Number: 2192967

Experimental: Single colourless plate-shaped crystals of **9** recrystallised by sublimation. A suitable crystal with dimensions 0.30 × 0.15 × 0.05 mm^3 was selected and mounted on a MITIGEN holder in Paratone oil on a Rigaku Oxford Diffraction SuperNova diffractometer. The crystal was kept at a steady T = 100.0 K during data collection. The structure was solved with the ShelXT[S11] solution program using dual methods and by using Olex2 1.5-beta[S9] as the graphical interface. The model was refined with ShelXL 2018/3[S10] using full matrix least squares minimisation on F^2.

**Compound**	**9**
Formula	$C_8H_{15}BF_6N_2O_4S_2$
$D_{calc.}$/ g cm^{-3}	1.577
μ/mm^{-1}	3.695
Formula Weight	392.15
Colour	colourless
Shape	plate-shaped
Size/mm^3	0.30×0.15×0.05
T/K	100.0
Crystal System	monoclinic
Space Group	$P2_1/c$
a/Å	13.0578(4)
b/Å	9.3579(2)
c/Å	14.0921(4)
α/°	90
β/°	106.392(3)
γ/°	90
V/Å^3	1651.97(8)
Z	4
Z'	1
Wavelength/Å	1.54184
Radiation type	Cu K_α
Θ_{min}/°	3.528
Θ_{max}/°	76.193
Measured Refl's.	30965
Indep't Refl's	6870
Refl's I≥2 σ(I)	6280
R_{int}	0.0983
Parameters	312
Restraints	308
Largest Peak	0.565
Deepest Hole	-0.597
GooF	1.028
wR_2 (all data)	0.1628
wR_2	0.1598
R_1 (all data)	0.0606
R_1	0.0575

6.5 **Crystal Structure of [(4-DMAP)$_2$BH$_2$][NTf$_2$]**

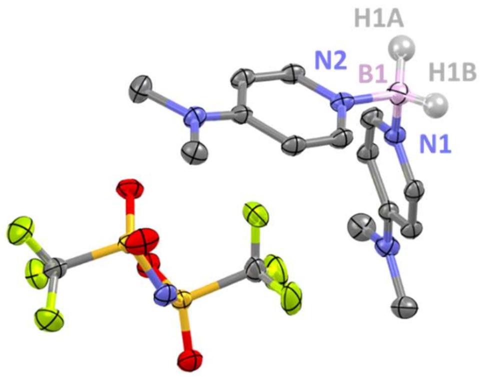

Figure S82: Solid-state Structure of [(4-DMAP)$_2$BH$_2$][NTf$_2$] (50% ellipsoid probability). Hydrogen atoms except H1A and H1B are omitted for clarity.

CCDC Deposition Number: 2192970

Experimental: Single colourless plate-shaped crystals of [(4-DMAP)2BH2][NTf2] recrystallised from hexane by slow evaporation. A suitable crystal with dimensions 0.08 × 0.06 × 0.03 mm^3 was selected and mounted on a MITIGEN holder in Paratone oil on a Rigaku Oxford Diffraction SuperNova diffractometer. The crystal was kept at a steady T = 120.01(10) K during data collection. The structure was solved with the ShelXT 2014/5[S11] solution program using dual methods and by using Olex2 1.5-beta[S9] as the graphical interface. The model was refined with ShelXL 2018/3[S10] using full matrix least squares minimisation on F^2.

**Compound**	**[(4-DMAP)$_2$BH$_2$][NTf$_2$]**
Formula	$C_{16}H_{22}BF_6N_5O_4S_2$
$D_{calc.}$/ g cm^{-3}	1.548
μ/mm^{-1}	2.863
Formula Weight	537.31
Colour	colourless
Shape	plate-shaped
Size/mm^3	0.08×0.06×0.03
T/K	120.01(10)
Crystal System	triclinic
Space Group	P-1
a/Å	8.6886(4)
b/Å	8.7838(4)
c/Å	15.5534(8)
α/°	78.847(4)
β/°	85.669(4)
γ/°	82.484(4)
V/Å^3	1153.06(10)
Z	2
Z'	1
Wavelength/Å	1.54184
Radiation type	CuK$_\alpha$
Θ_{min}/°	5.142
Θ_{max}/°	76.209
Measured Refl's.	18803
Indep't Refl's	4756
Refl's I≥2 σ(I)	3894
R_{int}	0.0763
Parameters	320
Restraints	0
Largest Peak	0.525
Deepest Hole	-0.518
GooF	1.038
wR_2 (all data)	0.1614
wR_2	0.1501
R_1 (all data)	0.0702
R_1	0.0586

7 Computational Details

All calculations were performed using the Gaussian09 programme.[S12] Geometries optimisation were completed with the DFT method using the M06-2X functional[S13] and the 6-311G(d,p) as a basis set. All geometry optimizations were full, with no restrictions. Stationary points located in the potential energy surface were characterized as minima by vibrational analysis. Solvent effects of the dichloromethane were introduced using the self consistent field approach, by means of the integral equation formalism polarizable continuum model (IEFPCM).[S14]

8 References

S1. Ihara E., Young, Jr. V. G. , Jordan R. F.; Cationic Aluminum Alkyl Complexes Incorporating Aminotroponiminate ligands. *J. Am. Chem. Soc.* **1998**, 120, 8277-8278.

S2. Orr S. A., Kennedy A. R., Liggat J. J., McLellan R., Mulvey R. E., Robertson S.D.; Accessible heavier s-block dihydropyridines: structural elucidation and reactivity of isolable molecular hydride sources. *Dalton Trans.* **2016**, 45, 6234-6240.

S3. Vance J. R., Robertson A. P. M., Lee K., Manners I.; Photoactivated, Iron-Catalyzed Dehydrocoupling of Amine-Borane Adducts: Formation of Boron-Nitrogen Oligomers and Polymers. *Chem. Eur. J.* **2011**, 17, 4099-4103.

S4. Vance J. R., Schäfer A., Robertson A. P. M., Lee K., Turner J., Whittell G. R., Manners I.; Iron-Catalyzed Dehydrocoupling/Dehydrogenation of Amine − Boranes. *J. Am. Chem. Soc.* **2014**, 136, 3048-3064.

S5. Jaska C. A., Temple K., Lough A. J., Manners I.; Rhodium-catalyzed formation of boron-nitrogen bonds: A mild route to cyclic aminoboranes and borazines. *Chem. Commun.* **2001**, 11, 962-963.

S6. Dazord J., Mongeot H., Atchekzaï H., Tuchagues J.P.; Preparation and chemico-physical study of halogeno-monoboranes containing the cyanate or thiocyanate group and related compounds $R_3N{:}BX_n(NCS)_{3-n}$. *J. Less-Common Metals* **1976**, 47, 55-59.

S7. Nöth H., Lukas S.; Die Reaktion von Chlorwasserstoff mit Tris(dialkylamino)-boranen. Das Salz $[Cl_2B(HNMe_2)_2]Cl$. *Chem Ber.* **1962**, 95, 1505-1512.

S8. Sheldrick G. M.; A short history of SHELX. *Acta Cryst.* **2008**, A64, 112-122.

S9. Dolomanov O. V., Bourhis L. J., Gildea R. J., Howard J. A. K., Puschmann H.; OLEX2: A complete structure solution, refinement and analysis program. *J. Appl. Cryst.* **2009**, 42, 339-341.

S10. Sheldrick G.M.; Crystal structure refinement with SHELXL. *Acta Cryst.* **2015**, C71, 3-8.

S11. Sheldrick G.M.; SHELXT - Integrated space-group and crystal-structure determination. *Acta Cryst.* **2015**, A71, 3-8.

S12. Gaussian 09, Revision C1; Frisch M. J., Trucks G. W., Schlegel H. B., Scuseria G. E., Robb M. A., Cheeseman J. R., Scalmani G., Barone V., Mennucci B., Petersson G. A., Nakatsuji H., Caricato M., Li X., Hratchian H. P., Izmaylov A. F., Bloino J., Zheng G., Sonnenberg J. L., Hada M., Ehara M., Toyota K., Fukuda R., Hasegawa J., Ishida M., Nakajima T., Honda Y., Kitao O., Nakai H., Vreven T., Montgomery Jr. J. A., Peralta J. E., Ogliaro F., Bearpark M., Heyd J. J., Brothers E., Kudin K. N., Staroverov V. N., Kobayashi R., Normand J., Raghavachari K., Rendell A., Burant J. C., Iyengar S. S., Tomasi J., Cossi M., Rega N., Millam J. M., Klene M., Knox J. E., Cross J. B., Bakken V., Adamo C., Jaramillo J., Gomperts R., Stratmann R. E., Yazyev O., Austin A. J., Cammi R., Pomelli C., Ochterski J. W., Martin R. L., Morokuma K., Zakrzewski V. G., Voth G. A., Salvador P., Dannenberg J. J., Dapprich S., Daniels A. D., Farkas Ö., Foresman J. B.,

Ortiz J. V., Cioslowski J., Fox D. J. Gaussian, Inc., Wallingford CT, 2009.

S13. Zhao Y., Truhlar D. G.; *Theor. Chem. Acc*. **2008**, 120, 215-241.

S14. Mennucci B., Cancès E., Tomasi J.; *J. Phys. Chem. B* **1997**, 101, 10506-10517.
